# ﻿Distribution patterns of *Calonectria* (Ascomycota, Sordariomycetes, Hypocreales, Nectriaceae) species complexes related to diseased leaves and soil habitats during leaf blight outbreak season in *Eucalyptus* plantations in southern China

**DOI:** 10.3897/mycokeys.110.130733

**Published:** 2024-11-04

**Authors:** WenXia Wu, ShuaiFei Chen

**Affiliations:** 1 Research Institute of Fast-growing Trees (RIFT), Chinese Academy of Forestry (CAF), Zhanjiang, 524022, Guangdong Province, China Research Institute of Fast-growing Trees (RIFT), Chinese Academy of Forestry (CAF) Zhanjiang China

**Keywords:** Calonectria leaf blight, *Eucalyptus* disease, forest pathogens, fungal ecology, phylogeny

## Abstract

Calonectria leaf blight caused by *Calonectria* species is one of the most important diseases associated with *Eucalyptus* plantations in Asia and South America. This study aimed to clarify the distribution patterns of *Calonectria* species residing in different species complexes associated with diseased trees and soils during leaf blight outbreak season in *Eucalyptus* plantations in southern China. In this study, 482 *Calonectria* isolates obtained from diseased *Eucalyptus* trees and soils under these trees in eight sampling sites in three provinces were identified by DNA sequence analyses of *tef1*, *tub2*, *cmdA*, and *his3* gene regions. Six species residing in three species complexes were identified: *Calonectriapseudoreteaudii* and *C.acaciicola* in the *Calonectriareteaudii* species complex; *C.hongkongensis*, *C.aconidialis*, and *C.chinensis* in *C.kyotensis* species complex; and *C.auriculiformis* in *C.cylindrospora* species complex. The habitats of *Calonectria* in different species complexes differed, *C.reteaudii* species complex inhabits in both diseased trees and soils, *C.kyotensis* species complex only in soils. The Calonectria leaf blight in the sampled regions was caused by species in the *C.reteaudii* species complex but not by the species in the *C.kyotensis* species complex. These findings suggest that the species in the *C.reteaudii* species complex should receive more attention in disease management, as they are the primary cause of the disease in the sampled regions.

## ﻿Introduction

The eucalypts, commonly known as gum trees, include the genera *Angophora*, *Corymbia*, and *Eucalyptus*, with more than 800 species, of which *Eucalyptus* spp. are the most numerous (Thornhill et al. 2019). *Eucalyptus* species are widely cultivated as commercial trees in Southeast Asia, Brazil, China, Australia, India, Europe, and South Africa due to their ability of fast-growing, adaptability, and versatility (Xie and Du 2019). In China, *Eucalyptus* plantations cover an area of approximately 5.46 million hm^2^, accounting for approx. 2.5% of the country’s total forest area, and provide one-third of the country’s timber production (Xie et al. 2017; Xie and Du 2019).

The Eucalyptus leaf blight, caused by *Calonectria* spp., is considered one of the most severe diseases affecting *Eucalyptus* plantations, especially in Asia and South America (Crous 2002; Rodas et al. 2005; Alfenas et al. 2015; Pham et al. 2019, 2022a, 2022b; Wang and Chen 2020; Li et al. 2023a; Liang et al. 2023). The disease initially presents as water-soaked and light gray lesions of the middle and lower leaves. As it rapidly spreads, the lesions progress to light brown color and cover a significant portion of the leaf blade, ultimately leading to defoliation and even the death of the entire tree under highly favorable environmental conditions (Old et al. 2003; Rodas et al. 2005; Wang and Chen 2020; Wu and Chen 2021; Liang et al. 2023).

Severe outbreaks of Calonectria leaf blight have significantly impacted the growth of *Eucalyptus* plantations in southern China, resulting in substantial economic losses (Zhou and Wingfield 2011; Wang and Chen 2020; Wu and Chen 2021; Li et al. 2023a; Liang et al. 2023). The disease was initially observed in an *Eucalyptus* nursery located in Hainan Province in 1985, which resulted in significant mortality among *Eucalyptus* seedlings (Feng and Zheng 1986). In recent years, leaf blight caused by *Calonectria* species has been observed and confirmed in plantation in Fujian, Guangdong, Guangxi, and Hainan Provinces (Chen et al. 2013; Wang and Chen 2020; Wu and Chen 2021; Li et al. 2023b; Liang et al. 2023). The *Calonectria* species that are frequently isolated from diseased *Eucalyptus* trees in China include *C.acaciicola*, *C.pseudoreteaudii*, and *C.queenslandica* (Wang and Chen 2020; Wu and Chen 2021; Li et al. 2023a; Liang et al. 2023).

*Calonectria* has been detected not only in *Eucalyptus* tissues (mainly from tree leaves, as well as tree shoots and seedling stems) but also in soils under diseased trees and seedlings in China (Li et al. 2017, 2023a; Wang and Chen 2020; Liang et al. 2023). Presently, 26 *Calonectria* species associated with *Eucalyptus* have been identified and reported, including 19 species isolated from diseased leaves, 17 species isolated from soils associated with *Eucalyptus*, and 10 species isolated from both diseased tissues and soils associated with *Eucalyptus* (Lombard et al. 2010c; Li et al. 2017; Liu et al. 2020, 2021; Wu and Chen 2021; Zhang et al. 2022; Liang et al. 2023; Liu and Chen 2023).

*Calonectria* species have been frequently isolated from diseased *Eucalyptus* trees and soils in their plantations (Liu et al. 2020, 2021; Wu and Chen 2021; Li et al. 2023b; Liu and Chen 2023). However, only two studies were conducted to understand the species diversity and distribution characteristics of *Calonectria*, both from diseased plantation *Eucalyptus* trees and soils under these trees (Wu and Chen 2021; Li et al. 2023b). These studies were conducted solely in one *Eucalyptus* plantation (Wu and Chen 2021), or in a limited number of sampling regions, and there are significant differences in the number of samples between diseased trees and the soil under these trees (Li et al. 2023b). As a result, the distribution patterns of *Calonectria* species associated with diseased *Eucalyptus* trees and soils under these trees are still unclear. The purpose of this study was to comprehensively understand the distribution characteristics of *Calonectria* species related to diseased leaves and soil habitats during leaf blight outbreak season in *Eucalyptus* plantations in southern China. This was achieved by systematically procuring sampled collections of *Calonectria* from eight Calonectria leaf blight outbreak *Eucalyptus* plantations in three provinces in southern China.

## ﻿Materials and methods

### ﻿Disease survey, sample collection, and fungal isolation

**Figure 1.**
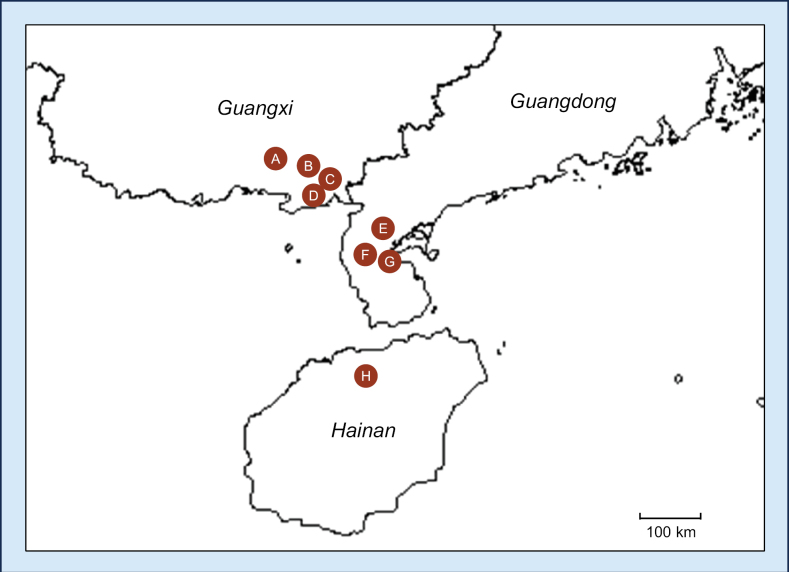
Locations of the eight sampled plantations in the Guangxi (sites **A, B, C, D**), Guangdong (sites **E, F, G**), and Hainan (site **H**) provinces in southern China.

**Figure 2.**
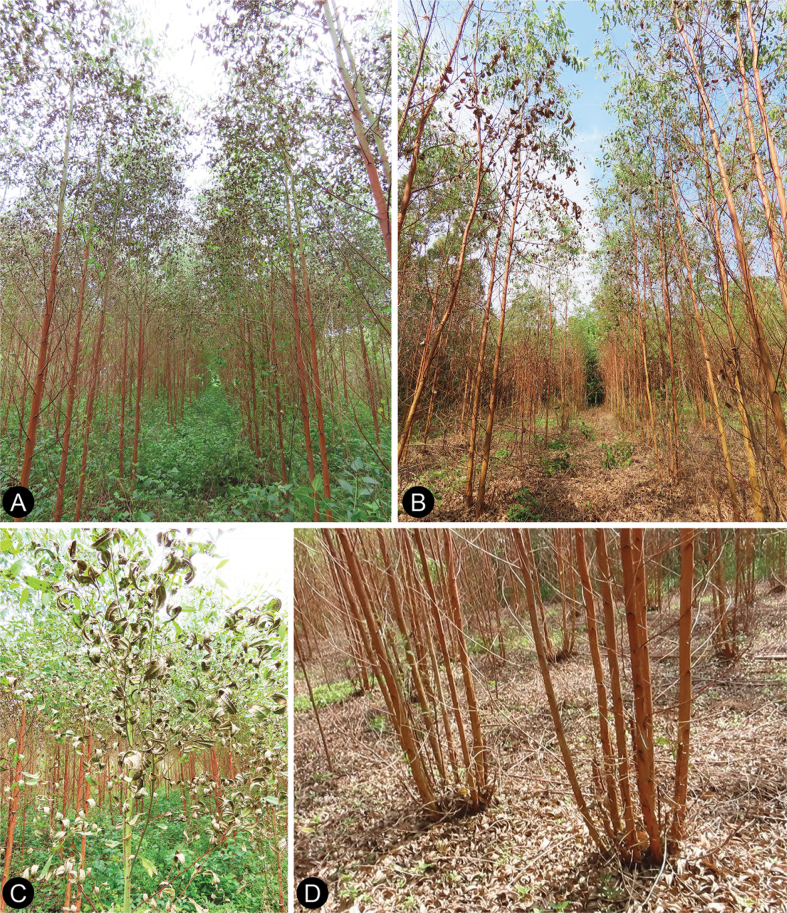
Diseased leaves and soil samples at plantations of a *Eucalyptusurophylla* hybrid in southern China **A, B** leaf spot and blight caused by *Calonectria* species were observed on 60%–80% of the trees in the plantations **C** blighted and dried dead leaves **D** leaves that were dying and drying, resulting in defoliation in the plantation.

In September 2021, we conducted several extensive surveys of the disease caused by *Calonectria* species in *E.urophylla* hybrid plantations in Guangdong, Guangxi, and Hainan Provinces in southern China. After the surveys, eight plantations were selected for sampling (Fig. 1). At each of the eight plantations, the trees were one-year-old, and the disease of *Calonectria* leaf blight occurred for about a month. The disease symptoms of Calonectria leaf blight were observed on 60%–80% of trees in each plantation (Fig. 2A, B) typically included leaf spots and blight (Fig. 2C), which resulted in defoliation (Fig. 2D). Depending on the plantation area, a number of diseased trees were selected for diseased leaf sampling. These trees were randomly distributed across each plantation. Three to five fresh symptomatic leaves were collected from each sampled tree. The same number of soil samples was collected under it from the upper 0–20 cm soil profile by removing the thick layer of leaf litter, as described by Liu and Chen (2023). The diseased leaf and soil samples were taken to our laboratory for further study.

To induce *Calonectria* sporulation on leaf samples, one diseased leaf of each sampled tree with typical symptoms of Calonectria leaf blight was selected for incubation in a moist petri dish chamber at room temperature until the conidiophores were observed. The development of *Calonectria* strains in soil samples was induced by using *Medicagosativa* (alfalfa) seeds, as described by Liu and Chen (2023). The single conidia from conidial masses of *Calonectria* that sporulated from the diseased leaf or soil samples were transferred to 2% (v/v) malt extract agar (MEA) also following Wu and Chen’s protocol (2023). One isolate with typical morphological characteristics of the conidiophores of *Calonectria*, was isolated from each diseased leaf sample or soil sample. Occasionally, two *Calonectria* isolates were isolated from each sample when the isolates with different morphological vesicles were observed. All obtained single conidium cultures were deposited in the Culture Collection (CSF) at the Research Institute of Fast-growing Trees (RIFT), Chinese Academy of Forestry (CAF), Zhanjiang, Guangdong Province, China.

### ﻿DNA extraction, PCR amplification, and sequencing

The obtained *Calonectria* isolates were cultivated in a 2% MEA medium for a week at room temperature for total genomic DNA extraction. The mycelia were scraped from the cultures. The total genomic DNA of each isolate was extracted using the cetyltrimethylammonium bromide (CTAB) method, as described by van Burik et al. (1998). Four gene regions, namely the translation elongation factor 1-alpha (*tef1*), β-tubulin (*tub2*), calmodulin (*cmdA*), and histone H3 (*his3*), were amplified using the primer pairs and PCR protocols described by Liu et al. (2020). All PCR products were Sanger sequenced in both directions by the same primers used for PCR amplification. The PCR products were sequenced by the Beijing Genomics Institute, Guangzhou, China. All initial sequences were edited using Geneious v.7.1.8 (Kearse et al. 2012). The sequences obtained in this study were deposited in GenBank (http://www.ncbi.nlm.nih.gov).

### ﻿Phylogenetic analyses

The *tef1* and *tub2* regions were sequenced for all *Calonectria* isolates selected for identification in the current study. All these isolates were preliminarily identified through standard nucleotide BLAST searches in the NCBI database (https://blast.ncbi.nlm.nih.gov/) using the *tef1* and *tub2* sequences. Based on their preliminary identification, representative isolates were selected for sequencing the additional gene regions of *cmdA* and *his3*. Based on the combined genotype of *tef1*, *tub2*, *cmdA*, and *his3* sequences, representative isolates presenting all genotypes obtained in this study were used for molecular identification. Sequences of the isolates from the type specimens of all the published species in the preliminarily identified *Calonectria* species complexes were used for phylogenetic analyses. The sequence datasets were aligned using online MAFFT v. 7 (http://mafft.cbrc.jp/alignment/server/) with the FFT-NS-i strategy (slow; interactive refinement method) (Katoh and Standley 2013). The aligned sequence datasets were manually edited and cut using MEGA v. 7.0 software (Kumar et al. 2016).

The sequence datasets of each gene region and the combination of four gene regions were performed on Maximum likelihood (ML) and Bayesian inference (BI) phylogenetic analyses using CIPRES Science Gateway v. 3.3. For the BI analyses, the most suitable models of the five sequence databases were carried through the jModelTest v. 2.1.5 (Posada 2008). Both ML and BI analyses were completed using online software, RaxML v. 8.2.12 (Stamatakis 2014) and MrBayes. v. 3.2.7 (Ronquist et al. 2012), respectively, as described by Wu and Chen (2023). Phylogenetic trees were viewed via FigTree v 1.4.2 and MEGA v. 7 for BI and ML trees, respectively.

## ﻿Results

### ﻿Sample collection and fungal isolation

After a comprehensive collection of samples across eight sites (A–H) in Guangdong, Guangxi, and Hainan Provinces of southern China, a total of 802 samples were collected. These included 401 diseased leaf samples from 401 trees, and 401 soil samples (Table 1, Figs 1, 2). At each sampling site, 39–62 diseased leaf samples, and the same number of soil samples were collected.

**Table 1.** The number of samples and *Calonectria* spp. obtained from *Eucalyptus* plantations at eight sampling sites.

Site code	Diseased leaf samples						Soil samples									
	Total number of diseased leaf samples	No. of samples which yielded *Calonectria*	Total number of *Calonectria* isolates	No. of *Calonectria* isolates which selected for sequencing	No. of *C.pseudoreteaudii* isolates	No. of *C.acaciicola* isolates	Total number of soil samples	No. of samples obtained *Calonectria*	Total number of *Calonectria* isolates	No. of *Calonectria* isolates which selected for sequencing	No. of *C.pseudoreteaudii* isolates	No. of *C.acaciicola* isolates	No. of *C.hongkongensis* isolates	No. of *C.aconidialis* isolates	No. of *C.chinensis* isolates	No. of *C.auriculiformis* isolates
A	62	61	61	20	20	0	62	47	53	26	20	0	0	6	0	0
B	50	50	50	50	50	0	50	19	19	19	19	0	0	0	0	0
C	50	50	50	20	20	0	50	34	35	21	20	0	0	1	0	0
D	50	50	50	50	50	0	50	30	31	31	29	0	2	0	0	0
E	50	50	50	50	50	0	50	26	26	26	25	0	0	0	0	1
F	39	39	39	20	20	0	39	5	5	5	5	0	0	0	0	0
G	50	49	49	20	20	0	50	1	1	1	1	0	0	0	0	0
H	50	50	54	54	33	21	50	50	69	69	29	21	14	0	5	0
Total	401	399	403	284	263	21	401	212	239	198	148	21	16	7	5	1

*Calonectria* isolates were obtained from leaf samples from the diseased trees from the eight sampling sites, except for one of the 62 samples from site A and one of the 50 samples from site G. The proportion of diseased leaf samples successfully obtained from *Calonectria* ranged from 98% to 100% for the eight sampling sites (avg. 99.5%). Each isolate was obtained from a single *Calonectria* diseased leaf sample, except for 54 *Calonectria* isolates from 50 such samples at site H. This is because four diseased leaf samples at site H exhibited differing vesicle morphologies, therefore, two isolates were obtained from each of them (Table 1).

A relatively large proportion of *Calonectria* isolates were obtained from the soil samples from the eight sites, except for sites F and G (Guangdong Province). The proportion of soil samples with *Calonectria* ranged from 2% (site G) to 100% (site H) (avg. 51.1%). Each isolate was obtained from a single *Calonectria* soil sample at sites B, E, F, and G, while more than one isolate was obtained from some of the soil samples at the sites A, C, D, and H where isolates with different morphologies of the conidia or vesicles were observed (Table 1).

In total, 642 isolates with typical morphological characteristics of *Calonectria* were obtained. These included 403 isolates from the 401 diseased leaf samples and 239 isolates from the 401 soil samples (Table 1).

### ﻿Sequencing

*Calonectria* isolates were obtained from a relatively large proportion of the samples, from both the diseased trees and soils, at sites A, B, C, D, E, and H. All isolates from sites B, D, E, and H were sequenced. Since sites A, and C are relatively near to B, and D, only partial isolates obtained from diseased trees and soils at sites A, and C were sequenced. Since *Calonectria* was obtained from a small proportion of the soil samples at sites F, and G, all the isolates obtained from soils, and partial isolates obtained from diseased trees at the two sites were sequenced (Table 1). In total, 482 isolates were used to sequence the *tef1* and *tub2* gene regions (Table 1, Suppl. material 1). This included 54 isolates that were also identified by Liang et al. (2023) (Suppl. material 1). Based on the combined genotypes of the *tef1* and *tub2* gene sequences, and the sampling source, 169 isolates were selected for further sequencing of the *cmdA* and *his3* gene regions (Suppl. material 1). A total of 18 genotypes were generated based on the sequences of the *tef1*, *tub2*, *cmdA*, and *his3* regions of the 169 isolates that allowed for their identification. The remaining 313 isolates were identified based on *tef1* and *tub2* gene regions exclusively.

### ﻿Phylogenetic analyses

To analyze their phylogenetic relationships, one or two isolates representing single genotype were selected that resulted in selection of 29 isolates representing 18 genotypes in total (Suppl. material 1). Additionally, the sequences from 90 isolates, including all ex-type isolates of all the *Calonectria* species of their respective species complexes, corresponding to 52 published *Calonectria* species, were retrieved from GenBank (Table 2). These sequences were used in phylogenetic analyses of the four individual gene regions and a combination of them.

**Table 2.** *Calonectria* spp. isolates from the published studies used for phylogenetic analyses in this study.

Species code ^a^	Species	Isolates no. ^b, c^	Other collection number ^c^	Substrate/host	Area of occurrence	Collector	GenBank accession numbers ^d^				References of source of the isolates/sequencing data
							*cmdA*	*his3*	*tef1*	*tub2*	
B1	*C.acaciicola*	CMW 47173^T^	CBS 143557	Soil (*Acaciaauriculiformis* plantation)	Do Luong, Nghe An, Vietnam	N.Q. Pham and T.Q. Pham	MT335160	MT335399	MT412690	MT412930	Pham et al. 2019; Liu et al. 2020
		CMW 47174	CBS 143558	Soil (*A.auriculiformis* plantation)	Do Luong, Nghe An, Vietnam	N.Q. Pham and T.Q. Pham	MT335161	MT335400	MT412691	MT412931	Pham et al. 2019; Liu et al. 2020
B2	*C.acicola*	CMW 30996^T^	–	*Phoenixcanariensis*	Northland, New Zealand	H. Pearson	MT335162	MT335401	MT412692	MT412932	Gadgil and Dick 2004; Lombard et al. 2010a; Liu et al. 2020
		CBS 114812	CMW 51216	*P.canariensis*	Northland, New Zealand	H. Pearson	MT335163	MT335402	MT412693	MT412933	Gadgil and Dick 2004; Lombard et al. 2010a; Liu et al. 2020
B4	*C.aconidialis*	CMW 35174^T^	CBS 136086; CERC 1850	Soil (*Eucalyptus* plantation)	Hainan, China	X. Mou and S.F. Chen	MT335165	MT335404	MT412695	OK357463	Lombard et al. 2015a; Liu et al. 2020, 2021
		CMW 35384	CBS 136091; CERC 1886	Soil (*Eucalyptus* plantation)	Hainan, China	X. Mou and S.F. Chen	MT335166	MT335405	MT412696	OK357464	Lombard et al. 2015a; Liu et al. 2020, 2021
B5	*C.aeknauliensis*	CMW 48253^T^	CBS 143559	Soil (*Eucalyptus* plantation)	Aek Nauli, North Sumatra, Indonesia	M.J. Wingfield	MT335180	MT335419	MT412710	OK357465	Pham et al. 2019; Liu et al. 2020, 2021
		CMW 48254	CBS 143560	Soil (*Eucalyptus* plantation)	Aek Nauli, North Sumatra, Indonesia	M.J. Wingfield	MT335181	MT335420	MT412711	OK357466	Pham et al. 2019; Liu et al. 2020, 2021
B8	*C.asiatica*	CBS 114073^T^	CMW 23782; CPC 3900	Debris (leaf litter)	Prathet Thai, Thailand	N.L. Hywel-Jones	AY725741	AY725658	AY725705	AY725616	Crous et al. 2004; Lombard et al. 2010a
B9	*C.auriculiformis*	CMW 47178^T^	CBS 143561	Soil (*A.auriculiformis* plantation)	Hau Loc, Thanh Hoa, Vietnam	N.Q. Pham and T.Q. Pham	MT335190	MT335430	MT412721	MT412944	Pham et al. 2019; Liu et al. 2020
		CMW 47179	CBS 143562	Soil (*A.auriculiformis* plantation)	Hau Loc, Thanh Hoa, Vietnam	N.Q. Pham and T.Q. Pham	MT335191	MT335431	MT412722	MT412945	Pham et al. 2019; Liu et al. 2020
B10	*C.australiensis*	CMW 23669^T^	CBS 112954; CPC 4714	*Ficuspleurocarpa*	Queensland, Australia	C. Pearce and B. Paulus	MT335192	MT335432	MT412723	MT412946	Crous et al. 2006; Lombard et al. 2010a; Liu et al. 2020
B14	*C.brasiliensis*	CBS 230.51^T^	IMI 299576	*Eucalyptus* sp.	Ceara state, Brazil	T.R. Ciferri	MT335200	MT335440	MT412731	MT412953	Batista 1951; Crous 2002; Lombard et al. 2010b; Liu et al. 2020
		CMW 32949	CBS 114257; CPC 1944	*Eucalyptus* sp.	Aracruz, Brazil	A.C. Alfenas	MT335201	MT335441	MT412732	MT412954	Lombard et al. 2010a; Liu et al. 2020
B17	*C.brassicicola*	CBS 112841^T^	CMW 51206; CPC 4552	Soil at *Brassica* sp.	Indonesia	M.J. Wingfield	KX784561	N/A	KX784689	KX784619	Lombard et al. 2016
B19	*C.bumicola*	CMW 48257^T^	CBS 143575	Soil (*Eucalyptus* plantation)	Aek Nauli, North Sumatra, Indonesia	M.J. Wingfield	MT335205	MT335445	MT412736	OK357467	Pham et al. 2019; Liu et al. 2020, 2021
B20	*C.canadiana*	CMW 23673^T^	CBS 110817; STE-U 499	*Picea* sp.	Canada	S. Greifenhagen	MT335206	MT335446	MT412737	MT412958	Kang et al. 2001b; Crous 2002; Lechat et al. 2010; Liu et al. 2020
		CERC 8952	–	Soil	Henan, China	S.F. Chen	MT335290	MT335530	MT412821	MT413035	Liu and Chen 2017; Liu et al. 2020
B22	*C.cerciana*	CMW 25309^T^	CBS 123693	*E.urophylla* × *E.grandis* hybrid cutting	CERC nursery, Guangdong, China	M.J. Wingfield and X.D. Zhou	MT335211	MT335451	MT412742	MT412963	Lombard et al. 2010c; Liu et al. 2020
		CMW 25290	CBS 123695	*E.urophylla* × *E.grandis* hybrid cutting	CERC nursery, Guangdong, China	M.J. Wingfield and X.D. Zhou	MT335212	MT335452	MT412743	MT412964	Lombard et al. 2010c; Liu et al. 2020
B23	*C.chinensis*	CMW 23674^T^	CBS 114827; CPC 4101	Soil	Hong Kong, China	E.C.Y. Liew	MT335220	MT335460	MT412751	MT412972	Crous et al. 2004; Lombard et al. 2010a; Liu et al. 2020
		CMW 30986	CBS 112744; CPC 4104	Soil	Hong Kong, China	E.C.Y. Liew	MT335221	MT335461	MT412752	MT412973	Crous et al. 2004; Lombard et al. 2010a; Liu et al. 2020
B26	*C.cochinchinensis*	CMW 49915^T^	CBS 143567	Soil (*Heveabrasiliensis* plantation)	Duong Minh Chau, Tay Ninh, Vietnam	N.Q. Pham, Q.N. Dang and T.Q. Pham	MT335225	MT335465	MT412756	MT412977	Pham et al. 2019; Liu et al. 2020
		CMW 47186	CBS 143568	Soil (*A.auriculiformis* plantation)	Song May, Dong Nai, Vietnam	N.Q. Pham and T.Q. Pham	MT335226	MT335466	MT412757	MT412978	Pham et al. 2019; Liu et al. 2020
B29	*C.colombiensis*	CMW 23676^T^	CBS 112220; CPC 723	Soil (*E.grandis* trees)	La Selva, Colombia	M.J. Wingfield	MT335228	MT335468	MT412759	MT412980	Crous et al. 2004; Liu et al. 2020
		CMW 30985	CBS 112221; CPC 724	Soil (*E.grandis* trees)	La Selva, Colombia	M.J. Wingfield	MT335229	MT335469	MT412760	MT412981	Crous et al. 2004; Liu et al. 2020
B30	*C.crousiana*	CMW 27249^T^	CBS 127198	*E.grandis*	Fujian, China	M.J. Wingfield	MT335230	MT335470	MT412761	MT412982	Chen et al. 2011; Liu et al. 2020
		CMW 27253	CBS 127199	*E.grandis*	Fujian, China	M.J. Wingfield	MT335231	MT335471	MT412762	MT412983	Chen et al. 2011; Liu et al. 2020
B31	*C.curvispora*	CMW 23693^T^	CBS 116159; CPC 765	Soil	Tamatave, Madagascar	P.W. Crous	MT335232	MT335472	MT412763	OK357468	Victor et al. 1997; Crous 2002; Lombard et al. 2010a, 2015a; Liu et al. 2020, 2021
		CMW 48245	CBS 143565	Soil (*Eucalyptus* plantation)	Aek Nauli, North Sumatra, Indonesia	M.J. Wingfield	MT335233	MT335473	MT412764	N/A ^e^	Pham et al. 2019; Liu et al. 2020
B32	*C.cylindrospora*	CBS 119670	CMW 51310; CPC 12766	*Pistacialentiscus*	Italy	N/A	MT335236	MT335476	MT412767	MT412985	Lombard et al. 2015a, 2015b, 2016; Liu et al. 2020
		CMW 30978	CBS 110666; P90.1479; STE-U 497	*Ilexvomitoria*	Florida, USA	N.E. El-Gholl	MT335237	MT335477	MT412768	MT412986	Crou 2002; Lombard et al. 2010a, 2015b; Liu et al. 2020
B44	*C.hawksworthii*	CBS 111870^T^	CMW 51194; CPC 2405	*Nelumbonucifera*	Pamplemousses garden, Mauritius	A. Peerally	MT335254	MT335494	MT412785	MT413003	Crous 2002; Liu et al. 2020
		CMW 31393	CBS 136641	*E.urophylla* × *E.grandis*	Guangxi, China	X. Zhou and G. Zhao	MT335247	MT335487	MT412778	MT412996	Lombard et al. 2015a; Liu et al. 2020
B46	*C.heveicola*	CMW 49913^T^	CBS 143570	Soil (*Heveabrasiliensis* plantation)	Bau Bang, Binh Duong, Vietnam	N.Q. Pham, Q.N. Dang and T.Q. Pham	MT335255	MT335495	MT412786	MT413004	Pham et al. 2019; Liu et al. 2020
		CMW 49928	CBS 143571	Soil	Bu Gia Map National Park, Binh Phuoc, Vietnam	N.Q. Pham, Q.N. Dang and T.Q. Pham	MT335280	MT335520	MT412811	MT413025	Pham et al. 2019; Liu et al. 2020
B48	*C.hongkongensis*	CBS 114828^T^	CMW 51217; CPC 4670	Soil	Hong Kong, China	M.J. Wingfield	MT335258	MT335498	MT412789	MT413007	Crous et al. 2004; Liu et al. 2020
		CERC 3570	CMW 47271	Soil (*Eucalyptus* plantation)	Beihai, Guangxi, China	S.F. Chen,J.Q. Li and G.Q. Li	MT335260	MT335500	MT412791	MT413009	Li et al. 2017; Liu et al. 2020
B51	*C.ilicicola*	CMW 30998^T^	CBS 190.50; IMI 299389; STE-U 2482	*Solanumtuberosum*	Bogor, Java, Indonesia	K.B. Boedijn and J. Reitsma	MT335266	MT335506	MT412797	OK357469	Boedijn and Reitsma 1950; Crous 2002; Lombard et al. 2010a; Liu et al. 2020, 2021
B52	*C.indonesiae*	CMW 23683^T^	CBS 112823; CPC 4508	*Syzygiumaromaticum*	Warambunga, Indonesia	M.J. Wingfield	MT335267	MT335507	MT412798	MT413015	Crous et al. 2004; Liu et al. 2020
		CBS 112840	CMW 51205; CPC 4554	*S.aromaticum*	Warambunga, Indonesia	M.J. Wingfield	MT335268	MT335508	MT412799	MT413016	Crous et al. 2004; Liu et al. 2020
B54	*C.insularis*	CMW 30991^T^	CBS 114558; CPC 768	Soil	Tamatave, Madagascar	P.W. Crous	MT335269	MT335509	MT412800	MT413017	Schoch et al. 1999; Lombard et al. 2010a, 2016; Liu et al. 2020
		CMW 30992	CBS 114559; CPC 954	Soil	Conejos, Veracruz, Mexico	M.J. Wingfield	MT335270	MT335510	MT412801	MT413018	Lombard et al. 2010a, 2016; Liu et al. 2020
B55	*C.kyotensis*	CBS 114525^T^	ATCC 18834; CMW 51824; CPC 2367	*Robiniapseudoacacia*	Japan	T. Terashita	MT335271	MT335511	MT412802	MT413019	Terashita 1968; Crous 2002; Lombard et al. 2016; Liu et al. 2020
		CBS 114550	CMW 51825; CPC 2351	Soil	China	M.J. Wingfield	MT335246	MT335486	MT412777	MT412995	Lombard et al. 2016; Liu et al. 2020
B56	*C.lageniformis*	CBS 111324^T^	CMW 51177; CPC 1473	Leaf of *Eucalyptus* sp.	Rivière Noire, Mauritius	H. Smith	KX784574	N/A	KX784702	KX784632	Lombard et al. 2016; Marin-Felix et al. 2017
B57	*C.lantauensis*	CERC 3302^T^	CBS 142888; CMW 47252	Soil	Lidao, Hong Kong, China	M.J. Wingfield and S.F. Chen	MT335272	MT335512	MT412803	OK357470	Li et al. 2017; Liu et al. 2020, 2021
		CERC 3301	CBS 142887; CMW 47251	Soil	Lidao, Hong Kong, China	M.J. Wingfield and S.F. Chen	MT335273	MT335513	MT412804	OK357471	Li et al. 2017; Liu et al. 2020, 2021
B58	*C.lateralis*	CMW 31412^T^	CBS 136629	Soil (*Eucalyptus* plantation)	Guangxi, China	X. Zhou, G. Zhao and F. Han	MT335274	MT335514	MT412805	MT413020	Lombard et al. 2015a; Liu et al. 2020
B63	*C.lombardiana*	CMW 30602^T^	CBS 112634; CPC 4233; Lynfield 417	*Xanthorrhoeaaustralis*	Victoria, Australia	T. Baigent	MT335395	MT335635	MT412926	MT413133	Crous 2002; Crous et al. 2006; Lombard et al. 2010c; Liu et al. 2020
B66	*C.malesiana*	CMW 23687^T^	CBS 112752; CPC 4223	Soil	Northern Sumatra, Indonesia	M.J. Wingfield	MT335286	MT335526	MT412817	MT413031	Crous et al. 2004; Liu et al. 2020
		CBS 112710	CMW 51199; CPC 3899	Leaf litter	Prathet, Thailand	N.L. Hywel-Jones	MT335287	MT335527	MT412818	MT413032	Crous et al. 2004; Liu et al. 2020
B67	*C.maranhensis*	CBS 134811^T^	LPF142	*Eucalyptus* sp. (leaf)	Açailandia, Maranhao, Brazil	A.C. Alfenas	KM396035	KM396118	KM395861	KM395948	Alfenas et al. 2015
		CBS 134812	LPF143	*Eucalyptus* sp. (leaf)	Açailandia, Maranhao, Brazil	A.C. Alfenas	KM396036	KM396119	KM395862	KM395949	Alfenas et al. 2015
B74	*C.multiseptata*	CMW 23692^T^	CBS 112682; CPC 1589	*E.grandis*	North Sumatra, Indonesia	M.J. Wingfield	MT335299	MT335539	MT412830	MT413044	Crous et al. 2004; Lombard et al. 2010a; Liu et al. 2020
B80	*C.pacifica*	CMW 16726^T^	A1568; CBS 109063; IMI 354528; STE-U 2534	*Araucariaheterophylla*	Hawaii, USA	M. Aragaki	MT335311	MT335551	MT412842	OK357472	Kang et al. 2001b; Crous 2002, Crous et al. 2004; Liu et al. 2020, 2021
		CMW 30988	CBS 114038	*Ipomoeaaquatica*	Auckland, New Zealand	C.F. Hill	MT335312	MT335552	MT412843	OK357473	Crous 2002; Crous et al. 2004; Lombard et al. 2010a; Liu et al. 2020, 2021
B86	*C.penicilloides*	CMW 23696^T^	CBS 174.55; STE-U 2388	*Prunus* sp.	Hatizyo Island, Japan	M. Ookubu	MT335338	MT335578	MT412869	MT413081	Tubaki 1958; Crous 2002; Liu et al. 2020
B89	*C.plurilateralis*	CBS 111401^T^	CMW 51178; CPC 1637	Soil	Ecuador	M.J. Wingfield	MT335340	MT335580	MT412871	MT413083	Lombard et al. 2016; Liu et al. 2020
B90	*C.propaginicola*	CBS 134815^T^	LPF220	*Eucalyptus* sp. (seeding)	Santana, Pará, Brazil	A.C. Alfenas	KM396040	KM396123	KM395866	KM395953	Alfenas et al. 2015
		CBS 134816	LPF222	*Eucalyptus* sp. (seeding)	Santana, Pará, Brazil	A.C. Alfenas	KM396041	KM396124	KM395867	KM395954	Alfenas et al. 2015
B97	*C.pseudoreteaudii*	CMW 25310^T^	CBS 123694	*E.urophylla* × *E.grandis*	Guangdong, China	M.J. Wingfield and X.D. Zhou	MT335354	MT335594	MT412885	MT413096	Lombard et al. 2010c; Liu et al. 2020
		CMW 25292	CBS 123696	*E.urophylla* × *E.grandis*	Guangdong, China	M.J. Wingfield and X.D. Zhou	MT335355	MT335595	MT412886	MT413097	Lombard et al. 2010c; Liu et al. 2020
B104	*C.queenslandica*	CMW 30604^T^	CBS 112146; CPC 3213	*E.urophylla*	Lannercost, Queensland, Australia	B. Brown	MT335367	MT335607	MT412898	MT413108	Kang et al. 2001a; Lombard et al. 2010c; Liu et al. 2020
		CMW 30603	CBS 112155; CPC 3210	*E.pellita*	Lannercost, Queensland, Australia	P.Q Thu and K.M. Old	MT335368	MT335608	MT412899	MT413109	Kang et al. 2001a; Lombard et al. 2010c; Liu et al. 2020
B106	*C.reteaudii*	CMW 30984^T^	CBS 112144; CPC 3201	*E.camaldulensis*	Chon Thanh, Binh Phuoc, Vietnam	M.J. Dudzinski and P.Q. Thu	MT335370	MT335610	MT412901	MT413111	Kang et al. 2001a; Crous 2002; Crous et al. 2006; Liu et al. 2020
		CMW 16738	CBS 112143; CPC 3200	*Eucalyptus* leaves	Binh Phuoc, Vietnam	M.J. Dudzinski and P.Q. Thu	MT335371	MT335611	MT412902	MT413112	Kang et al. 2001a; Crous 2002; Crous et al. 2006; Liu et al. 2020
B112	*C.sumatrensis*	CMW 23698^T^	CBS 112829; CPC 4518	Soil	Northern Sumatra, Indonesia	M.J. Wingfield	MT335382	MT335622	MT412913	OK357474	Crous et al. 2004; Liu et al. 2020, 2021
		CMW 30987	CBS 112934; CPC 4516	Soil	Northern Sumatra, Indonesia	M.J. Wingfield	MT335383	MT335623	MT412914	OK357475	Crous et al. 2004; Liu et al. 2020, 2021
B113	*C.syzygiicola*	CBS 112831^T^	CMW 51204; CPC 4511	*Syzygiumaromaticum*	Sumatra, Indonesia	M.J. Wingfield	N/A	N/A	KX784736	KX784663	Lombard et al. 2016
B115	*C.tonkinensis*	CMW 47430^T^	CBS 143576	Soil (*Eucalyptus* plantation)	Bavi, Hanoi, Vietnam	N.Q. Pham and T.Q. Pham	MT335384	MT335624	MT412915	MT413122	Pham et al. 2019; Liu et al. 2020
B116	*C.uniseptata*	CBS 413.67^T^	CMW 23678; CPC 2391; IMI 299577	*Paphiopedilumcallosum*	Celle, Germany	W. Gerlach	GQ267379	GQ267248	GQ267307	GQ267208	Lombard et al. 2016
B118	*C.variabilis*	CMW 3187^T^	AR2675; CBS 114677; CPC 2436	*Scheffleramorototoni*	Pará, Brazil	F.C. de Albuquerque	MT335392	MT335632	MT412923	MT413130	Crous et al. 1993; Crous 2002; Lombard et al. 2010a, 2016; Liu et al. 2020
		CMW 2914	CBS 112691; CPC 2506	*Theobromagrandiflorum*	Pará, Brazil	F. Carneiro	MT335393	MT335633	MT412924	MT413131	Crous et al. 1993; Crous 2002; Lombard et al. 2010a, 2016; Liu et al. 2020
B120	*C.yunnanensis*	CERC 5339^T^	CBS 142897; CMW 47644	Soil (*Eucalyptus* plantation)	Yunnan, China	S.F. Chen and J.Q. Li	MT335396	MT335636	MT412927	MT413134	Li et al. 2017; Liu et al. 2020
		CERC 5337	CBS 142895; CMW 47642	Soil (*Eucalyptus* plantation)	Yunnan, China	S.F. Chen and J.Q. Li	MT335397	MT335637	MT412928	MT413135	Li et al. 2017; Liu et al. 2020
B124	*C.singaporensis*	CBS 146715^T^	MUCL 048320	leaf litter (submerged in a small stream)	South East Asian rainforest, Mac Ritchie Reservoir, Singapore	C. Decock	MW890042	MW890055	MW890086	MW890124	Crous et al. 2021
		CBS 146713	MUCL 048171	leaf litter (submerged in a small stream)	South East Asian rainforest, Mac Ritchie Reservoir, Singapore	C. Decock	MW890040	MW890053	MW890084	MW890123	Crous et al. 2021
B127	*C.borneana*	CMW 50782^T^	CBS 144553	Soil (*Eucalyptus* plantation)	Sabah, Tawau, Brumas, Malaysia	N.Q. Pham, Marincowitz and M.J. Wingfield	OL635067	OL635043	OL635019	N/A	Pham et al. 2022a
		CMW 50832	CBS 144551	Soil (*Eucalyptus* plantation)	Sabah, Tawau, Brumas, Malaysia	N.Q. Pham, Marincowitz and M.J. Wingfield	OL635065	OL635041	OL635017	N/A	Pham et al. 2022a
B128	*C.ladang*	CMW 50776^T^	CBS 144550	Soil (*Eucalyptus* plantation)	Sabah, Tawau, Brumas, Malaysia	N.Q. Pham, Marincowitz and M.J. Wingfield	OL635075	OL635051	OL635027	N/A	Pham et al. 2022a
		CMW 50775	CBS 144549	Soil (*Eucalyptus* plantation)	Sabah, Tawau, Brumas, Malaysia	N.Q. Pham, Marincowitz and M.J. Wingfield	OL635074	OL635050	OL635026	N/A	Pham et al. 2022a
B129	*C.pseudomalesiana*	CMW 50821^T^	CBS 144563	Soil (*Eucalyptus* plantation)	Sabah, Tawau, Brumas, Malaysia	N.Q. Pham, Marincowitz and M.J. Wingfield	OL635076	OL635052	OL635028	OL635137	Pham et al. 2022a
		CMW 50779	CBS 144668	Soil (*Eucalyptus* plantation)	Sabah, Tawau, Brumas, Malaysia	N.Q. Pham, Marincowitz and M.J. Wingfield	OL635077	OL635053	OL635029	OL635138	Pham et al. 2022a
B130	*C.tanah*	CMW 50777^T^	CBS 144562	Soil (*Eucalyptus* plantation)	Sabah, Tawau, Brumas, Malaysia	N.Q. Pham, Marincowitz and M.J. Wingfield	OL635088	OL635064	OL635040	OL635146	Pham et al. 2022a
		CMW 50771	CBS 144560	Soil (*Eucalyptus* plantation)	Sabah, Tawau, Brumas, Malaysia	N.Q. Pham, Marincowitz and M.J. Wingfield	OL635086	OL635062	OL635038	OL635144	Pham et al. 2022a
B131	*C.cassiae*	ZHKUCC 210011 T	–	*Cassiasurattensis*	Guangzhou City, Guangdong, China	Y. X. Zhang, C. T. Chen, Manawas., and M. M. Xiang	ON260790	N/A	MZ516860	MZ516863	Zhang et al. 2022
		ZHKUCC 210012	–	*Cassiasurattensis*	Guangzhou City, Guangdong, China	Y. X. Zhang, C. T. Chen, Manawas., and M. M. Xiang	ON260791	N/A	MZ516861	MZ516864	Zhang et al. 2022
B132	*C.guangdongensis*	ZHKUCC 21-0062T	–	*Heliconiametallica*	Guangdong, China	Y. X. Zhang, C. T. Chen, Manawas., and M. M. Xiang	MZ491127	N/A	MZ491149	MZ491171	Zhang et al. 2022
		ZHKUCC 21-0063	–	*Heliconiametallica*	Guangdong, China	Y. X. Zhang, C. T. Chen, Manawas., and M. M. Xiang	MZ491128	N/A	MZ491150	MZ491172	Zhang et al. 2022
	*Curvicladiellacignea*	CBS 109167T	CPC 1595; MUCL 40269	Decaying leaf	French Guiana	C. Decock	KM231287	KM231461	KM231867	KM232002	Decock and Crous 1998; Crous et al. 2006; Lombard et al. 2015b
		CBS 109168	CPC 1594; MUCL 40268	Decaying seed	French Guiana	C. Decock	KM231286	KM231460	KM231868	KM232003	Decock and Crous 1998; Crous et al. 2006; Lombard et al. 2015b

^a^ Codes (B1–B120) of the 120 accepted *Calonectria* species accepted according to Liu et al. (2020). ^b^ T: ex-type isolates of the species. ^c^ AR: Amy Y. Rossman working collection; ATCC: American Type Culture Collection, Virginia, USA; CBS: Westerdijk Fungal Biodiversity Institute, Utrecht, The Netherlands; CERC: China Eucalypt Research Centre, Zhanjiang, Guangdong Province, China; CMW: Culture collection of the Forestry and Agricultural Biotechnology Institute (FABI), University of Pretoria, Pretoria, South Africa; CPC: Pedro Crous working collection housed at Westerdijk Fungal Biodiversity Institute; IMI: International Mycological Institute, CABI Bioscience, Egham, Bakeham Lane, UK; MUCL: Mycotheque, Laboratoire de Mycologie Systematique st Appliqee, I’Universite, Louvian-la-Neuve, Belgium; STE-U: Department of Plant Pathology, University of Stellenbosch, South Africa; ZHKUCC: Zhongkai University of Agriculture and Engineering Culture Collection; –: no other collection number. ^d^*tef1*: translation elongation factor 1-alpha; *tub2*: β-tubulin; *cmdA*: calmodulin; *his3*: histone H3. ^e^ N/A: information is not available.

**Figure 3.**
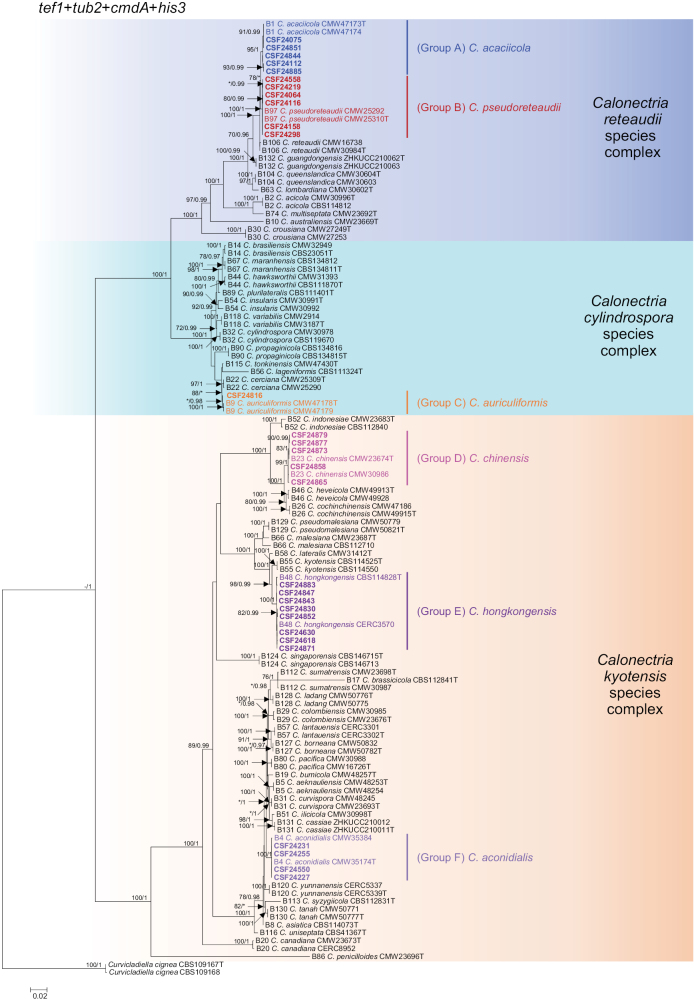
Phylogenetic tree of *Calonectria* species based on maximum likelihood (ML) analyses of a combined DNA dataset of *tef1*, *tub2*, *cmdA*, and *his3* gene sequences. Bootstrap support values ≥ 70% for ML and posterior probability values ≥ 0.95 for Bayesian inference (BI) analyses are presented above the branches as ML/BI. Bootstrap values < 70% or probability values < 0.95 are marked with “*,” and absent analysis values are marked with “-”. Ex-type isolates are marked with “T.” Isolates sequenced in this study are highlighted in bold. The outgroup taxon was *Curvicladiellacignea* (CBS 109167 and CBS 109168).

The sequenced isolates yielded approximately 500 bp for *tef1*, 560 bp for *tub2*, 680 bp for *cmdA*, and 430 bp for *his3* gene regions. The model for each gene region was selected based on jModeltest v. 2.1.5. The TIM2+I+G, HKY+I+G, TrN+I+G, HKY+I+G, and TIM2+I+G models were selected for *tef1*, *tub2*, *cmdA*, *his3*, and the consolidated dataset, respectively. The topological structures generated from BI analyses were similar to those generated from ML analyses for each dataset. The ML trees displayed bootstrap values from ML and the posterior probabilities from BI are shown in Fig. 3, Suppl. materials 2–5.

The 29 *Calonectria* isolates were clustered into six distinct groups (Groups A–F) based on the phylogenetic analyses of the four gene regions’ combination (Fig. 3). Among them, isolates in Group A and Group B belong to the *C.reteaudii* complex. Isolates in Group A were clustered with or closely related to *C.acaciicola*, *C.pseudoreteaudii*, *C.reteaudii*, or *C.guangdongensis* in the *tef1*, *cmdA*, and *his3* trees (Suppl. materials 2, 4, 5), and with *C.acaciicola* in the *tub2* tree (Suppl. material 3). The *tef1*/*tub2*/*cmdA*/*his3* tree confirmed that isolates in Group A were most closely related to *C.acaciicola* (Fig. 3), and thus they were accepted as belonging to this species. Isolates in Group B were clustered with, or most closely related to, *C.pseudoreteaudii* in each of the *tef1*, *tub2*, *cmdA*, *his3*, and *tef1*/*tub2*/*cmdA*/*his3* trees (Fig. 3, Suppl. materials 2–5). Thus, isolates in Group B are referred as *C.pseudoreteaudii*.

Isolate CSF24816 (Group C) was grouped in the *C.cylindrospora* species complex (Fig. 3, Suppl. materials 2–5). It was clustered with *C.auriculiformis* in the *tef1* tree, with *C.cerciana* in the *tub2* tree, with *C.cerciana* and *C.tonkinensis* in the *cmdA* tree, and with *C.auriculiformis*, *C.cerciana*, and *C.tonkinensis* in the *his3* tree (Suppl. materials 2–5). It was most closely related to *C.auriculiformis* in the *tef1/tub2/cmdA/his3* tree (Fig. 3), thus the isolate CSF24816 was identified as *C.auriculiformis*.

Isolates in Groups D, E, and F resided in the *C.kyotensis* species complex based on the phylogenetic trees of *tef1*, *tub2*, *cmdA*, *his3*, and *tef1/tub2/cmdA/his3* (Fig. 3, Suppl. materials 2–5). Isolates in Group D, Group E, and Group F were consistently clustered with or most closely related to *C.chinensis*, *C.hongkongensis*, and *C.aconidialis*, respectively (Fig. 3, Suppl. materials 2–5). Therefore, isolates in Group D, Group E, and Group F were identified as *C.chinensis*, *C.hongkongensis* and *C.aconidialis*, respectively.

### ﻿*Calonectria* distribution associated with diseased leaves and soil in *Eucalyptus* plantations

**Figure 4.**
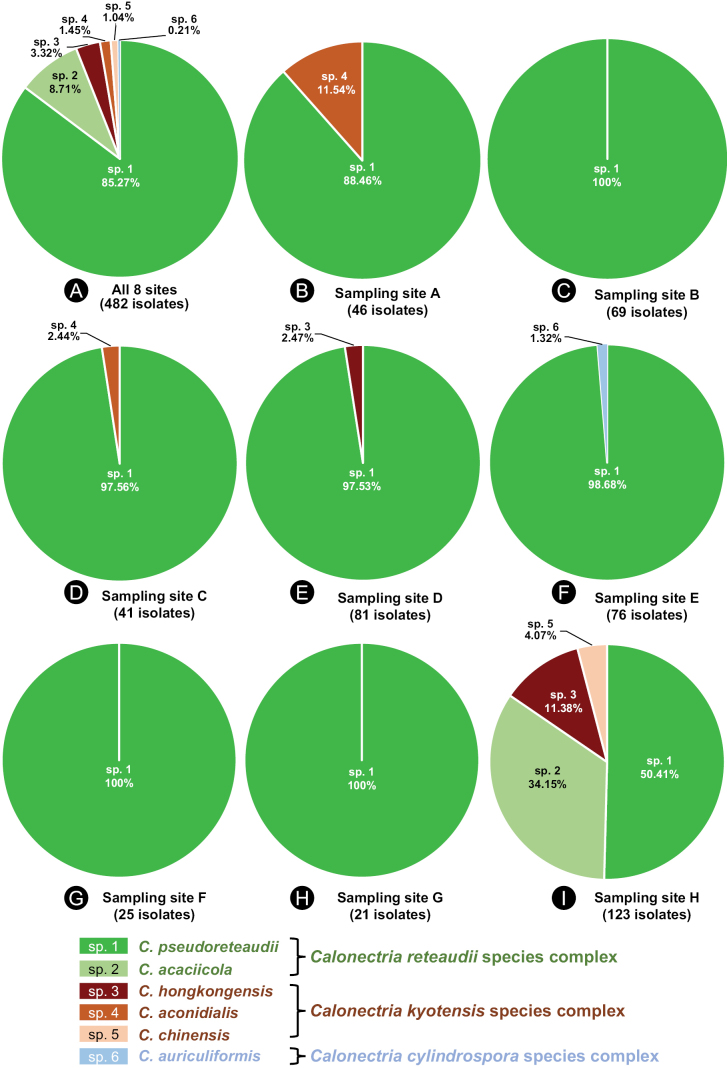
The isolate number and percentage of each *Calonectria* species at the eight sampling sites. “sp. 1, 2, 3, 4, 5, 6” indicate the six *Calonectria* species **A** isolates and species obtained from all eight sites **B-I** isolates and species obtained from a particular site (sites **A–H**).

The 482 *Calonectria* isolates used for molecular identification in the current study were identified as six species, which resided in three species complexes. The six species were *C.pseudoreteaudii* (411 isolates, 85.27%), *C.acaciicola* (42 isolates, 8.71%), *C.hongkongensis* (16 isolates, 3.32%), *C.aconidialis* (seven isolates, 1.45%), *C.chinensis* (five isolates, 1.04%), and *C.auriculiformis* (one isolate, 0.21%) (Fig. 4).

**Figure 5.**
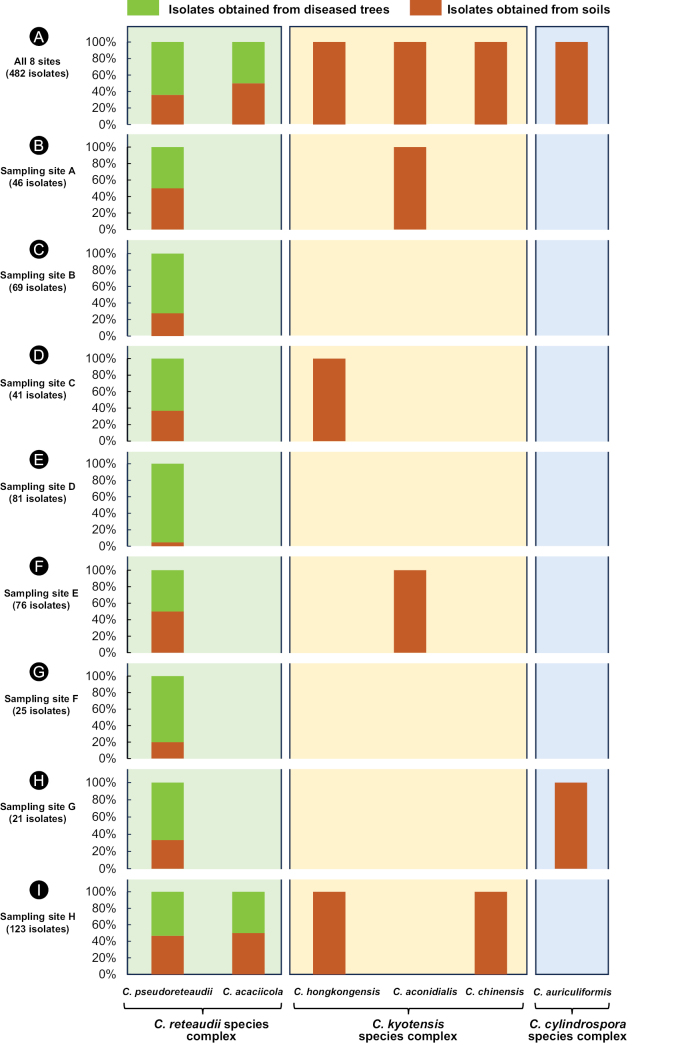
Histogram showing the proportions of each of the six *Calonectria* species reside in three species complexes isolated from diseased leaves and soil at the eight sampling sites. The histograms in green and orange indicated isolates obtained from diseased trees and soils, respectively **A** species obtained from all the eight sites **B–I** species obtained from a particular site (sites **A–H**).

At each of the eight sampling sites, *C.pseudoreteaudii* was dominant in the samples collected from both the diseased trees and soil under these trees, particularly at sites A–G located on the mainland of China. *Calonectriaacaciicola* isolates were obtained from site H in Hainan Province, and this species was also frequently isolated from both diseased trees and soil. The other four *Calonectria* species, *C.hongkongensis*, *C.aconidialis*, *C.chinensis*, and *C.auriculiformis*, were only isolated from samples collected from soils (Table 1, Figs 4, 5).

When considering the species complexes associated with diseased leaves and soils, all *Calonectria* isolates obtained from diseased trees resided in the *C.reteaudii* species complex; and 14.14% of *Calonectria* isolates obtained from soils resided in the *C.kyotensis* species complex. All isolates residing in the *C.kyotensis* species complex were obtained from soils. For the isolates residing in the *C.reteaudii* species complex, 62.69% of the isolates come from diseased trees and 37.31% were from soil samples (Table 1, Fig. 5).

## ﻿Discussion

In this study, a systematic and comprehensive investigation of Calonectria leaf blight occurring on *Eucalyptus* plantations in a wide geographic range in southern China was conducted. The results of this study clearly showed that the *Calonectria* species in the *C.cylindrospora* species complex was occasionally distributed in *Eucalyptus* plantations. *Calonectria* species in both the *C.reteaudii* species complex and *C.kyotensis* species complex were widely distributed. The distribution patterns of *Calonectria* species in the *C.reteaudii* species complex and *C.kyotensis* species complex were related to diseased leaves and soil habitats during leaf blight outbreak season in *Eucalyptus* plantations in southern China.

The results of this study showed that all isolates obtained from diseased trees resided in the *C.reteaudii* species complex, which indicated that they are the causal agents of Calonectria leaf blight at the sampled sites in China. Moreover, *C.pseudoreteaudii* was the dominant species of all the eight sampling sites in the three provinces. This was consistent with previous studies in which *C.pseudoreteaudii* was frequently obtained from diseased *Eucalyptus* trees in Guangdong, Guangxi, Fujian, and Hainan Provinces in southern China (Chen et al. 2013; Wang and Chen 2020; Wu and Chen 2021; Li et al. 2023a; Liang et al. 2023). *Calonectriaacaciicola* was only isolated from site H in Hainan Province in this and a previous study by Liang et al. (2023). Furthermore, inoculation results from previous studies indicated that both *C.pseudoreteaudii* and *C.acaciicola* were highly virulent to the tested *Eucalyptus* genotypes (Liang et al. 2023). *C.pseudoreteaudii* is one of the main causal agents of Calonectria leaf blight widely observed in southern China, and *C.acaciicola* is causal agent of the disease in Hainan Province in particular.

It is still unclear whether the species in the *C.reteaudii* complex are soil-borne or not. The results of the previous studies consistently indicated that species in the *C.reteaudii* complex can survive in the soils, at least for a certain time (Crous 2002; Liu et al. 2021). Both *C.pseudoreteaudii* and *C.acaciicola* were frequently isolated from soils under the diseased trees in this study. Results of the previous research confirmed that *Calonectria* species in the *C.reteaudii* species complex were frequently isolated from soils under diseased *Eucalyptus* trees in southern China (Wu and Chen 2021; Li et al. 2023b). A recent population study showed that the genetic diversity of the *C.pseudoreteaudii* isolates obtained from diseased leaves was higher than that of the *C.pseudoreteaudii* isolates obtained from the soil in one *Eucalyptus* plantation, and the *C.pseudoreteaudii* isolates in soil may spread from diseased leaves (Wu et al. 2023). The results of the current study highlight that *C.pseudoreteaudii* from the soils in *Eucalyptus* plantations also needs to be carefully monitored for disease management purposes. It is necessary to clarify whether *Calonectria* in the *C.reteaudii* species complex is soil-borne or not and further understand the sources and dispersal pathways of the pathogens from this complex.

Previous studies showed that the species in the *C.kyotensis* complex were widely isolated from soils, both in natural forests and commercial plantations (Liu et al. 2021, 2022; Wu and Chen 2021, 2023; Li et al. 2023b; Liu and Chen 2023), and a relatively small number of isolates were isolated from susceptible *Eucalyptus* leaves (Li et al. 2023a; Liang et al. 2023). The research in this study indicated that all isolates residing in the *C.kyotensis* species complex were obtained from soils but not from diseased trees. Moreover, the results of this study revealed that isolates in the *C.kyotensis* species complex may not be the pathogens causing leaf blight in *Eucalyptus*. Further research is needed to clarify their ecological niche since they were also frequently isolated from diseased leaves (Li et al. 2023b; Liang et al. 2023).

## ﻿Conclusion

This study clarified the distribution patterns of *Calonectria* species complexes related to the *Calonectria* isolated sources of diseased trees and soils during the disease outbreak season. The results of this study clearly showed that all isolates obtained from diseased leaves resided in the *C.reteaudii* species complex, and species in the *C.reteaudii* species complex were widely distributed in diseased leaves and soils. All the isolates residing in the *C.kyotensis* species complex were obtained from soils. This indicated that species in the *C.kyotensis* species complex are soil inhabitants. These results highlight that *Calonectria* species in the *C.reteaudii* species complex, but not in the *C.kyotensis* species complex, are the causal agents of Calonectria leaf blight in southern China. More attention should be paid to the causal agents of Calonectria leaf blight, especially *C.pseudoreteaudii*, with a wide geographic distribution during the disease outbreak season, for disease management in the future.

## ﻿Additional information

### Author ORCIDs

WenXia Wu https://orcid.org/0009-0000-1685-9627

ShuaiFei Chen https://orcid.org/0000-0002-3920-9982

### Data availability

All of the data that support the findings of this study are available in the main text or Supplementary Information.

## Supplementary materials

Supplementary material 1 *Calonectria* isolates obtained from eight *Eucalyptus* plantations in this study Data type docx https://binary.pensoft.net/file/1167650This dataset is made available under the Open Database License (http://opendatacommons.org/licenses/odbl/1.0/). The Open Database License (ODbL) is a license agreement intended to allow users to freely share, modify, and use this Dataset while maintaining this same freedom for others, provided that the original source and author(s) are credited.WenXia Wu, ShuaiFei Chen

Supplementary material 2 Phylogenetic tree of *Calonectria* species based on maximum likelihood (ML) analyses of a combined DNA dataset of *tef1* gene sequences Data type pdf https://binary.pensoft.net/file/1167651This dataset is made available under the Open Database License (http://opendatacommons.org/licenses/odbl/1.0/). The Open Database License (ODbL) is a license agreement intended to allow users to freely share, modify, and use this Dataset while maintaining this same freedom for others, provided that the original source and author(s) are credited.WenXia Wu, ShuaiFei Chen

Supplementary material 3 Phylogenetic tree of *Calonectria* species based on maximum likelihood (ML) analyses of a combined DNA dataset of *tub2* gene sequences Data type pdf https://binary.pensoft.net/file/1167652This dataset is made available under the Open Database License (http://opendatacommons.org/licenses/odbl/1.0/). The Open Database License (ODbL) is a license agreement intended to allow users to freely share, modify, and use this Dataset while maintaining this same freedom for others, provided that the original source and author(s) are credited.WenXia Wu, ShuaiFei Chen

Supplementary material 4 Phylogenetic tree of *Calonectria* species based on maximum likelihood (ML) analyses of a combined DNA dataset of *cmdA* gene sequences Data type pdf https://binary.pensoft.net/file/1167653This dataset is made available under the Open Database License (http://opendatacommons.org/licenses/odbl/1.0/). The Open Database License (ODbL) is a license agreement intended to allow users to freely share, modify, and use this Dataset while maintaining this same freedom for others, provided that the original source and author(s) are credited.WenXia Wu, ShuaiFei Chen

Supplementary material 5 Phylogenetic tree of *Calonectria* species based on maximum likelihood (ML) analyses of a combined DNA dataset of *his3* gene sequences Data type pdf https://binary.pensoft.net/file/1167654This dataset is made available under the Open Database License (http://opendatacommons.org/licenses/odbl/1.0/). The Open Database License (ODbL) is a license agreement intended to allow users to freely share, modify, and use this Dataset while maintaining this same freedom for others, provided that the original source and author(s) are credited.WenXia Wu, ShuaiFei Chen
