# Supplementary material for: ﻿Distribution patterns of Calonectria (Ascomycota, Sordariomycetes, Hypocreales, Nectriaceae) species complexes related to diseased leaves and soil habitats during leaf blight outbreak season in Eucalyptus plantations in southern China
Source: MycoKeys. 2024 Nov 4;110:117–40. doi: 10.3897/mycokeys.110.130733 (PMC11555429; doi:10.3897/mycokeys.110.130733)
Supplement: Supplementary material 1 — Calonectria isolates obtained from eight Eucalyptus plantations in this study [file mycokeys-110-117-s001.docx]

**Supplementary Table S1.** *Calonectria* spp. isolates obtained from eight *Eucalyptus* plantations in this study.

| **Species** | **Isolate no. ^a, b^** | **Genotype ^c^** | **Substrate** | **Site code** | **Coordinates** | **Collectors** | **GenBank accession no. ^d^** | | | |  |  |
| --- | --- | --- | --- | --- | --- | --- | --- | --- | --- | --- | --- | --- |
|  |  |  |  |  |  |  | ***tef1*** | ***tub2*** | ***cmdA*** | ***his3*** |  |  |
| *Calonectria reteaudii* species complex | | | | | | | | | | |  |  |
| *C. acaciicola* | **CSF24074** | AA-- ^f^ | 1-year-old *E. urophylla × E. grandis* leaf | H | 19º47'41.46"N, 109º49'52.71"E | S. F. Chen, Q. C. Wang, X. Y. Liang and L. F. Liu | *OQ187861* | *OQ210221* | N/A ^g^ | N/A |  |  |
| *C. acaciicola* | **CSF24075 ^e^** | AAAA | 1-year-old *E. urophylla × E. grandis* leaf | H | 19º47'41.46"N, 109º49'52.71"E | S. F. Chen, Q. C. Wang, X. Y. Liang and L. F. Liu | *OQ187826* | *OQ210186* | *OQ210585* | *OQ230656* |  |  |
| *C. acaciicola* | **CSF24076** | AA-- | 1-year-old *E. urophylla × E. grandis* leaf | H | 19º47'41.46"N, 109º49'52.71"E | S. F. Chen, Q. C. Wang, X. Y. Liang and L. F. Liu | *OQ187862* | *OQ210222* | N/A | N/A |  |  |
| *C. acaciicola* | **CSF24078** | AA-- | 1-year-old *E. urophylla × E. grandis* leaf | H | 19º47'41.46"N, 109º49'52.71"E | S. F. Chen, Q. C. Wang, X. Y. Liang and L. F. Liu | *OQ187863* | *OQ210223* | N/A | N/A |  |  |
| *C. acaciicola* | **CSF24080** | AA-- | 1-year-old *E. urophylla × E. grandis* leaf | H | 19º47'41.46"N, 109º49'52.71"E | S. F. Chen, Q. C. Wang, X. Y. Liang and L. F. Liu | *OQ187864* | *OQ210224* | N/A | N/A |  |  |
| *C. acaciicola* | **CSF24081** | AA-- | 1-year-old *E. urophylla × E. grandis* leaf | H | 19º47'41.46"N, 109º49'52.71"E | S. F. Chen, Q. C. Wang, X. Y. Liang and L. F. Liu | *OQ187865* | *OQ210225* | N/A | N/A |  |  |
| *C. acaciicola* | **CSF24082** | AAAA | 1-year-old *E. urophylla × E. grandis* leaf | H | 19º47'41.46"N, 109º49'52.71"E | S. F. Chen, Q. C. Wang, X. Y. Liang and L. F. Liu | *OQ187827* | *OQ210187* | *OQ210586* | *OQ230657* |  |  |
| *C. acaciicola* | **CSF24083** | AA-- | 1-year-old *E. urophylla × E. grandis* leaf | H | 19º47'41.46"N, 109º49'52.71"E | S. F. Chen, Q. C. Wang, X. Y. Liang and L. F. Liu | *OQ187866* | *OQ210226* | N/A | N/A |  |  |
| *C. acaciicola* | **CSF24088** | AA-- | 1-year-old *E. urophylla × E. grandis* leaf | H | 19º47'41.46"N, 109º49'52.71"E | S. F. Chen, Q. C. Wang, X. Y. Liang and L. F. Liu | *OQ187867* | *OQ210227* | N/A | N/A |  |  |
| *C. acaciicola* | **CSF24089** | AA-- | 1-year-old *E. urophylla × E. grandis* leaf | H | 19º47'41.46"N, 109º49'52.71"E | S. F. Chen, Q. C. Wang, X. Y. Liang and L. F. Liu | *OQ187868* | *OQ210228* | N/A | N/A |  |  |
| *C. acaciicola* | **CSF24090** | AA-- | 1-year-old *E. urophylla × E. grandis* leaf | H | 19º47'41.46"N, 109º49'52.71"E | S. F. Chen, Q. C. Wang, X. Y. Liang and L. F. Liu | *OQ187869* | *OQ210229* | N/A | N/A |  |  |
| *C. acaciicola* | **CSF24091** | AA-- | 1-year-old *E. urophylla × E. grandis* leaf | H | 19º47'41.46"N, 109º49'52.71"E | S. F. Chen, Q. C. Wang, X. Y. Liang and L. F. Liu | *OQ187870* | *OQ210230* | N/A | N/A |  |  |
| *C. acaciicola* | **CSF24097** | AA-- | 1-year-old *E. urophylla × E. grandis* leaf | H | 19º47'41.46"N, 109º49'52.71"E | S. F. Chen, Q. C. Wang, X. Y. Liang and L. F. Liu | *OQ187871* | *OQ210231* | N/A | N/A |  |  |
| *C. acaciicola* | **CSF24098** | AAAA | 1-year-old *E. urophylla × E. grandis* leaf | H | 19º47'41.46"N, 109º49'52.71"E | S. F. Chen, Q. C. Wang, X. Y. Liang and L. F. Liu | *OQ187828* | *OQ210188* | *OQ210587* | *OQ230658* |  |  |
| *C. acaciicola* | **CSF24099** | AA-- | 1-year-old *E. urophylla × E. grandis* leaf | H | 19º47'41.46"N, 109º49'52.71"E | S. F. Chen, Q. C. Wang, X. Y. Liang and L. F. Liu | *OQ187872* | *OQ210232* | N/A | N/A |  |  |
| *C. acaciicola* | **CSF24100** | AA-- | 1-year-old *E. urophylla × E. grandis* leaf | H | 19º47'41.46"N, 109º49'52.71"E | S. F. Chen, Q. C. Wang, X. Y. Liang and L. F. Liu | *OQ187873* | *OQ210233* | N/A | N/A |  |  |
| *C. acaciicola* | **CSF24102** | AA-- | 1-year-old *E. urophylla × E. grandis* leaf | H | 19º47'41.46"N, 109º49'52.71"E | S. F. Chen, Q. C. Wang, X. Y. Liang and L. F. Liu | *OQ187874* | *OQ210234* | N/A | N/A |  |  |
| *C. acaciicola* | **CSF24112 ^e^** | BABB | 1-year-old *E. urophylla × E. grandis* leaf | H | 19º47'41.46"N, 109º49'52.71"E | S. F. Chen, Q. C. Wang, X. Y. Liang and L. F. Liu | *OQ188011* | *OQ210371* | *OQ210634* | *OQ230705* |  |  |
| *C. acaciicola* | **CSF24113** | BABB | 1-year-old *E. urophylla × E. grandis* leaf | H | 19º47'41.46"N, 109º49'52.71"E | S. F. Chen, Q. C. Wang, X. Y. Liang and L. F. Liu | *OQ188012* | *OQ210372* | *OQ210635* | *OQ230706* |  |  |
| *C. acaciicola* | **CSF24115** | BABB | 1-year-old *E. urophylla × E. grandis* leaf | H | 19º47'41.46"N, 109º49'52.71"E | S. F. Chen, Q. C. Wang, X. Y. Liang and L. F. Liu | *OQ188013* | *OQ210373* | *OQ210636* | *OQ230707* |  |  |
| *C. acaciicola* | **CSF24118** | BABB | 1-year-old *E. urophylla × E. grandis* leaf | H | 19º47'41.46"N, 109º49'52.71"E | S. F. Chen, Q. C. Wang, X. Y. Liang and L. F. Liu | *OQ188014* | *OQ210374* | *OQ210637* | *OQ230708* |  |  |
| *C. acaciicola* | CSF24836 | AAAA | Soil (*Eucalyptus* plantation) | H | 19º47'41.46"N, 109º49'52.71"E | S. F. Chen, Q. C. Wang, X. Y. Liang and L. F. Liu | PQ330682 | PQ338549 | PQ330841 | PQ338708 |  |  |
| *C. acaciicola* | CSF24837 | AAAA | Soil (*Eucalyptus* plantation) | H | 19º47'41.46"N, 109º49'52.71"E | S. F. Chen, Q. C. Wang, X. Y. Liang and L. F. Liu | PQ330683 | PQ338550 | PQ330842 | PQ338709 |  |  |
| *C. acaciicola* | CSF24839 | AAAA | Soil (*Eucalyptus* plantation) | H | 19º47'41.46"N, 109º49'52.71"E | S. F. Chen, Q. C. Wang, X. Y. Liang and L. F. Liu | PQ330684 | PQ338551 | PQ330843 | PQ338710 |  |  |
| *C. acaciicola* | CSF24842 | AAAA | Soil (*Eucalyptus* plantation) | H | 19º47'41.46"N, 109º49'52.71"E | S. F. Chen, Q. C. Wang, X. Y. Liang and L. F. Liu | PQ330685 | PQ338552 | PQ330844 | PQ338711 |  |  |
| *C. acaciicola* | CSF24844 ^e^ | CABA | Soil (*Eucalyptus* plantation) | H | 19º47'41.46"N, 109º49'52.71"E | S. F. Chen, Q. C. Wang, X. Y. Liang and L. F. Liu | PQ330686 | PQ338553 | PQ330845 | PQ338712 |  |  |
| *C. acaciicola* | CSF24846 | AAAA | Soil (*Eucalyptus* plantation) | H | 19º47'41.46"N, 109º49'52.71"E | S. F. Chen, Q. C. Wang, X. Y. Liang and L. F. Liu | PQ330687 | PQ338554 | PQ330846 | PQ338713 |  |  |
| *C. acaciicola* | CSF24849 | AAAA | Soil (*Eucalyptus* plantation) | H | 19º47'41.46"N, 109º49'52.71"E | S. F. Chen, Q. C. Wang, X. Y. Liang and L. F. Liu | PQ330688 | PQ338555 | PQ330847 | PQ338714 |  |  |
| *C. acaciicola* | CSF24851 ^e^ | AAAA | Soil (*Eucalyptus* plantation) | H | 19º47'41.46"N, 109º49'52.71"E | S. F. Chen, Q. C. Wang, X. Y. Liang and L. F. Liu | PQ330689 | PQ338556 | PQ330848 | PQ338715 |  |  |
| *C. acaciicola* | CSF24853 | AAAA | Soil (*Eucalyptus* plantation) | H | 19º47'41.46"N, 109º49'52.71"E | S. F. Chen, Q. C. Wang, X. Y. Liang and L. F. Liu | PQ330690 | PQ338557 | PQ330849 | PQ338716 |  |  |
| *C. acaciicola* | CSF24855 | AAAA | Soil (*Eucalyptus* plantation) | H | 19º47'41.46"N, 109º49'52.71"E | S. F. Chen, Q. C. Wang, X. Y. Liang and L. F. Liu | PQ330691 | PQ338558 | PQ330850 | PQ338717 |  |  |
| *C. acaciicola* | CSF24856 | AAAA | Soil (*Eucalyptus* plantation) | H | 19º47'41.46"N, 109º49'52.71"E | S. F. Chen, Q. C. Wang, X. Y. Liang and L. F. Liu | PQ330692 | PQ338559 | PQ330851 | PQ338718 |  |  |
| *C. acaciicola* | CSF24857 | AAAA | Soil (*Eucalyptus* plantation) | H | 19º47'41.46"N, 109º49'52.71"E | S. F. Chen, Q. C. Wang, X. Y. Liang and L. F. Liu | PQ330693 | PQ338560 | PQ330852 | PQ338719 |  |  |
| *C. acaciicola* | CSF24859 | AAAA | Soil (*Eucalyptus* plantation) | H | 19º47'41.46"N, 109º49'52.71"E | S. F. Chen, Q. C. Wang, X. Y. Liang and L. F. Liu | PQ330694 | PQ338561 | PQ330853 | PQ338720 |  |  |
| *C. acaciicola* | CSF24861 | AAAA | Soil (*Eucalyptus* plantation) | H | 19º47'41.46"N, 109º49'52.71"E | S. F. Chen, Q. C. Wang, X. Y. Liang and L. F. Liu | PQ330695 | PQ338562 | PQ330854 | PQ338721 |  |  |
| *C. acaciicola* | CSF24863 | AAAA | Soil (*Eucalyptus* plantation) | H | 19º47'41.46"N, 109º49'52.71"E | S. F. Chen, Q. C. Wang, X. Y. Liang and L. F. Liu | PQ330696 | PQ338563 | PQ330855 | PQ338722 |  |  |
| *C. acaciicola* | CSF24864 | AAAA | Soil (*Eucalyptus* plantation) | H | 19º47'41.46"N, 109º49'52.71"E | S. F. Chen, Q. C. Wang, X. Y. Liang and L. F. Liu | PQ330697 | PQ338564 | PQ330856 | PQ338723 |  |  |
| *C. acaciicola* | CSF24866 | AAAA | Soil (*Eucalyptus* plantation) | H | 19º47'41.46"N, 109º49'52.71"E | S. F. Chen, Q. C. Wang, X. Y. Liang and L. F. Liu | PQ330698 | PQ338565 | PQ330857 | PQ338724 |  |  |
| *C. acaciicola* | CSF24867 | AAAA | Soil (*Eucalyptus* plantation) | H | 19º47'41.46"N, 109º49'52.71"E | S. F. Chen, Q. C. Wang, X. Y. Liang and L. F. Liu | PQ330699 | PQ338566 | PQ330858 | PQ338725 |  |  |
| *C. acaciicola* | CSF24885 ^e^ | BABB | Soil (*Eucalyptus* plantation) | H | 19º47'41.46"N, 109º49'52.71"E | S. F. Chen, Q. C. Wang, X. Y. Liang and L. F. Liu | PQ330700 | PQ338567 | PQ330859 | PQ338726 |  |  |
| *C. acaciicola* | CSF24888 | BABB | Soil (*Eucalyptus* plantation) | H | 19º47'41.46"N, 109º49'52.71"E | S. F. Chen, Q. C. Wang, X. Y. Liang and L. F. Liu | PQ330701 | PQ338568 | PQ330860 | PQ338727 |  |  |
| *C. acaciicola* | CSF24890 | BABB | Soil (*Eucalyptus* plantation) | H | 19º47'41.46"N, 109º49'52.71"E | S. F. Chen, Q. C. Wang, X. Y. Liang and L. F. Liu | PQ330702 | PQ338569 | PQ330861 | PQ338728 |  |  |
| *C. pseudoreteaudii* | CSF24158 ^e^ | AAAA | 1-year-old *Eucalyptus* leaf | A | 21º51'39.78"N, 108º49'52.83"E | S. F. Chen, W. X. Wu, X. Y. Liang and B. Y. Chen | PQ330304 | PQ338171 | PQ330732 | PQ338599 |  |  |
| *C. pseudoreteaudii* | CSF24169 | AA-- | 1-year-old *Eucalyptus* leaf | A | 21º51'39.78"N, 108º49'52.83"E | S. F. Chen, W. X. Wu, X. Y. Liang and B. Y. Chen | PQ330305 | PQ338172 | N/A | N/A |  |  |
| *C. pseudoreteaudii* | CSF24174 | AA-- | 1-year-old *Eucalyptus* leaf | A | 21º51'39.78"N, 108º49'52.83"E | S. F. Chen, W. X. Wu, X. Y. Liang and B. Y. Chen | PQ330306 | PQ338173 | N/A | N/A |  |  |
| *C. pseudoreteaudii* | CSF24176 | AA-- | 1-year-old *Eucalyptus* leaf | A | 21º51'39.78"N, 108º49'52.83"E | S. F. Chen, W. X. Wu, X. Y. Liang and B. Y. Chen | PQ330307 | PQ338174 | N/A | N/A |  |  |
| *C. pseudoreteaudii* | CSF24180 | AAAA | 1-year-old *Eucalyptus* leaf | A | 21º51'39.78"N, 108º49'52.83"E | S. F. Chen, W. X. Wu, X. Y. Liang and B. Y. Chen | PQ330308 | PQ338175 | PQ330733 | PQ338600 |  |  |
| *C. pseudoreteaudii* | CSF24182 | AA-- | 1-year-old *Eucalyptus* leaf | A | 21º51'39.78"N, 108º49'52.83"E | S. F. Chen, W. X. Wu, X. Y. Liang and B. Y. Chen | PQ330309 | PQ338176 | N/A | N/A |  |  |
| *C. pseudoreteaudii* | CSF24184 | AA-- | 1-year-old *Eucalyptus* leaf | A | 21º51'39.78"N, 108º49'52.83"E | S. F. Chen, W. X. Wu, X. Y. Liang and B. Y. Chen | PQ330310 | PQ338177 | N/A | N/A |  |  |
| *C. pseudoreteaudii* | CSF24188 | AA-- | 1-year-old *Eucalyptus* leaf | A | 21º51'39.78"N, 108º49'52.83"E | S. F. Chen, W. X. Wu, X. Y. Liang and B. Y. Chen | PQ330311 | PQ338178 | N/A | N/A |  |  |
| *C. pseudoreteaudii* | CSF24190 | AAAA | 1-year-old *Eucalyptus* leaf | A | 21º51'39.78"N, 108º49'52.83"E | S. F. Chen, W. X. Wu, X. Y. Liang and B. Y. Chen | PQ330312 | PQ338179 | PQ330734 | PQ338601 |  |  |
| *C. pseudoreteaudii* | CSF24194 | AA-- | 1-year-old *Eucalyptus* leaf | A | 21º51'39.78"N, 108º49'52.83"E | S. F. Chen, W. X. Wu, X. Y. Liang and B. Y. Chen | PQ330313 | PQ338180 | N/A | N/A |  |  |
| *C. pseudoreteaudii* | CSF24199 | AA-- | 1-year-old *Eucalyptus* leaf | A | 21º51'39.78"N, 108º49'52.83"E | S. F. Chen, W. X. Wu, X. Y. Liang and B. Y. Chen | PQ330314 | PQ338181 | N/A | N/A |  |  |
| *C. pseudoreteaudii* | CSF24201 | AA-- | 1-year-old *Eucalyptus* leaf | A | 21º51'39.78"N, 108º49'52.83"E | S. F. Chen, W. X. Wu, X. Y. Liang and B. Y. Chen | PQ330315 | PQ338182 | N/A | N/A |  |  |
| *C. pseudoreteaudii* | CSF24202 | AA-- | 1-year-old *Eucalyptus* leaf | A | 21º51'39.78"N, 108º49'52.83"E | S. F. Chen, W. X. Wu, X. Y. Liang and B. Y. Chen | PQ330316 | PQ338183 | N/A | N/A |  |  |
| *C. pseudoreteaudii* | CSF24204 | AA-- | 1-year-old *Eucalyptus* leaf | A | 21º51'39.78"N, 108º49'52.83"E | S. F. Chen, W. X. Wu, X. Y. Liang and B. Y. Chen | PQ330317 | PQ338184 | N/A | N/A |  |  |
| *C. pseudoreteaudii* | CSF24206 | AAAA | 1-year-old *Eucalyptus* leaf | A | 21º51'39.78"N, 108º49'52.83"E | S. F. Chen, W. X. Wu, X. Y. Liang and B. Y. Chen | PQ330318 | PQ338185 | PQ330735 | PQ338602 |  |  |
| *C. pseudoreteaudii* | CSF24209 | AA-- | 1-year-old *Eucalyptus* leaf | A | 21º51'39.78"N, 108º49'52.83"E | S. F. Chen, W. X. Wu, X. Y. Liang and B. Y. Chen | PQ330319 | PQ338186 | N/A | N/A |  |  |
| *C. pseudoreteaudii* | CSF24211 | AA-- | 1-year-old *Eucalyptus* leaf | A | 21º51'39.78"N, 108º49'52.83"E | S. F. Chen, W. X. Wu, X. Y. Liang and B. Y. Chen | PQ330320 | PQ338187 | N/A | N/A |  |  |
| *C. pseudoreteaudii* | CSF24215 | AA-- | 1-year-old *Eucalyptus* leaf | A | 21º51'39.78"N, 108º49'52.83"E | S. F. Chen, W. X. Wu, X. Y. Liang and B. Y. Chen | PQ330321 | PQ338188 | N/A | N/A |  |  |
| *C. pseudoreteaudii* | CSF24217 | AA-- | 1-year-old *Eucalyptus* leaf | A | 21º51'39.78"N, 108º49'52.83"E | S. F. Chen, W. X. Wu, X. Y. Liang and B. Y. Chen | PQ330322 | PQ338189 | N/A | N/A |  |  |
| *C. pseudoreteaudii* | CSF24219 ^e^ | AABA | 1-year-old *Eucalyptus* leaf | A | 21º51'39.78"N, 108º49'52.83"E | S. F. Chen, W. X. Wu, X. Y. Liang and B. Y. Chen | PQ330323 | PQ338190 | PQ330736 | PQ338603 |  |  |
| *C. pseudoreteaudii* | CSF24221 | AAAA | Soil (*Eucalyptus* plantation) | A | 21º51'39.78"N, 108º49'52.83"E | S. F. Chen, W. X. Wu, X. Y. Liang and B. Y. Chen | PQ330324 | PQ338191 | PQ330737 | PQ338604 |  |  |
| *C. pseudoreteaudii* | CSF24242 | AA-- | Soil (*Eucalyptus* plantation) | A | 21º51'39.78"N, 108º49'52.83"E | S. F. Chen, W. X. Wu, X. Y. Liang and B. Y. Chen | PQ330325 | PQ338192 | N/A | N/A |  |  |
| *C. pseudoreteaudii* | CSF24248 | AA-- | Soil (*Eucalyptus* plantation) | A | 21º51'39.78"N, 108º49'52.83"E | S. F. Chen, W. X. Wu, X. Y. Liang and B. Y. Chen | PQ330326 | PQ338193 | N/A | N/A |  |  |
| *C. pseudoreteaudii* | CSF24252 | AAAA | Soil (*Eucalyptus* plantation) | A | 21º51'39.78"N, 108º49'52.83"E | S. F. Chen, W. X. Wu, X. Y. Liang and B. Y. Chen | PQ330327 | PQ338194 | PQ330738 | PQ338605 |  |  |
| *C. pseudoreteaudii* | CSF24261 | AA-- | Soil (*Eucalyptus* plantation) | A | 21º51'39.78"N, 108º49'52.83"E | S. F. Chen, W. X. Wu, X. Y. Liang and B. Y. Chen | PQ330328 | PQ338195 | N/A | N/A |  |  |
| *C. pseudoreteaudii* | CSF24267 | AA-- | Soil (*Eucalyptus* plantation) | A | 21º51'39.78"N, 108º49'52.83"E | S. F. Chen, W. X. Wu, X. Y. Liang and B. Y. Chen | PQ330329 | PQ338196 | N/A | N/A |  |  |
| *C. pseudoreteaudii* | CSF24272 | AA-- | Soil (*Eucalyptus* plantation) | A | 21º51'39.78"N, 108º49'52.83"E | S. F. Chen, W. X. Wu, X. Y. Liang and B. Y. Chen | PQ330330 | PQ338197 | N/A | N/A |  |  |
| *C. pseudoreteaudii* | CSF24280 | AA-- | Soil (*Eucalyptus* plantation) | A | 21º51'39.78"N, 108º49'52.83"E | S. F. Chen, W. X. Wu, X. Y. Liang and B. Y. Chen | PQ330331 | PQ338198 | N/A | N/A |  |  |
| *C. pseudoreteaudii* | CSF24286 | AA-- | Soil (*Eucalyptus* plantation) | A | 21º51'39.78"N, 108º49'52.83"E | S. F. Chen, W. X. Wu, X. Y. Liang and B. Y. Chen | PQ330332 | PQ338199 | N/A | N/A |  |  |
| *C. pseudoreteaudii* | CSF24298 ^e^ | AAAA | Soil (*Eucalyptus* plantation) | A | 21º51'39.78"N, 108º49'52.83"E | S. F. Chen, W. X. Wu, X. Y. Liang and B. Y. Chen | PQ330333 | PQ338200 | PQ330739 | PQ338606 |  |  |
| *C. pseudoreteaudii* | CSF24307 | AA-- | Soil (*Eucalyptus* plantation) | A | 21º51'39.78"N, 108º49'52.83"E | S. F. Chen, W. X. Wu, X. Y. Liang and B. Y. Chen | PQ330334 | PQ338201 | N/A | N/A |  |  |
| *C. pseudoreteaudii* | CSF24313 | AA-- | Soil (*Eucalyptus* plantation) | A | 21º51'39.78"N, 108º49'52.83"E | S. F. Chen, W. X. Wu, X. Y. Liang and B. Y. Chen | PQ330335 | PQ338202 | N/A | N/A |  |  |
| *C. pseudoreteaudii* | CSF24316 | AA-- | Soil (*Eucalyptus* plantation) | A | 21º51'39.78"N, 108º49'52.83"E | S. F. Chen, W. X. Wu, X. Y. Liang and B. Y. Chen | PQ330336 | PQ338203 | N/A | N/A |  |  |
| *C. pseudoreteaudii* | CSF24322 | AA-- | Soil (*Eucalyptus* plantation) | A | 21º51'39.78"N, 108º49'52.83"E | S. F. Chen, W. X. Wu, X. Y. Liang and B. Y. Chen | PQ330337 | PQ338204 | N/A | N/A |  |  |
| *C. pseudoreteaudii* | CSF24328 | AAAA | Soil (*Eucalyptus* plantation) | A | 21º51'39.78"N, 108º49'52.83"E | S. F. Chen, W. X. Wu, X. Y. Liang and B. Y. Chen | PQ330338 | PQ338205 | PQ330740 | PQ338607 |  |  |
| *C. pseudoreteaudii* | CSF24334 | AA-- | Soil (*Eucalyptus* plantation) | A | 21º51'39.78"N, 108º49'52.83"E | S. F. Chen, W. X. Wu, X. Y. Liang and B. Y. Chen | PQ330339 | PQ338206 | N/A | N/A |  |  |
| *C. pseudoreteaudii* | CSF24340 | AA-- | Soil (*Eucalyptus* plantation) | A | 21º51'39.78"N, 108º49'52.83"E | S. F. Chen, W. X. Wu, X. Y. Liang and B. Y. Chen | PQ330340 | PQ338207 | N/A | N/A |  |  |
| *C. pseudoreteaudii* | CSF24348 | AA-- | Soil (*Eucalyptus* plantation) | A | 21º51'39.78"N, 108º49'52.83"E | S. F. Chen, W. X. Wu, X. Y. Liang and B. Y. Chen | PQ330341 | PQ338208 | N/A | N/A |  |  |
| *C. pseudoreteaudii* | CSF24354 | AA-- | Soil (*Eucalyptus* plantation) | A | 21º51'39.78"N, 108º49'52.83"E | S. F. Chen, W. X. Wu, X. Y. Liang and B. Y. Chen | PQ330342 | PQ338209 | N/A | N/A |  |  |
| *C. pseudoreteaudii* | CSF24359 | AABA | Soil (*Eucalyptus* plantation) | A | 21º51'39.78"N, 108º49'52.83"E | S. F. Chen, W. X. Wu, X. Y. Liang and B. Y. Chen | PQ330343 | PQ338210 | PQ330741 | PQ338608 |  |  |
| *C. pseudoreteaudii* | CSF24361 | AAAA | 1-year-old *Eucalyptus* leaf | B | 21º51'20.64"N, 108º55'26.70"E | S. F. Chen, W. X. Wu, X. Y. Liang and B. Y. Chen | PQ330344 | PQ338211 | PQ330742 | PQ338609 |  |  |
| *C. pseudoreteaudii* | CSF24362 | AA-- | 1-year-old *Eucalyptus* leaf | B | 21º51'20.64"N, 108º55'26.70"E | S. F. Chen, W. X. Wu, X. Y. Liang and B. Y. Chen | PQ330345 | PQ338212 | N/A | N/A |  |  |
| *C. pseudoreteaudii* | CSF24363 | AA-- | 1-year-old *Eucalyptus* leaf | B | 21º51'20.64"N, 108º55'26.70"E | S. F. Chen, W. X. Wu, X. Y. Liang and B. Y. Chen | PQ330346 | PQ338213 | N/A | N/A |  |  |
| *C. pseudoreteaudii* | CSF24364 | AA-- | 1-year-old *Eucalyptus* leaf | B | 21º51'20.64"N, 108º55'26.70"E | S. F. Chen, W. X. Wu, X. Y. Liang and B. Y. Chen | PQ330347 | PQ338214 | N/A | N/A |  |  |
| *C. pseudoreteaudii* | CSF24365 | AA-- | 1-year-old *Eucalyptus* leaf | B | 21º51'20.64"N, 108º55'26.70"E | S. F. Chen, W. X. Wu, X. Y. Liang and B. Y. Chen | PQ330348 | PQ338215 | N/A | N/A |  |  |
| *C. pseudoreteaudii* | CSF24366 | AA-- | 1-year-old *Eucalyptus* leaf | B | 21º51'20.64"N, 108º55'26.70"E | S. F. Chen, W. X. Wu, X. Y. Liang and B. Y. Chen | PQ330349 | PQ338216 | N/A | N/A |  |  |
| *C. pseudoreteaudii* | CSF24367 | AAAA | 1-year-old *Eucalyptus* leaf | B | 21º51'20.64"N, 108º55'26.70"E | S. F. Chen, W. X. Wu, X. Y. Liang and B. Y. Chen | PQ330350 | PQ338217 | PQ330743 | PQ338610 |  |  |
| *C. pseudoreteaudii* | CSF24368 | AA-- | 1-year-old *Eucalyptus* leaf | B | 21º51'20.64"N, 108º55'26.70"E | S. F. Chen, W. X. Wu, X. Y. Liang and B. Y. Chen | PQ330351 | PQ338218 | N/A | N/A |  |  |
| *C. pseudoreteaudii* | CSF24369 | AA-- | 1-year-old *Eucalyptus* leaf | B | 21º51'20.64"N, 108º55'26.70"E | S. F. Chen, W. X. Wu, X. Y. Liang and B. Y. Chen | PQ330352 | PQ338219 | N/A | N/A |  |  |
| *C. pseudoreteaudii* | CSF24370 | AA-- | 1-year-old *Eucalyptus* leaf | B | 21º51'20.64"N, 108º55'26.70"E | S. F. Chen, W. X. Wu, X. Y. Liang and B. Y. Chen | PQ330353 | PQ338220 | N/A | N/A |  |  |
| *C. pseudoreteaudii* | CSF24371 | AAAA | 1-year-old *Eucalyptus* leaf | B | 21º51'20.64"N, 108º55'26.70"E | S. F. Chen, W. X. Wu, X. Y. Liang and B. Y. Chen | PQ330354 | PQ338221 | PQ330744 | PQ338611 |  |  |
| *C. pseudoreteaudii* | CSF24372 | AA-- | 1-year-old *Eucalyptus* leaf | B | 21º51'20.64"N, 108º55'26.70"E | S. F. Chen, W. X. Wu, X. Y. Liang and B. Y. Chen | PQ330355 | PQ338222 | N/A | N/A |  |  |
| *C. pseudoreteaudii* | CSF24373 | AA-- | 1-year-old *Eucalyptus* leaf | B | 21º51'20.64"N, 108º55'26.70"E | S. F. Chen, W. X. Wu, X. Y. Liang and B. Y. Chen | PQ330356 | PQ338223 | N/A | N/A |  |  |
| *C. pseudoreteaudii* | CSF24374 | AA-- | 1-year-old *Eucalyptus* leaf | B | 21º51'20.64"N, 108º55'26.70"E | S. F. Chen, W. X. Wu, X. Y. Liang and B. Y. Chen | PQ330357 | PQ338224 | N/A | N/A |  |  |
| *C. pseudoreteaudii* | CSF24375 | AA-- | 1-year-old *Eucalyptus* leaf | B | 21º51'20.64"N, 108º55'26.70"E | S. F. Chen, W. X. Wu, X. Y. Liang and B. Y. Chen | PQ330358 | PQ338225 | N/A | N/A |  |  |
| *C. pseudoreteaudii* | CSF24376 | AAAA | 1-year-old *Eucalyptus* leaf | B | 21º51'20.64"N, 108º55'26.70"E | S. F. Chen, W. X. Wu, X. Y. Liang and B. Y. Chen | PQ330359 | PQ338226 | PQ330745 | PQ338612 |  |  |
| *C. pseudoreteaudii* | CSF24377 | AA-- | 1-year-old *Eucalyptus* leaf | B | 21º51'20.64"N, 108º55'26.70"E | S. F. Chen, W. X. Wu, X. Y. Liang and B. Y. Chen | PQ330360 | PQ338227 | N/A | N/A |  |  |
| *C. pseudoreteaudii* | CSF24378 | AA-- | 1-year-old *Eucalyptus* leaf | B | 21º51'20.64"N, 108º55'26.70"E | S. F. Chen, W. X. Wu, X. Y. Liang and B. Y. Chen | PQ330361 | PQ338228 | N/A | N/A |  |  |
| *C. pseudoreteaudii* | CSF24379 | AA-- | 1-year-old *Eucalyptus* leaf | B | 21º51'20.64"N, 108º55'26.70"E | S. F. Chen, W. X. Wu, X. Y. Liang and B. Y. Chen | PQ330362 | PQ338229 | N/A | N/A |  |  |
| *C. pseudoreteaudii* | CSF24380 | AA-- | 1-year-old *Eucalyptus* leaf | B | 21º51'20.64"N, 108º55'26.70"E | S. F. Chen, W. X. Wu, X. Y. Liang and B. Y. Chen | PQ330363 | PQ338230 | N/A | N/A |  |  |
| *C. pseudoreteaudii* | CSF24381 | AA-- | 1-year-old *Eucalyptus* leaf | B | 21º51'20.64"N, 108º55'26.70"E | S. F. Chen, W. X. Wu, X. Y. Liang and B. Y. Chen | PQ330364 | PQ338231 | N/A | N/A |  |  |
| *C. pseudoreteaudii* | CSF24382 | AA-- | 1-year-old *Eucalyptus* leaf | B | 21º51'20.64"N, 108º55'26.70"E | S. F. Chen, W. X. Wu, X. Y. Liang and B. Y. Chen | PQ330365 | PQ338232 | N/A | N/A |  |  |
| *C. pseudoreteaudii* | CSF24383 | AAAA | 1-year-old *Eucalyptus* leaf | B | 21º51'20.64"N, 108º55'26.70"E | S. F. Chen, W. X. Wu, X. Y. Liang and B. Y. Chen | PQ330366 | PQ338233 | PQ330746 | PQ338613 |  |  |
| *C. pseudoreteaudii* | CSF24384 | AA-- | 1-year-old *Eucalyptus* leaf | B | 21º51'20.64"N, 108º55'26.70"E | S. F. Chen, W. X. Wu, X. Y. Liang and B. Y. Chen | PQ330367 | PQ338234 | N/A | N/A |  |  |
| *C. pseudoreteaudii* | CSF24385 | AA-- | 1-year-old *Eucalyptus* leaf | B | 21º51'20.64"N, 108º55'26.70"E | S. F. Chen, W. X. Wu, X. Y. Liang and B. Y. Chen | PQ330368 | PQ338235 | N/A | N/A |  |  |
| *C. pseudoreteaudii* | CSF24386 | AA-- | 1-year-old *Eucalyptus* leaf | B | 21º51'20.64"N, 108º55'26.70"E | S. F. Chen, W. X. Wu, X. Y. Liang and B. Y. Chen | PQ330369 | PQ338236 | N/A | N/A |  |  |
| *C. pseudoreteaudii* | CSF24387 | AA-- | 1-year-old *Eucalyptus* leaf | B | 21º51'20.64"N, 108º55'26.70"E | S. F. Chen, W. X. Wu, X. Y. Liang and B. Y. Chen | PQ330370 | PQ338237 | N/A | N/A |  |  |
| *C. pseudoreteaudii* | CSF24388 | AA-- | 1-year-old *Eucalyptus* leaf | B | 21º51'20.64"N, 108º55'26.70"E | S. F. Chen, W. X. Wu, X. Y. Liang and B. Y. Chen | PQ330371 | PQ338238 | N/A | N/A |  |  |
| *C. pseudoreteaudii* | CSF24389 | AA-- | 1-year-old *Eucalyptus* leaf | B | 21º51'20.64"N, 108º55'26.70"E | S. F. Chen, W. X. Wu, X. Y. Liang and B. Y. Chen | PQ330372 | PQ338239 | N/A | N/A |  |  |
| *C. pseudoreteaudii* | CSF24390 | AAAA | 1-year-old *Eucalyptus* leaf | B | 21º51'20.64"N, 108º55'26.70"E | S. F. Chen, W. X. Wu, X. Y. Liang and B. Y. Chen | PQ330373 | PQ338240 | PQ330747 | PQ338614 |  |  |
| *C. pseudoreteaudii* | CSF24391 | AA-- | 1-year-old *Eucalyptus* leaf | B | 21º51'20.64"N, 108º55'26.70"E | S. F. Chen, W. X. Wu, X. Y. Liang and B. Y. Chen | PQ330374 | PQ338241 | N/A | N/A |  |  |
| *C. pseudoreteaudii* | CSF24392 | AA-- | 1-year-old *Eucalyptus* leaf | B | 21º51'20.64"N, 108º55'26.70"E | S. F. Chen, W. X. Wu, X. Y. Liang and B. Y. Chen | PQ330375 | PQ338242 | N/A | N/A |  |  |
| *C. pseudoreteaudii* | CSF24393 | AA-- | 1-year-old *Eucalyptus* leaf | B | 21º51'20.64"N, 108º55'26.70"E | S. F. Chen, W. X. Wu, X. Y. Liang and B. Y. Chen | PQ330376 | PQ338243 | N/A | N/A |  |  |
| *C. pseudoreteaudii* | CSF24394 | AAAA | 1-year-old *Eucalyptus* leaf | B | 21º51'20.64"N, 108º55'26.70"E | S. F. Chen, W. X. Wu, X. Y. Liang and B. Y. Chen | PQ330377 | PQ338244 | PQ330748 | PQ338615 |  |  |
| *C. pseudoreteaudii* | CSF24395 | AA-- | 1-year-old *Eucalyptus* leaf | B | 21º51'20.64"N, 108º55'26.70"E | S. F. Chen, W. X. Wu, X. Y. Liang and B. Y. Chen | PQ330378 | PQ338245 | N/A | N/A |  |  |
| *C. pseudoreteaudii* | CSF24396 | AA-- | 1-year-old *Eucalyptus* leaf | B | 21º51'20.64"N, 108º55'26.70"E | S. F. Chen, W. X. Wu, X. Y. Liang and B. Y. Chen | PQ330379 | PQ338246 | N/A | N/A |  |  |
| *C. pseudoreteaudii* | CSF24397 | AA-- | 1-year-old *Eucalyptus* leaf | B | 21º51'20.64"N, 108º55'26.70"E | S. F. Chen, W. X. Wu, X. Y. Liang and B. Y. Chen | PQ330380 | PQ338247 | N/A | N/A |  |  |
| *C. pseudoreteaudii* | CSF24398 | AAAA | 1-year-old *Eucalyptus* leaf | B | 21º51'20.64"N, 108º55'26.70"E | S. F. Chen, W. X. Wu, X. Y. Liang and B. Y. Chen | PQ330381 | PQ338248 | PQ330749 | PQ338616 |  |  |
| *C. pseudoreteaudii* | CSF24399 | AA-- | 1-year-old *Eucalyptus* leaf | B | 21º51'20.64"N, 108º55'26.70"E | S. F. Chen, W. X. Wu, X. Y. Liang and B. Y. Chen | PQ330382 | PQ338249 | N/A | N/A |  |  |
| *C. pseudoreteaudii* | CSF24400 | AA-- | 1-year-old *Eucalyptus* leaf | B | 21º51'20.64"N, 108º55'26.70"E | S. F. Chen, W. X. Wu, X. Y. Liang and B. Y. Chen | PQ330383 | PQ338250 | N/A | N/A |  |  |
| *C. pseudoreteaudii* | CSF24401 | AA-- | 1-year-old *Eucalyptus* leaf | B | 21º51'20.64"N, 108º55'26.70"E | S. F. Chen, W. X. Wu, X. Y. Liang and B. Y. Chen | PQ330384 | PQ338251 | N/A | N/A |  |  |
| *C. pseudoreteaudii* | CSF24402 | AA-- | 1-year-old *Eucalyptus* leaf | B | 21º51'20.64"N, 108º55'26.70"E | S. F. Chen, W. X. Wu, X. Y. Liang and B. Y. Chen | PQ330385 | PQ338252 | N/A | N/A |  |  |
| *C. pseudoreteaudii* | CSF24403 | AA-- | 1-year-old *Eucalyptus* leaf | B | 21º51'20.64"N, 108º55'26.70"E | S. F. Chen, W. X. Wu, X. Y. Liang and B. Y. Chen | PQ330386 | PQ338253 | N/A | N/A |  |  |
| *C. pseudoreteaudii* | CSF24404 | AAAA | 1-year-old *Eucalyptus* leaf | B | 21º51'20.64"N, 108º55'26.70"E | S. F. Chen, W. X. Wu, X. Y. Liang and B. Y. Chen | PQ330387 | PQ338254 | PQ330750 | PQ338617 |  |  |
| *C. pseudoreteaudii* | CSF24405 | AA-- | 1-year-old *Eucalyptus* leaf | B | 21º51'20.64"N, 108º55'26.70"E | S. F. Chen, W. X. Wu, X. Y. Liang and B. Y. Chen | PQ330388 | PQ338255 | N/A | N/A |  |  |
| *C. pseudoreteaudii* | CSF24406 | AA-- | 1-year-old *Eucalyptus* leaf | B | 21º51'20.64"N, 108º55'26.70"E | S. F. Chen, W. X. Wu, X. Y. Liang and B. Y. Chen | PQ330389 | PQ338256 | N/A | N/A |  |  |
| *C. pseudoreteaudii* | CSF24407 | AA-- | 1-year-old *Eucalyptus* leaf | B | 21º51'20.64"N, 108º55'26.70"E | S. F. Chen, W. X. Wu, X. Y. Liang and B. Y. Chen | PQ330390 | PQ338257 | N/A | N/A |  |  |
| *C. pseudoreteaudii* | CSF24408 | AA-- | 1-year-old *Eucalyptus* leaf | B | 21º51'20.64"N, 108º55'26.70"E | S. F. Chen, W. X. Wu, X. Y. Liang and B. Y. Chen | PQ330391 | PQ338258 | N/A | N/A |  |  |
| *C. pseudoreteaudii* | CSF24409 | AA-- | 1-year-old *Eucalyptus* leaf | B | 21º51'20.64"N, 108º55'26.70"E | S. F. Chen, W. X. Wu, X. Y. Liang and B. Y. Chen | PQ330392 | PQ338259 | N/A | N/A |  |  |
| *C. pseudoreteaudii* | CSF24410 | AAAA | 1-year-old *Eucalyptus* leaf | B | 21º51'20.64"N, 108º55'26.70"E | S. F. Chen, W. X. Wu, X. Y. Liang and B. Y. Chen | PQ330393 | PQ338260 | PQ330751 | PQ338618 |  |  |
| *C. pseudoreteaudii* | CSF24411 | AA-- | Soil (*Eucalyptus* plantation) | B | 21º51'20.64"N, 108º55'26.70"E | S. F. Chen, W. X. Wu, X. Y. Liang and B. Y. Chen | PQ330394 | PQ338261 | N/A | N/A |  |  |
| *C. pseudoreteaudii* | CSF24413 | AAAA | Soil (*Eucalyptus* plantation) | B | 21º51'20.64"N, 108º55'26.70"E | S. F. Chen, W. X. Wu, X. Y. Liang and B. Y. Chen | PQ330395 | PQ338262 | PQ330752 | PQ338619 |  |  |
| *C. pseudoreteaudii* | CSF24414 | AA-- | Soil (*Eucalyptus* plantation) | B | 21º51'20.64"N, 108º55'26.70"E | S. F. Chen, W. X. Wu, X. Y. Liang and B. Y. Chen | PQ330396 | PQ338263 | N/A | N/A |  |  |
| *C. pseudoreteaudii* | CSF24417 | AA-- | Soil (*Eucalyptus* plantation) | B | 21º51'20.64"N, 108º55'26.70"E | S. F. Chen, W. X. Wu, X. Y. Liang and B. Y. Chen | PQ330397 | PQ338264 | N/A | N/A |  |  |
| *C. pseudoreteaudii* | CSF24419 | AA-- | Soil (*Eucalyptus* plantation) | B | 21º51'20.64"N, 108º55'26.70"E | S. F. Chen, W. X. Wu, X. Y. Liang and B. Y. Chen | PQ330398 | PQ338265 | N/A | N/A |  |  |
| *C. pseudoreteaudii* | CSF24420 | AAAA | Soil (*Eucalyptus* plantation) | B | 21º51'20.64"N, 108º55'26.70"E | S. F. Chen, W. X. Wu, X. Y. Liang and B. Y. Chen | PQ330399 | PQ338266 | PQ330753 | PQ338620 |  |  |
| *C. pseudoreteaudii* | CSF24422 | AA-- | Soil (*Eucalyptus* plantation) | B | 21º51'20.64"N, 108º55'26.70"E | S. F. Chen, W. X. Wu, X. Y. Liang and B. Y. Chen | PQ330400 | PQ338267 | N/A | N/A |  |  |
| *C. pseudoreteaudii* | CSF24423 | AA-- | Soil (*Eucalyptus* plantation) | B | 21º51'20.64"N, 108º55'26.70"E | S. F. Chen, W. X. Wu, X. Y. Liang and B. Y. Chen | PQ330401 | PQ338268 | N/A | N/A |  |  |
| *C. pseudoreteaudii* | CSF24426 | AA-- | Soil (*Eucalyptus* plantation) | B | 21º51'20.64"N, 108º55'26.70"E | S. F. Chen, W. X. Wu, X. Y. Liang and B. Y. Chen | PQ330402 | PQ338269 | N/A | N/A |  |  |
| *C. pseudoreteaudii* | CSF24429 | AAAA | Soil (*Eucalyptus* plantation) | B | 21º51'20.64"N, 108º55'26.70"E | S. F. Chen, W. X. Wu, X. Y. Liang and B. Y. Chen | PQ330403 | PQ338270 | PQ330754 | PQ338621 |  |  |
| *C. pseudoreteaudii* | CSF24432 | AA-- | Soil (*Eucalyptus* plantation) | B | 21º51'20.64"N, 108º55'26.70"E | S. F. Chen, W. X. Wu, X. Y. Liang and B. Y. Chen | PQ330404 | PQ338271 | N/A | N/A |  |  |
| *C. pseudoreteaudii* | CSF24435 | AA-- | Soil (*Eucalyptus* plantation) | B | 21º51'20.64"N, 108º55'26.70"E | S. F. Chen, W. X. Wu, X. Y. Liang and B. Y. Chen | PQ330405 | PQ338272 | N/A | N/A |  |  |
| *C. pseudoreteaudii* | CSF24436 | AA-- | Soil (*Eucalyptus* plantation) | B | 21º51'20.64"N, 108º55'26.70"E | S. F. Chen, W. X. Wu, X. Y. Liang and B. Y. Chen | PQ330406 | PQ338273 | N/A | N/A |  |  |
| *C. pseudoreteaudii* | CSF24437 | AA-- | Soil (*Eucalyptus* plantation) | B | 21º51'20.64"N, 108º55'26.70"E | S. F. Chen, W. X. Wu, X. Y. Liang and B. Y. Chen | PQ330407 | PQ338274 | N/A | N/A |  |  |
| *C. pseudoreteaudii* | CSF24439 | AAAA | Soil (*Eucalyptus* plantation) | B | 21º51'20.64"N, 108º55'26.70"E | S. F. Chen, W. X. Wu, X. Y. Liang and B. Y. Chen | PQ330408 | PQ338275 | PQ330755 | PQ338622 |  |  |
| *C. pseudoreteaudii* | CSF24442 | AA-- | Soil (*Eucalyptus* plantation) | B | 21º51'20.64"N, 108º55'26.70"E | S. F. Chen, W. X. Wu, X. Y. Liang and B. Y. Chen | PQ330409 | PQ338276 | N/A | N/A |  |  |
| *C. pseudoreteaudii* | CSF24445 | AA-- | Soil (*Eucalyptus* plantation) | B | 21º51'20.64"N, 108º55'26.70"E | S. F. Chen, W. X. Wu, X. Y. Liang and B. Y. Chen | PQ330410 | PQ338277 | N/A | N/A |  |  |
| *C. pseudoreteaudii* | CSF24448 | AA-- | Soil (*Eucalyptus* plantation) | B | 21º51'20.64"N, 108º55'26.70"E | S. F. Chen, W. X. Wu, X. Y. Liang and B. Y. Chen | PQ330411 | PQ338278 | N/A | N/A |  |  |
| *C. pseudoreteaudii* | CSF24451 | AAAA | Soil (*Eucalyptus* plantation) | B | 21º51'20.64"N, 108º55'26.70"E | S. F. Chen, W. X. Wu, X. Y. Liang and B. Y. Chen | PQ330412 | PQ338279 | PQ330756 | PQ338623 |  |  |
| *C. pseudoreteaudii* | CSF24454 | AAAA | 1-year-old *Eucalyptus* leaf | C | 21º50'31.94"N, 108º57'37.67"E | S. F. Chen, W. X. Wu, X. Y. Liang and B. Y. Chen | PQ330413 | PQ338280 | PQ330757 | PQ338624 |  |  |
| *C. pseudoreteaudii* | CSF24456 | AA-- | 1-year-old *Eucalyptus* leaf | C | 21º50'31.94"N, 108º57'37.67"E | S. F. Chen, W. X. Wu, X. Y. Liang and B. Y. Chen | PQ330414 | PQ338281 | N/A | N/A |  |  |
| *C. pseudoreteaudii* | CSF24461 | AA-- | 1-year-old *Eucalyptus* leaf | C | 21º50'31.94"N, 108º57'37.67"E | S. F. Chen, W. X. Wu, X. Y. Liang and B. Y. Chen | PQ330415 | PQ338282 | N/A | N/A |  |  |
| *C. pseudoreteaudii* | CSF24464 | AA-- | 1-year-old *Eucalyptus* leaf | C | 21º50'31.94"N, 108º57'37.67"E | S. F. Chen, W. X. Wu, X. Y. Liang and B. Y. Chen | PQ330416 | PQ338283 | N/A | N/A |  |  |
| *C. pseudoreteaudii* | CSF24470 | AAAA | 1-year-old *Eucalyptus* leaf | C | 21º50'31.94"N, 108º57'37.67"E | S. F. Chen, W. X. Wu, X. Y. Liang and B. Y. Chen | PQ330417 | PQ338284 | PQ330758 | PQ338625 |  |  |
| *C. pseudoreteaudii* | CSF24471 | AA-- | 1-year-old *Eucalyptus* leaf | C | 21º50'31.94"N, 108º57'37.67"E | S. F. Chen, W. X. Wu, X. Y. Liang and B. Y. Chen | PQ330418 | PQ338285 | N/A | N/A |  |  |
| *C. pseudoreteaudii* | CSF24475 | AA-- | 1-year-old *Eucalyptus* leaf | C | 21º50'31.94"N, 108º57'37.67"E | S. F. Chen, W. X. Wu, X. Y. Liang and B. Y. Chen | PQ330419 | PQ338286 | N/A | N/A |  |  |
| *C. pseudoreteaudii* | CSF24477 | AA-- | 1-year-old *Eucalyptus* leaf | C | 21º50'31.94"N, 108º57'37.67"E | S. F. Chen, W. X. Wu, X. Y. Liang and B. Y. Chen | PQ330420 | PQ338287 | N/A | N/A |  |  |
| *C. pseudoreteaudii* | CSF24481 | AA-- | 1-year-old *Eucalyptus* leaf | C | 21º50'31.94"N, 108º57'37.67"E | S. F. Chen, W. X. Wu, X. Y. Liang and B. Y. Chen | PQ330421 | PQ338288 | N/A | N/A |  |  |
| *C. pseudoreteaudii* | CSF24483 | AAAA | 1-year-old *Eucalyptus* leaf | C | 21º50'31.94"N, 108º57'37.67"E | S. F. Chen, W. X. Wu, X. Y. Liang and B. Y. Chen | PQ330422 | PQ338289 | PQ330759 | PQ338626 |  |  |
| *C. pseudoreteaudii* | CSF24485 | AA-- | 1-year-old *Eucalyptus* leaf | C | 21º50'31.94"N, 108º57'37.67"E | S. F. Chen, W. X. Wu, X. Y. Liang and B. Y. Chen | PQ330423 | PQ338290 | N/A | N/A |  |  |
| *C. pseudoreteaudii* | CSF24487 | AA-- | 1-year-old *Eucalyptus* leaf | C | 21º50'31.94"N, 108º57'37.67"E | S. F. Chen, W. X. Wu, X. Y. Liang and B. Y. Chen | PQ330424 | PQ338291 | N/A | N/A |  |  |
| *C. pseudoreteaudii* | CSF24489 | AA-- | 1-year-old *Eucalyptus* leaf | C | 21º50'31.94"N, 108º57'37.67"E | S. F. Chen, W. X. Wu, X. Y. Liang and B. Y. Chen | PQ330425 | PQ338292 | N/A | N/A |  |  |
| *C. pseudoreteaudii* | CSF24492 | AA-- | 1-year-old *Eucalyptus* leaf | C | 21º50'31.94"N, 108º57'37.67"E | S. F. Chen, W. X. Wu, X. Y. Liang and B. Y. Chen | PQ330426 | PQ338293 | N/A | N/A |  |  |
| *C. pseudoreteaudii* | CSF24494 | AAAA | 1-year-old *Eucalyptus* leaf | C | 21º50'31.94"N, 108º57'37.67"E | S. F. Chen, W. X. Wu, X. Y. Liang and B. Y. Chen | PQ330427 | PQ338294 | PQ330760 | PQ338627 |  |  |
| *C. pseudoreteaudii* | CSF24496 | AA-- | 1-year-old *Eucalyptus* leaf | C | 21º50'31.94"N, 108º57'37.67"E | S. F. Chen, W. X. Wu, X. Y. Liang and B. Y. Chen | PQ330428 | PQ338295 | N/A | N/A |  |  |
| *C. pseudoreteaudii* | CSF24497 | AA-- | 1-year-old *Eucalyptus* leaf | C | 21º50'31.94"N, 108º57'37.67"E | S. F. Chen, W. X. Wu, X. Y. Liang and B. Y. Chen | PQ330429 | PQ338296 | N/A | N/A |  |  |
| *C. pseudoreteaudii* | CSF24500 | AA-- | 1-year-old *Eucalyptus* leaf | C | 21º50'31.94"N, 108º57'37.67"E | S. F. Chen, W. X. Wu, X. Y. Liang and B. Y. Chen | PQ330430 | PQ338297 | N/A | N/A |  |  |
| *C. pseudoreteaudii* | CSF24501 | AA-- | 1-year-old *Eucalyptus* leaf | C | 21º50'31.94"N, 108º57'37.67"E | S. F. Chen, W. X. Wu, X. Y. Liang and B. Y. Chen | PQ330431 | PQ338298 | N/A | N/A |  |  |
| *C. pseudoreteaudii* | CSF24503 | AAAA | 1-year-old *Eucalyptus* leaf | C | 21º50'31.94"N, 108º57'37.67"E | S. F. Chen, W. X. Wu, X. Y. Liang and B. Y. Chen | PQ330432 | PQ338299 | PQ330761 | PQ338628 |  |  |
| *C. pseudoreteaudii* | CSF24505 | AAAA | Soil (*Eucalyptus* plantation) | C | 21º50'31.94"N, 108º57'37.67"E | S. F. Chen, W. X. Wu, X. Y. Liang and B. Y. Chen | PQ330433 | PQ338300 | PQ330762 | PQ338629 |  |  |
| *C. pseudoreteaudii* | CSF24508 | AA-- | Soil (*Eucalyptus* plantation) | C | 21º50'31.94"N, 108º57'37.67"E | S. F. Chen, W. X. Wu, X. Y. Liang and B. Y. Chen | PQ330434 | PQ338301 | N/A | N/A |  |  |
| *C. pseudoreteaudii* | CSF24511 | AA-- | Soil (*Eucalyptus* plantation) | C | 21º50'31.94"N, 108º57'37.67"E | S. F. Chen, W. X. Wu, X. Y. Liang and B. Y. Chen | PQ330435 | PQ338302 | N/A | N/A |  |  |
| *C. pseudoreteaudii* | CSF24513 | AA-- | Soil (*Eucalyptus* plantation) | C | 21º50'31.94"N, 108º57'37.67"E | S. F. Chen, W. X. Wu, X. Y. Liang and B. Y. Chen | PQ330436 | PQ338303 | N/A | N/A |  |  |
| *C. pseudoreteaudii* | CSF24516 | AAAA | Soil (*Eucalyptus* plantation) | C | 21º50'31.94"N, 108º57'37.67"E | S. F. Chen, W. X. Wu, X. Y. Liang and B. Y. Chen | PQ330437 | PQ338304 | PQ330763 | PQ338630 |  |  |
| *C. pseudoreteaudii* | CSF24518 | AA-- | Soil (*Eucalyptus* plantation) | C | 21º50'31.94"N, 108º57'37.67"E | S. F. Chen, W. X. Wu, X. Y. Liang and B. Y. Chen | PQ330438 | PQ338305 | N/A | N/A |  |  |
| *C. pseudoreteaudii* | CSF24521 | AA-- | Soil (*Eucalyptus* plantation) | C | 21º50'31.94"N, 108º57'37.67"E | S. F. Chen, W. X. Wu, X. Y. Liang and B. Y. Chen | PQ330439 | PQ338306 | N/A | N/A |  |  |
| *C. pseudoreteaudii* | CSF24524 | AA-- | Soil (*Eucalyptus* plantation) | C | 21º50'31.94"N, 108º57'37.67"E | S. F. Chen, W. X. Wu, X. Y. Liang and B. Y. Chen | PQ330440 | PQ338307 | N/A | N/A |  |  |
| *C. pseudoreteaudii* | CSF24526 | AAAA | Soil (*Eucalyptus* plantation) | C | 21º50'31.94"N, 108º57'37.67"E | S. F. Chen, W. X. Wu, X. Y. Liang and B. Y. Chen | PQ330441 | PQ338308 | PQ330764 | PQ338631 |  |  |
| *C. pseudoreteaudii* | CSF24529 | AA-- | Soil (*Eucalyptus* plantation) | C | 21º50'31.94"N, 108º57'37.67"E | S. F. Chen, W. X. Wu, X. Y. Liang and B. Y. Chen | PQ330442 | PQ338309 | N/A | N/A |  |  |
| *C. pseudoreteaudii* | CSF24532 | AA-- | Soil (*Eucalyptus* plantation) | C | 21º50'31.94"N, 108º57'37.67"E | S. F. Chen, W. X. Wu, X. Y. Liang and B. Y. Chen | PQ330443 | PQ338310 | N/A | N/A |  |  |
| *C. pseudoreteaudii* | CSF24536 | AA-- | Soil (*Eucalyptus* plantation) | C | 21º50'31.94"N, 108º57'37.67"E | S. F. Chen, W. X. Wu, X. Y. Liang and B. Y. Chen | PQ330444 | PQ338311 | N/A | N/A |  |  |
| *C. pseudoreteaudii* | CSF24539 | AAAA | Soil (*Eucalyptus* plantation) | C | 21º50'31.94"N, 108º57'37.67"E | S. F. Chen, W. X. Wu, X. Y. Liang and B. Y. Chen | PQ330445 | PQ338312 | PQ330765 | PQ338632 |  |  |
| *C. pseudoreteaudii* | CSF24542 | AA-- | Soil (*Eucalyptus* plantation) | C | 21º50'31.94"N, 108º57'37.67"E | S. F. Chen, W. X. Wu, X. Y. Liang and B. Y. Chen | PQ330446 | PQ338313 | N/A | N/A |  |  |
| *C. pseudoreteaudii* | CSF24545 | AA-- | Soil (*Eucalyptus* plantation) | C | 21º50'31.94"N, 108º57'37.67"E | S. F. Chen, W. X. Wu, X. Y. Liang and B. Y. Chen | PQ330447 | PQ338314 | N/A | N/A |  |  |
| *C. pseudoreteaudii* | CSF24547 | AA-- | Soil (*Eucalyptus* plantation) | C | 21º50'31.94"N, 108º57'37.67"E | S. F. Chen, W. X. Wu, X. Y. Liang and B. Y. Chen | PQ330448 | PQ338315 | N/A | N/A |  |  |
| *C. pseudoreteaudii* | CSF24548 | AAAA | Soil (*Eucalyptus* plantation) | C | 21º50'31.94"N, 108º57'37.67"E | S. F. Chen, W. X. Wu, X. Y. Liang and B. Y. Chen | PQ330449 | PQ338316 | PQ330766 | PQ338633 |  |  |
| *C. pseudoreteaudii* | CSF24551 | AA-- | Soil (*Eucalyptus* plantation) | C | 21º50'31.94"N, 108º57'37.67"E | S. F. Chen, W. X. Wu, X. Y. Liang and B. Y. Chen | PQ330450 | PQ338317 | N/A | N/A |  |  |
| *C. pseudoreteaudii* | CSF24552 | AA-- | Soil (*Eucalyptus* plantation) | C | 21º50'31.94"N, 108º57'37.67"E | S. F. Chen, W. X. Wu, X. Y. Liang and B. Y. Chen | PQ330451 | PQ338318 | N/A | N/A |  |  |
| *C. pseudoreteaudii* | CSF24555 | AA-- | Soil (*Eucalyptus* plantation) | C | 21º50'31.94"N, 108º57'37.67"E | S. F. Chen, W. X. Wu, X. Y. Liang and B. Y. Chen | PQ330452 | PQ338319 | N/A | N/A |  |  |
| *C. pseudoreteaudii* | CSF24558 ^e^ | AABA | 1-year-old *Eucalyptus* leaf | D | 21º50'39.81"N, 108º55'56.89"E | S. F. Chen, W. X. Wu, X. Y. Liang and B. Y. Chen | PQ330453 | PQ338320 | PQ330767 | PQ338634 |  |  |
| *C. pseudoreteaudii* | CSF24559 | AA-- | 1-year-old *Eucalyptus* leaf | D | 21º50'39.81"N, 108º55'56.89"E | S. F. Chen, W. X. Wu, X. Y. Liang and B. Y. Chen | PQ330454 | PQ338321 | N/A | N/A |  |  |
| *C. pseudoreteaudii* | CSF24560 | AA-- | 1-year-old *Eucalyptus* leaf | D | 21º50'39.81"N, 108º55'56.89"E | S. F. Chen, W. X. Wu, X. Y. Liang and B. Y. Chen | PQ330455 | PQ338322 | N/A | N/A |  |  |
| *C. pseudoreteaudii* | CSF24561 | AA-- | 1-year-old *Eucalyptus* leaf | D | 21º50'39.81"N, 108º55'56.89"E | S. F. Chen, W. X. Wu, X. Y. Liang and B. Y. Chen | PQ330456 | PQ338323 | N/A | N/A |  |  |
| *C. pseudoreteaudii* | CSF24562 | AA-- | 1-year-old *Eucalyptus* leaf | D | 21º50'39.81"N, 108º55'56.89"E | S. F. Chen, W. X. Wu, X. Y. Liang and B. Y. Chen | PQ330457 | PQ338324 | N/A | N/A |  |  |
| *C. pseudoreteaudii* | CSF24563 | AAAA | 1-year-old *Eucalyptus* leaf | D | 21º50'39.81"N, 108º55'56.89"E | S. F. Chen, W. X. Wu, X. Y. Liang and B. Y. Chen | PQ330458 | PQ338325 | PQ330768 | PQ338635 |  |  |
| *C. pseudoreteaudii* | CSF24564 | AA-- | 1-year-old *Eucalyptus* leaf | D | 21º50'39.81"N, 108º55'56.89"E | S. F. Chen, W. X. Wu, X. Y. Liang and B. Y. Chen | PQ330459 | PQ338326 | N/A | N/A |  |  |
| *C. pseudoreteaudii* | CSF24565 | AA-- | 1-year-old *Eucalyptus* leaf | D | 21º50'39.81"N, 108º55'56.89"E | S. F. Chen, W. X. Wu, X. Y. Liang and B. Y. Chen | PQ330460 | PQ338327 | N/A | N/A |  |  |
| *C. pseudoreteaudii* | CSF24566 | AA-- | 1-year-old *Eucalyptus* leaf | D | 21º50'39.81"N, 108º55'56.89"E | S. F. Chen, W. X. Wu, X. Y. Liang and B. Y. Chen | PQ330461 | PQ338328 | N/A | N/A |  |  |
| *C. pseudoreteaudii* | CSF24567 | AA-- | 1-year-old *Eucalyptus* leaf | D | 21º50'39.81"N, 108º55'56.89"E | S. F. Chen, W. X. Wu, X. Y. Liang and B. Y. Chen | PQ330462 | PQ338329 | N/A | N/A |  |  |
| *C. pseudoreteaudii* | CSF24568 | AAAA | 1-year-old *Eucalyptus* leaf | D | 21º50'39.81"N, 108º55'56.89"E | S. F. Chen, W. X. Wu, X. Y. Liang and B. Y. Chen | PQ330463 | PQ338330 | PQ330769 | PQ338636 |  |  |
| *C. pseudoreteaudii* | CSF24569 | AA-- | 1-year-old *Eucalyptus* leaf | D | 21º50'39.81"N, 108º55'56.89"E | S. F. Chen, W. X. Wu, X. Y. Liang and B. Y. Chen | PQ330464 | PQ338331 | N/A | N/A |  |  |
| *C. pseudoreteaudii* | CSF24570 | AA-- | 1-year-old *Eucalyptus* leaf | D | 21º50'39.81"N, 108º55'56.89"E | S. F. Chen, W. X. Wu, X. Y. Liang and B. Y. Chen | PQ330465 | PQ338332 | N/A | N/A |  |  |
| *C. pseudoreteaudii* | CSF24571 | AA-- | 1-year-old *Eucalyptus* leaf | D | 21º50'39.81"N, 108º55'56.89"E | S. F. Chen, W. X. Wu, X. Y. Liang and B. Y. Chen | PQ330466 | PQ338333 | N/A | N/A |  |  |
| *C. pseudoreteaudii* | CSF24572 | AA-- | 1-year-old *Eucalyptus* leaf | D | 21º50'39.81"N, 108º55'56.89"E | S. F. Chen, W. X. Wu, X. Y. Liang and B. Y. Chen | PQ330467 | PQ338334 | N/A | N/A |  |  |
| *C. pseudoreteaudii* | CSF24573 | AAAA | 1-year-old *Eucalyptus* leaf | D | 21º50'39.81"N, 108º55'56.89"E | S. F. Chen, W. X. Wu, X. Y. Liang and B. Y. Chen | PQ330468 | PQ338335 | PQ330770 | PQ338637 |  |  |
| *C. pseudoreteaudii* | CSF24574 | AA-- | 1-year-old *Eucalyptus* leaf | D | 21º50'39.81"N, 108º55'56.89"E | S. F. Chen, W. X. Wu, X. Y. Liang and B. Y. Chen | PQ330469 | PQ338336 | N/A | N/A |  |  |
| *C. pseudoreteaudii* | CSF24575 | AA-- | 1-year-old *Eucalyptus* leaf | D | 21º50'39.81"N, 108º55'56.89"E | S. F. Chen, W. X. Wu, X. Y. Liang and B. Y. Chen | PQ330470 | PQ338337 | N/A | N/A |  |  |
| *C. pseudoreteaudii* | CSF24576 | AA-- | 1-year-old *Eucalyptus* leaf | D | 21º50'39.81"N, 108º55'56.89"E | S. F. Chen, W. X. Wu, X. Y. Liang and B. Y. Chen | PQ330471 | PQ338338 | N/A | N/A |  |  |
| *C. pseudoreteaudii* | CSF24577 | AA-- | 1-year-old *Eucalyptus* leaf | D | 21º50'39.81"N, 108º55'56.89"E | S. F. Chen, W. X. Wu, X. Y. Liang and B. Y. Chen | PQ330472 | PQ338339 | N/A | N/A |  |  |
| *C. pseudoreteaudii* | CSF24578 | AAAA | 1-year-old *Eucalyptus* leaf | D | 21º50'39.81"N, 108º55'56.89"E | S. F. Chen, W. X. Wu, X. Y. Liang and B. Y. Chen | PQ330473 | PQ338340 | PQ330771 | PQ338638 |  |  |
| *C. pseudoreteaudii* | CSF24579 | AA-- | 1-year-old *Eucalyptus* leaf | D | 21º50'39.81"N, 108º55'56.89"E | S. F. Chen, W. X. Wu, X. Y. Liang and B. Y. Chen | PQ330474 | PQ338341 | N/A | N/A |  |  |
| *C. pseudoreteaudii* | CSF24580 | AA-- | 1-year-old *Eucalyptus* leaf | D | 21º50'39.81"N, 108º55'56.89"E | S. F. Chen, W. X. Wu, X. Y. Liang and B. Y. Chen | PQ330475 | PQ338342 | N/A | N/A |  |  |
| *C. pseudoreteaudii* | CSF24581 | AA-- | 1-year-old *Eucalyptus* leaf | D | 21º50'39.81"N, 108º55'56.89"E | S. F. Chen, W. X. Wu, X. Y. Liang and B. Y. Chen | PQ330476 | PQ338343 | N/A | N/A |  |  |
| *C. pseudoreteaudii* | CSF24582 | AA-- | 1-year-old *Eucalyptus* leaf | D | 21º50'39.81"N, 108º55'56.89"E | S. F. Chen, W. X. Wu, X. Y. Liang and B. Y. Chen | PQ330477 | PQ338344 | N/A | N/A |  |  |
| *C. pseudoreteaudii* | CSF24583 | AAAA | 1-year-old *Eucalyptus* leaf | D | 21º50'39.81"N, 108º55'56.89"E | S. F. Chen, W. X. Wu, X. Y. Liang and B. Y. Chen | PQ330478 | PQ338345 | PQ330772 | PQ338639 |  |  |
| *C. pseudoreteaudii* | CSF24584 | AA-- | 1-year-old *Eucalyptus* leaf | D | 21º50'39.81"N, 108º55'56.89"E | S. F. Chen, W. X. Wu, X. Y. Liang and B. Y. Chen | PQ330479 | PQ338346 | N/A | N/A |  |  |
| *C. pseudoreteaudii* | CSF24585 | AA-- | 1-year-old *Eucalyptus* leaf | D | 21º50'39.81"N, 108º55'56.89"E | S. F. Chen, W. X. Wu, X. Y. Liang and B. Y. Chen | PQ330480 | PQ338347 | N/A | N/A |  |  |
| *C. pseudoreteaudii* | CSF24586 | AA-- | 1-year-old *Eucalyptus* leaf | D | 21º50'39.81"N, 108º55'56.89"E | S. F. Chen, W. X. Wu, X. Y. Liang and B. Y. Chen | PQ330481 | PQ338348 | N/A | N/A |  |  |
| *C. pseudoreteaudii* | CSF24587 | AA-- | 1-year-old *Eucalyptus* leaf | D | 21º50'39.81"N, 108º55'56.89"E | S. F. Chen, W. X. Wu, X. Y. Liang and B. Y. Chen | PQ330482 | PQ338349 | N/A | N/A |  |  |
| *C. pseudoreteaudii* | CSF24588 | AAAA | 1-year-old *Eucalyptus* leaf | D | 21º50'39.81"N, 108º55'56.89"E | S. F. Chen, W. X. Wu, X. Y. Liang and B. Y. Chen | PQ330483 | PQ338350 | PQ330773 | PQ338640 |  |  |
| *C. pseudoreteaudii* | CSF24589 | AA-- | 1-year-old *Eucalyptus* leaf | D | 21º50'39.81"N, 108º55'56.89"E | S. F. Chen, W. X. Wu, X. Y. Liang and B. Y. Chen | PQ330484 | PQ338351 | N/A | N/A |  |  |
| *C. pseudoreteaudii* | CSF24590 | AA-- | 1-year-old *Eucalyptus* leaf | D | 21º50'39.81"N, 108º55'56.89"E | S. F. Chen, W. X. Wu, X. Y. Liang and B. Y. Chen | PQ330485 | PQ338352 | N/A | N/A |  |  |
| *C. pseudoreteaudii* | CSF24591 | AA-- | 1-year-old *Eucalyptus* leaf | D | 21º50'39.81"N, 108º55'56.89"E | S. F. Chen, W. X. Wu, X. Y. Liang and B. Y. Chen | PQ330486 | PQ338353 | N/A | N/A |  |  |
| *C. pseudoreteaudii* | CSF24592 | AA-- | 1-year-old *Eucalyptus* leaf | D | 21º50'39.81"N, 108º55'56.89"E | S. F. Chen, W. X. Wu, X. Y. Liang and B. Y. Chen | PQ330487 | PQ338354 | N/A | N/A |  |  |
| *C. pseudoreteaudii* | CSF24593 | AABA | 1-year-old *Eucalyptus* leaf | D | 21º50'39.81"N, 108º55'56.89"E | S. F. Chen, W. X. Wu, X. Y. Liang and B. Y. Chen | PQ330488 | PQ338355 | PQ330774 | PQ338641 |  |  |
| *C. pseudoreteaudii* | CSF24594 | AA-- | 1-year-old *Eucalyptus* leaf | D | 21º50'39.81"N, 108º55'56.89"E | S. F. Chen, W. X. Wu, X. Y. Liang and B. Y. Chen | PQ330489 | PQ338356 | N/A | N/A |  |  |
| *C. pseudoreteaudii* | CSF24595 | AA-- | 1-year-old *Eucalyptus* leaf | D | 21º50'39.81"N, 108º55'56.89"E | S. F. Chen, W. X. Wu, X. Y. Liang and B. Y. Chen | PQ330490 | PQ338357 | N/A | N/A |  |  |
| *C. pseudoreteaudii* | CSF24596 | AA-- | 1-year-old *Eucalyptus* leaf | D | 21º50'39.81"N, 108º55'56.89"E | S. F. Chen, W. X. Wu, X. Y. Liang and B. Y. Chen | PQ330491 | PQ338358 | N/A | N/A |  |  |
| *C. pseudoreteaudii* | CSF24597 | AABA | 1-year-old *Eucalyptus* leaf | D | 21º50'39.81"N, 108º55'56.89"E | S. F. Chen, W. X. Wu, X. Y. Liang and B. Y. Chen | PQ330492 | PQ338359 | PQ330775 | PQ338642 |  |  |
| *C. pseudoreteaudii* | CSF24598 | AA-- | 1-year-old *Eucalyptus* leaf | D | 21º50'39.81"N, 108º55'56.89"E | S. F. Chen, W. X. Wu, X. Y. Liang and B. Y. Chen | PQ330493 | PQ338360 | N/A | N/A |  |  |
| *C. pseudoreteaudii* | CSF24599 | AA-- | 1-year-old *Eucalyptus* leaf | D | 21º50'39.81"N, 108º55'56.89"E | S. F. Chen, W. X. Wu, X. Y. Liang and B. Y. Chen | PQ330494 | PQ338361 | N/A | N/A |  |  |
| *C. pseudoreteaudii* | CSF24600 | AA-- | 1-year-old *Eucalyptus* leaf | D | 21º50'39.81"N, 108º55'56.89"E | S. F. Chen, W. X. Wu, X. Y. Liang and B. Y. Chen | PQ330495 | PQ338362 | N/A | N/A |  |  |
| *C. pseudoreteaudii* | CSF24601 | AA-- | 1-year-old *Eucalyptus* leaf | D | 21º50'39.81"N, 108º55'56.89"E | S. F. Chen, W. X. Wu, X. Y. Liang and B. Y. Chen | PQ330496 | PQ338363 | N/A | N/A |  |  |
| *C. pseudoreteaudii* | CSF24602 | AABA | 1-year-old *Eucalyptus* leaf | D | 21º50'39.81"N, 108º55'56.89"E | S. F. Chen, W. X. Wu, X. Y. Liang and B. Y. Chen | PQ330497 | PQ338364 | PQ330776 | PQ338643 |  |  |
| *C. pseudoreteaudii* | CSF24603 | AA-- | 1-year-old *Eucalyptus* leaf | D | 21º50'39.81"N, 108º55'56.89"E | S. F. Chen, W. X. Wu, X. Y. Liang and B. Y. Chen | PQ330498 | PQ338365 | N/A | N/A |  |  |
| *C. pseudoreteaudii* | CSF24604 | AA-- | 1-year-old *Eucalyptus* leaf | D | 21º50'39.81"N, 108º55'56.89"E | S. F. Chen, W. X. Wu, X. Y. Liang and B. Y. Chen | PQ330499 | PQ338366 | N/A | N/A |  |  |
| *C. pseudoreteaudii* | CSF24605 | AA-- | 1-year-old *Eucalyptus* leaf | D | 21º50'39.81"N, 108º55'56.89"E | S. F. Chen, W. X. Wu, X. Y. Liang and B. Y. Chen | PQ330500 | PQ338367 | N/A | N/A |  |  |
| *C. pseudoreteaudii* | CSF24606 | AA-- | 1-year-old *Eucalyptus* leaf | D | 21º50'39.81"N, 108º55'56.89"E | S. F. Chen, W. X. Wu, X. Y. Liang and B. Y. Chen | PQ330501 | PQ338368 | N/A | N/A |  |  |
| *C. pseudoreteaudii* | CSF24607 | AA-- | 1-year-old *Eucalyptus* leaf | D | 21º50'39.81"N, 108º55'56.89"E | S. F. Chen, W. X. Wu, X. Y. Liang and B. Y. Chen | PQ330502 | PQ338369 | N/A | N/A |  |  |
| *C. pseudoreteaudii* | CSF24608 | AABA | Soil (*Eucalyptus* plantation) | D | 21º50'39.81"N, 108º55'56.89"E | S. F. Chen, W. X. Wu, X. Y. Liang and B. Y. Chen | PQ330503 | PQ338370 | PQ330777 | PQ338644 |  |  |
| *C. pseudoreteaudii* | CSF24609 | AA-- | Soil (*Eucalyptus* plantation) | D | 21º50'39.81"N, 108º55'56.89"E | S. F. Chen, W. X. Wu, X. Y. Liang and B. Y. Chen | PQ330504 | PQ338371 | N/A | N/A |  |  |
| *C. pseudoreteaudii* | CSF24610 | AA-- | Soil (*Eucalyptus* plantation) | D | 21º50'39.81"N, 108º55'56.89"E | S. F. Chen, W. X. Wu, X. Y. Liang and B. Y. Chen | PQ330505 | PQ338372 | N/A | N/A |  |  |
| *C. pseudoreteaudii* | CSF24612 | AA-- | Soil (*Eucalyptus* plantation) | D | 21º50'39.81"N, 108º55'56.89"E | S. F. Chen, W. X. Wu, X. Y. Liang and B. Y. Chen | PQ330506 | PQ338373 | N/A | N/A |  |  |
| *C. pseudoreteaudii* | CSF24614 | AA-- | Soil (*Eucalyptus* plantation) | D | 21º50'39.81"N, 108º55'56.89"E | S. F. Chen, W. X. Wu, X. Y. Liang and B. Y. Chen | PQ330507 | PQ338374 | N/A | N/A |  |  |
| *C. pseudoreteaudii* | CSF24615 | AA-- | Soil (*Eucalyptus* plantation) | D | 21º50'39.81"N, 108º55'56.89"E | S. F. Chen, W. X. Wu, X. Y. Liang and B. Y. Chen | PQ330508 | PQ338375 | N/A | N/A |  |  |
| *C. pseudoreteaudii* | CSF24617 | AABA | Soil (*Eucalyptus* plantation) | D | 21º50'39.81"N, 108º55'56.89"E | S. F. Chen, W. X. Wu, X. Y. Liang and B. Y. Chen | PQ330509 | PQ338376 | PQ330778 | PQ338645 |  |  |
| *C. pseudoreteaudii* | CSF24619 | AA-- | Soil (*Eucalyptus* plantation) | D | 21º50'39.81"N, 108º55'56.89"E | S. F. Chen, W. X. Wu, X. Y. Liang and B. Y. Chen | PQ330510 | PQ338377 | N/A | N/A |  |  |
| *C. pseudoreteaudii* | CSF24621 | AA-- | Soil (*Eucalyptus* plantation) | D | 21º50'39.81"N, 108º55'56.89"E | S. F. Chen, W. X. Wu, X. Y. Liang and B. Y. Chen | PQ330511 | PQ338378 | N/A | N/A |  |  |
| *C. pseudoreteaudii* | CSF24622 | AA-- | Soil (*Eucalyptus* plantation) | D | 21º50'39.81"N, 108º55'56.89"E | S. F. Chen, W. X. Wu, X. Y. Liang and B. Y. Chen | PQ330512 | PQ338379 | N/A | N/A |  |  |
| *C. pseudoreteaudii* | CSF24624 | AA-- | Soil (*Eucalyptus* plantation) | D | 21º50'39.81"N, 108º55'56.89"E | S. F. Chen, W. X. Wu, X. Y. Liang and B. Y. Chen | PQ330513 | PQ338380 | N/A | N/A |  |  |
| *C. pseudoreteaudii* | CSF24625 | AA-- | Soil (*Eucalyptus* plantation) | D | 21º50'39.81"N, 108º55'56.89"E | S. F. Chen, W. X. Wu, X. Y. Liang and B. Y. Chen | PQ330514 | PQ338381 | N/A | N/A |  |  |
| *C. pseudoreteaudii* | CSF24626 | AABA | Soil (*Eucalyptus* plantation) | D | 21º50'39.81"N, 108º55'56.89"E | S. F. Chen, W. X. Wu, X. Y. Liang and B. Y. Chen | PQ330515 | PQ338382 | PQ330779 | PQ338646 |  |  |
| *C. pseudoreteaudii* | CSF24627 | AA-- | Soil (*Eucalyptus* plantation) | D | 21º50'39.81"N, 108º55'56.89"E | S. F. Chen, W. X. Wu, X. Y. Liang and B. Y. Chen | PQ330516 | PQ338383 | N/A | N/A |  |  |
| *C. pseudoreteaudii* | CSF24629 | AA-- | Soil (*Eucalyptus* plantation) | D | 21º50'39.81"N, 108º55'56.89"E | S. F. Chen, W. X. Wu, X. Y. Liang and B. Y. Chen | PQ330517 | PQ338384 | N/A | N/A |  |  |
| *C. pseudoreteaudii* | CSF24631 | AA-- | Soil (*Eucalyptus* plantation) | D | 21º50'39.81"N, 108º55'56.89"E | S. F. Chen, W. X. Wu, X. Y. Liang and B. Y. Chen | PQ330518 | PQ338385 | N/A | N/A |  |  |
| *C. pseudoreteaudii* | CSF24632 | AABA | Soil (*Eucalyptus* plantation) | D | 21º50'39.81"N, 108º55'56.89"E | S. F. Chen, W. X. Wu, X. Y. Liang and B. Y. Chen | PQ330519 | PQ338386 | PQ330780 | PQ338647 |  |  |
| *C. pseudoreteaudii* | CSF24634 | AA-- | Soil (*Eucalyptus* plantation) | D | 21º50'39.81"N, 108º55'56.89"E | S. F. Chen, W. X. Wu, X. Y. Liang and B. Y. Chen | PQ330520 | PQ338387 | N/A | N/A |  |  |
| *C. pseudoreteaudii* | CSF24636 | AA-- | Soil (*Eucalyptus* plantation) | D | 21º50'39.81"N, 108º55'56.89"E | S. F. Chen, W. X. Wu, X. Y. Liang and B. Y. Chen | PQ330521 | PQ338388 | N/A | N/A |  |  |
| *C. pseudoreteaudii* | CSF24637 | AA-- | Soil (*Eucalyptus* plantation) | D | 21º50'39.81"N, 108º55'56.89"E | S. F. Chen, W. X. Wu, X. Y. Liang and B. Y. Chen | PQ330522 | PQ338389 | N/A | N/A |  |  |
| *C. pseudoreteaudii* | CSF24639 | AA-- | Soil (*Eucalyptus* plantation) | D | 21º50'39.81"N, 108º55'56.89"E | S. F. Chen, W. X. Wu, X. Y. Liang and B. Y. Chen | PQ330523 | PQ338390 | N/A | N/A |  |  |
| *C. pseudoreteaudii* | CSF24641 | AA-- | Soil (*Eucalyptus* plantation) | D | 21º50'39.81"N, 108º55'56.89"E | S. F. Chen, W. X. Wu, X. Y. Liang and B. Y. Chen | PQ330524 | PQ338391 | N/A | N/A |  |  |
| *C. pseudoreteaudii* | CSF24642 | AABA | Soil (*Eucalyptus* plantation) | D | 21º50'39.81"N, 108º55'56.89"E | S. F. Chen, W. X. Wu, X. Y. Liang and B. Y. Chen | PQ330525 | PQ338392 | PQ330781 | PQ338648 |  |  |
| *C. pseudoreteaudii* | CSF24643 | AA-- | Soil (*Eucalyptus* plantation) | D | 21º50'39.81"N, 108º55'56.89"E | S. F. Chen, W. X. Wu, X. Y. Liang and B. Y. Chen | PQ330526 | PQ338393 | N/A | N/A |  |  |
| *C. pseudoreteaudii* | CSF24644 | AA-- | Soil (*Eucalyptus* plantation) | D | 21º50'39.81"N, 108º55'56.89"E | S. F. Chen, W. X. Wu, X. Y. Liang and B. Y. Chen | PQ330527 | PQ338394 | N/A | N/A |  |  |
| *C. pseudoreteaudii* | CSF24645 | AA-- | Soil (*Eucalyptus* plantation) | D | 21º50'39.81"N, 108º55'56.89"E | S. F. Chen, W. X. Wu, X. Y. Liang and B. Y. Chen | PQ330528 | PQ338395 | N/A | N/A |  |  |
| *C. pseudoreteaudii* | CSF24646 | AA-- | Soil (*Eucalyptus* plantation) | D | 21º50'39.81"N, 108º55'56.89"E | S. F. Chen, W. X. Wu, X. Y. Liang and B. Y. Chen | PQ330529 | PQ338396 | N/A | N/A |  |  |
| *C. pseudoreteaudii* | CSF24649 | AA-- | Soil (*Eucalyptus* plantation) | D | 21º50'39.81"N, 108º55'56.89"E | S. F. Chen, W. X. Wu, X. Y. Liang and B. Y. Chen | PQ330530 | PQ338397 | N/A | N/A |  |  |
| *C. pseudoreteaudii* | CSF24650 | AAAA | Soil (*Eucalyptus* plantation) | D | 21º50'39.81"N, 108º55'56.89"E | S. F. Chen, W. X. Wu, X. Y. Liang and B. Y. Chen | PQ330531 | PQ338398 | PQ330782 | PQ338649 |  |  |
| *C. pseudoreteaudii* | CSF24745 | AAAA | 1-year-old *Eucalyptus* leaf | E | 21º8'34.99"N, 110º5'32.03"E | S. F. Chen, W. X. Wu, X. Y. Liang and B. Y. Chen | PQ330532 | PQ338399 | PQ330783 | PQ338650 |  |  |
| *C. pseudoreteaudii* | CSF24746 | AA-- | 1-year-old *Eucalyptus* leaf | E | 21º8'34.99"N, 110º5'32.03"E | S. F. Chen, W. X. Wu, X. Y. Liang and B. Y. Chen | PQ330533 | PQ338400 | N/A | N/A |  |  |
| *C. pseudoreteaudii* | CSF24747 | AA-- | 1-year-old *Eucalyptus* leaf | E | 21º8'34.99"N, 110º5'32.03"E | S. F. Chen, W. X. Wu, X. Y. Liang and B. Y. Chen | PQ330534 | PQ338401 | N/A | N/A |  |  |
| *C. pseudoreteaudii* | CSF24748 | AA-- | 1-year-old *Eucalyptus* leaf | E | 21º8'34.99"N, 110º5'32.03"E | S. F. Chen, W. X. Wu, X. Y. Liang and B. Y. Chen | PQ330535 | PQ338402 | N/A | N/A |  |  |
| *C. pseudoreteaudii* | CSF24749 | AA-- | 1-year-old *Eucalyptus* leaf | E | 21º8'34.99"N, 110º5'32.03"E | S. F. Chen, W. X. Wu, X. Y. Liang and B. Y. Chen | PQ330536 | PQ338403 | N/A | N/A |  |  |
| *C. pseudoreteaudii* | CSF24750 | AAAA | 1-year-old *Eucalyptus* leaf | E | 21º8'34.99"N, 110º5'32.03"E | S. F. Chen, W. X. Wu, X. Y. Liang and B. Y. Chen | PQ330537 | PQ338404 | PQ330784 | PQ338651 |  |  |
| *C. pseudoreteaudii* | CSF24751 | AA-- | 1-year-old *Eucalyptus* leaf | E | 21º8'34.99"N, 110º5'32.03"E | S. F. Chen, W. X. Wu, X. Y. Liang and B. Y. Chen | PQ330538 | PQ338405 | N/A | N/A |  |  |
| *C. pseudoreteaudii* | CSF24752 | AA-- | 1-year-old *Eucalyptus* leaf | E | 21º8'34.99"N, 110º5'32.03"E | S. F. Chen, W. X. Wu, X. Y. Liang and B. Y. Chen | PQ330539 | PQ338406 | N/A | N/A |  |  |
| *C. pseudoreteaudii* | CSF24753 | AA-- | 1-year-old *Eucalyptus* leaf | E | 21º8'34.99"N, 110º5'32.03"E | S. F. Chen, W. X. Wu, X. Y. Liang and B. Y. Chen | PQ330540 | PQ338407 | N/A | N/A |  |  |
| *C. pseudoreteaudii* | CSF24754 | AA-- | 1-year-old *Eucalyptus* leaf | E | 21º8'34.99"N, 110º5'32.03"E | S. F. Chen, W. X. Wu, X. Y. Liang and B. Y. Chen | PQ330541 | PQ338408 | N/A | N/A |  |  |
| *C. pseudoreteaudii* | CSF24755 | AAAA | 1-year-old *Eucalyptus* leaf | E | 21º8'34.99"N, 110º5'32.03"E | S. F. Chen, W. X. Wu, X. Y. Liang and B. Y. Chen | PQ330542 | PQ338409 | PQ330785 | PQ338652 |  |  |
| *C. pseudoreteaudii* | CSF24756 | AA-- | 1-year-old *Eucalyptus* leaf | E | 21º8'34.99"N, 110º5'32.03"E | S. F. Chen, W. X. Wu, X. Y. Liang and B. Y. Chen | PQ330543 | PQ338410 | N/A | N/A |  |  |
| *C. pseudoreteaudii* | CSF24757 | AA-- | 1-year-old *Eucalyptus* leaf | E | 21º8'34.99"N, 110º5'32.03"E | S. F. Chen, W. X. Wu, X. Y. Liang and B. Y. Chen | PQ330544 | PQ338411 | N/A | N/A |  |  |
| *C. pseudoreteaudii* | CSF24758 | AA-- | 1-year-old *Eucalyptus* leaf | E | 21º8'34.99"N, 110º5'32.03"E | S. F. Chen, W. X. Wu, X. Y. Liang and B. Y. Chen | PQ330545 | PQ338412 | N/A | N/A |  |  |
| *C. pseudoreteaudii* | CSF24759 | AA-- | 1-year-old *Eucalyptus* leaf | E | 21º8'34.99"N, 110º5'32.03"E | S. F. Chen, W. X. Wu, X. Y. Liang and B. Y. Chen | PQ330546 | PQ338413 | N/A | N/A |  |  |
| *C. pseudoreteaudii* | CSF24760 | AAAA | 1-year-old *Eucalyptus* leaf | E | 21º8'34.99"N, 110º5'32.03"E | S. F. Chen, W. X. Wu, X. Y. Liang and B. Y. Chen | PQ330547 | PQ338414 | PQ330786 | PQ338653 |  |  |
| *C. pseudoreteaudii* | CSF24761 | AA-- | 1-year-old *Eucalyptus* leaf | E | 21º8'34.99"N, 110º5'32.03"E | S. F. Chen, W. X. Wu, X. Y. Liang and B. Y. Chen | PQ330548 | PQ338415 | N/A | N/A |  |  |
| *C. pseudoreteaudii* | CSF24762 | AA-- | 1-year-old *Eucalyptus* leaf | E | 21º8'34.99"N, 110º5'32.03"E | S. F. Chen, W. X. Wu, X. Y. Liang and B. Y. Chen | PQ330549 | PQ338416 | N/A | N/A |  |  |
| *C. pseudoreteaudii* | CSF24763 | AA-- | 1-year-old *Eucalyptus* leaf | E | 21º8'34.99"N, 110º5'32.03"E | S. F. Chen, W. X. Wu, X. Y. Liang and B. Y. Chen | PQ330550 | PQ338417 | N/A | N/A |  |  |
| *C. pseudoreteaudii* | CSF24764 | AA-- | 1-year-old *Eucalyptus* leaf | E | 21º8'34.99"N, 110º5'32.03"E | S. F. Chen, W. X. Wu, X. Y. Liang and B. Y. Chen | PQ330551 | PQ338418 | N/A | N/A |  |  |
| *C. pseudoreteaudii* | CSF24765 | AAAA | 1-year-old *Eucalyptus* leaf | E | 21º8'34.99"N, 110º5'32.03"E | S. F. Chen, W. X. Wu, X. Y. Liang and B. Y. Chen | PQ330552 | PQ338419 | PQ330787 | PQ338654 |  |  |
| *C. pseudoreteaudii* | CSF24766 | AA-- | 1-year-old *Eucalyptus* leaf | E | 21º8'34.99"N, 110º5'32.03"E | S. F. Chen, W. X. Wu, X. Y. Liang and B. Y. Chen | PQ330553 | PQ338420 | N/A | N/A |  |  |
| *C. pseudoreteaudii* | CSF24767 | AA-- | 1-year-old *Eucalyptus* leaf | E | 21º8'34.99"N, 110º5'32.03"E | S. F. Chen, W. X. Wu, X. Y. Liang and B. Y. Chen | PQ330554 | PQ338421 | N/A | N/A |  |  |
| *C. pseudoreteaudii* | CSF24768 | AA-- | 1-year-old *Eucalyptus* leaf | E | 21º8'34.99"N, 110º5'32.03"E | S. F. Chen, W. X. Wu, X. Y. Liang and B. Y. Chen | PQ330555 | PQ338422 | N/A | N/A |  |  |
| *C. pseudoreteaudii* | CSF24769 | AA-- | 1-year-old *Eucalyptus* leaf | E | 21º8'34.99"N, 110º5'32.03"E | S. F. Chen, W. X. Wu, X. Y. Liang and B. Y. Chen | PQ330556 | PQ338423 | N/A | N/A |  |  |
| *C. pseudoreteaudii* | CSF24770 | AAAA | 1-year-old *Eucalyptus* leaf | E | 21º8'34.99"N, 110º5'32.03"E | S. F. Chen, W. X. Wu, X. Y. Liang and B. Y. Chen | PQ330557 | PQ338424 | PQ330788 | PQ338655 |  |  |
| *C. pseudoreteaudii* | CSF24771 | AA-- | 1-year-old *Eucalyptus* leaf | E | 21º8'34.99"N, 110º5'32.03"E | S. F. Chen, W. X. Wu, X. Y. Liang and B. Y. Chen | PQ330558 | PQ338425 | N/A | N/A |  |  |
| *C. pseudoreteaudii* | CSF24772 | AA-- | 1-year-old *Eucalyptus* leaf | E | 21º8'34.99"N, 110º5'32.03"E | S. F. Chen, W. X. Wu, X. Y. Liang and B. Y. Chen | PQ330559 | PQ338426 | N/A | N/A |  |  |
| *C. pseudoreteaudii* | CSF24773 | AA-- | 1-year-old *Eucalyptus* leaf | E | 21º8'34.99"N, 110º5'32.03"E | S. F. Chen, W. X. Wu, X. Y. Liang and B. Y. Chen | PQ330560 | PQ338427 | N/A | N/A |  |  |
| *C. pseudoreteaudii* | CSF24774 | AA-- | 1-year-old *Eucalyptus* leaf | E | 21º8'34.99"N, 110º5'32.03"E | S. F. Chen, W. X. Wu, X. Y. Liang and B. Y. Chen | PQ330561 | PQ338428 | N/A | N/A |  |  |
| *C. pseudoreteaudii* | CSF24775 | AAAA | 1-year-old *Eucalyptus* leaf | E | 21º8'34.99"N, 110º5'32.03"E | S. F. Chen, W. X. Wu, X. Y. Liang and B. Y. Chen | PQ330562 | PQ338429 | PQ330789 | PQ338656 |  |  |
| *C. pseudoreteaudii* | CSF24776 | AA-- | 1-year-old *Eucalyptus* leaf | E | 21º8'34.99"N, 110º5'32.03"E | S. F. Chen, W. X. Wu, X. Y. Liang and B. Y. Chen | PQ330563 | PQ338430 | N/A | N/A |  |  |
| *C. pseudoreteaudii* | CSF24777 | AA-- | 1-year-old *Eucalyptus* leaf | E | 21º8'34.99"N, 110º5'32.03"E | S. F. Chen, W. X. Wu, X. Y. Liang and B. Y. Chen | PQ330564 | PQ338431 | N/A | N/A |  |  |
| *C. pseudoreteaudii* | CSF24778 | AA-- | 1-year-old *Eucalyptus* leaf | E | 21º8'34.99"N, 110º5'32.03"E | S. F. Chen, W. X. Wu, X. Y. Liang and B. Y. Chen | PQ330565 | PQ338432 | N/A | N/A |  |  |
| *C. pseudoreteaudii* | CSF24779 | AA-- | 1-year-old *Eucalyptus* leaf | E | 21º8'34.99"N, 110º5'32.03"E | S. F. Chen, W. X. Wu, X. Y. Liang and B. Y. Chen | PQ330566 | PQ338433 | N/A | N/A |  |  |
| *C. pseudoreteaudii* | CSF24780 | AAAA | 1-year-old *Eucalyptus* leaf | E | 21º8'34.99"N, 110º5'32.03"E | S. F. Chen, W. X. Wu, X. Y. Liang and B. Y. Chen | PQ330567 | PQ338434 | PQ330790 | PQ338657 |  |  |
| *C. pseudoreteaudii* | CSF24781 | AA-- | 1-year-old *Eucalyptus* leaf | E | 21º8'34.99"N, 110º5'32.03"E | S. F. Chen, W. X. Wu, X. Y. Liang and B. Y. Chen | PQ330568 | PQ338435 | N/A | N/A |  |  |
| *C. pseudoreteaudii* | CSF24782 | AA-- | 1-year-old *Eucalyptus* leaf | E | 21º8'34.99"N, 110º5'32.03"E | S. F. Chen, W. X. Wu, X. Y. Liang and B. Y. Chen | PQ330569 | PQ338436 | N/A | N/A |  |  |
| *C. pseudoreteaudii* | CSF24783 | AA-- | 1-year-old *Eucalyptus* leaf | E | 21º8'34.99"N, 110º5'32.03"E | S. F. Chen, W. X. Wu, X. Y. Liang and B. Y. Chen | PQ330570 | PQ338437 | N/A | N/A |  |  |
| *C. pseudoreteaudii* | CSF24784 | AA-- | 1-year-old *Eucalyptus* leaf | E | 21º8'34.99"N, 110º5'32.03"E | S. F. Chen, W. X. Wu, X. Y. Liang and B. Y. Chen | PQ330571 | PQ338438 | N/A | N/A |  |  |
| *C. pseudoreteaudii* | CSF24785 | AAAA | 1-year-old *Eucalyptus* leaf | E | 21º8'34.99"N, 110º5'32.03"E | S. F. Chen, W. X. Wu, X. Y. Liang and B. Y. Chen | PQ330572 | PQ338439 | PQ330791 | PQ338658 |  |  |
| *C. pseudoreteaudii* | CSF24786 | AA-- | 1-year-old *Eucalyptus* leaf | E | 21º8'34.99"N, 110º5'32.03"E | S. F. Chen, W. X. Wu, X. Y. Liang and B. Y. Chen | PQ330573 | PQ338440 | N/A | N/A |  |  |
| *C. pseudoreteaudii* | CSF24787 | AA-- | 1-year-old *Eucalyptus* leaf | E | 21º8'34.99"N, 110º5'32.03"E | S. F. Chen, W. X. Wu, X. Y. Liang and B. Y. Chen | PQ330574 | PQ338441 | N/A | N/A |  |  |
| *C. pseudoreteaudii* | CSF24788 | AA-- | 1-year-old *Eucalyptus* leaf | E | 21º8'34.99"N, 110º5'32.03"E | S. F. Chen, W. X. Wu, X. Y. Liang and B. Y. Chen | PQ330575 | PQ338442 | N/A | N/A |  |  |
| *C. pseudoreteaudii* | CSF24789 | AA-- | 1-year-old *Eucalyptus* leaf | E | 21º8'34.99"N, 110º5'32.03"E | S. F. Chen, W. X. Wu, X. Y. Liang and B. Y. Chen | PQ330576 | PQ338443 | N/A | N/A |  |  |
| *C. pseudoreteaudii* | CSF24790 | AA-- | 1-year-old *Eucalyptus* leaf | E | 21º8'34.99"N, 110º5'32.03"E | S. F. Chen, W. X. Wu, X. Y. Liang and B. Y. Chen | PQ330577 | PQ338444 | N/A | N/A |  |  |
| *C. pseudoreteaudii* | CSF24791 | AAAA | 1-year-old *Eucalyptus* leaf | E | 21º8'34.99"N, 110º5'32.03"E | S. F. Chen, W. X. Wu, X. Y. Liang and B. Y. Chen | PQ330578 | PQ338445 | PQ330792 | PQ338659 |  |  |
| *C. pseudoreteaudii* | CSF24792 | AA-- | 1-year-old *Eucalyptus* leaf | E | 21º8'34.99"N, 110º5'32.03"E | S. F. Chen, W. X. Wu, X. Y. Liang and B. Y. Chen | PQ330579 | PQ338446 | N/A | N/A |  |  |
| *C. pseudoreteaudii* | CSF24793 | AA-- | 1-year-old *Eucalyptus* leaf | E | 21º8'34.99"N, 110º5'32.03"E | S. F. Chen, W. X. Wu, X. Y. Liang and B. Y. Chen | PQ330580 | PQ338447 | N/A | N/A |  |  |
| *C. pseudoreteaudii* | CSF24794 | AA-- | 1-year-old *Eucalyptus* leaf | E | 21º8'34.99"N, 110º5'32.03"E | S. F. Chen, W. X. Wu, X. Y. Liang and B. Y. Chen | PQ330581 | PQ338448 | N/A | N/A |  |  |
| *C. pseudoreteaudii* | CSF24795 | AAAA | Soil (*Eucalyptus* plantation) | E | 21º8'34.99"N, 110º5'32.03"E | S. F. Chen, W. X. Wu, X. Y. Liang and B. Y. Chen | PQ330582 | PQ338449 | PQ330793 | PQ338660 |  |  |
| *C. pseudoreteaudii* | CSF24796 | AA-- | Soil (*Eucalyptus* plantation) | E | 21º8'34.99"N, 110º5'32.03"E | S. F. Chen, W. X. Wu, X. Y. Liang and B. Y. Chen | PQ330583 | PQ338450 | N/A | N/A |  |  |
| *C. pseudoreteaudii* | CSF24797 | AA-- | Soil (*Eucalyptus* plantation) | E | 21º8'34.99"N, 110º5'32.03"E | S. F. Chen, W. X. Wu, X. Y. Liang and B. Y. Chen | PQ330584 | PQ338451 | N/A | N/A |  |  |
| *C. pseudoreteaudii* | CSF24798 | AA-- | Soil (*Eucalyptus* plantation) | E | 21º8'34.99"N, 110º5'32.03"E | S. F. Chen, W. X. Wu, X. Y. Liang and B. Y. Chen | PQ330585 | PQ338452 | N/A | N/A |  |  |
| *C. pseudoreteaudii* | CSF24799 | AA-- | Soil (*Eucalyptus* plantation) | E | 21º8'34.99"N, 110º5'32.03"E | S. F. Chen, W. X. Wu, X. Y. Liang and B. Y. Chen | PQ330586 | PQ338453 | N/A | N/A |  |  |
| *C. pseudoreteaudii* | CSF24800 | AAAA | Soil (*Eucalyptus* plantation) | E | 21º8'34.99"N, 110º5'32.03"E | S. F. Chen, W. X. Wu, X. Y. Liang and B. Y. Chen | PQ330587 | PQ338454 | PQ330794 | PQ338661 |  |  |
| *C. pseudoreteaudii* | CSF24801 | AA-- | Soil (*Eucalyptus* plantation) | E | 21º8'34.99"N, 110º5'32.03"E | S. F. Chen, W. X. Wu, X. Y. Liang and B. Y. Chen | PQ330588 | PQ338455 | N/A | N/A |  |  |
| *C. pseudoreteaudii* | CSF24802 | AA-- | Soil (*Eucalyptus* plantation) | E | 21º8'34.99"N, 110º5'32.03"E | S. F. Chen, W. X. Wu, X. Y. Liang and B. Y. Chen | PQ330589 | PQ338456 | N/A | N/A |  |  |
| *C. pseudoreteaudii* | CSF24803 | AA-- | Soil (*Eucalyptus* plantation) | E | 21º8'34.99"N, 110º5'32.03"E | S. F. Chen, W. X. Wu, X. Y. Liang and B. Y. Chen | PQ330590 | PQ338457 | N/A | N/A |  |  |
| *C. pseudoreteaudii* | CSF24804 | AA-- | Soil (*Eucalyptus* plantation) | E | 21º8'34.99"N, 110º5'32.03"E | S. F. Chen, W. X. Wu, X. Y. Liang and B. Y. Chen | PQ330591 | PQ338458 | N/A | N/A |  |  |
| *C. pseudoreteaudii* | CSF24805 | AA-- | Soil (*Eucalyptus* plantation) | E | 21º8'34.99"N, 110º5'32.03"E | S. F. Chen, W. X. Wu, X. Y. Liang and B. Y. Chen | PQ330592 | PQ338459 | N/A | N/A |  |  |
| *C. pseudoreteaudii* | CSF24806 | AAAA | Soil (*Eucalyptus* plantation) | E | 21º8'34.99"N, 110º5'32.03"E | S. F. Chen, W. X. Wu, X. Y. Liang and B. Y. Chen | PQ330593 | PQ338460 | PQ330795 | PQ338662 |  |  |
| *C. pseudoreteaudii* | CSF24807 | AA-- | Soil (*Eucalyptus* plantation) | E | 21º8'34.99"N, 110º5'32.03"E | S. F. Chen, W. X. Wu, X. Y. Liang and B. Y. Chen | PQ330594 | PQ338461 | N/A | N/A |  |  |
| *C. pseudoreteaudii* | CSF24808 | AA-- | Soil (*Eucalyptus* plantation) | E | 21º8'34.99"N, 110º5'32.03"E | S. F. Chen, W. X. Wu, X. Y. Liang and B. Y. Chen | PQ330595 | PQ338462 | N/A | N/A |  |  |
| *C. pseudoreteaudii* | CSF24809 | AA-- | Soil (*Eucalyptus* plantation) | E | 21º8'34.99"N, 110º5'32.03"E | S. F. Chen, W. X. Wu, X. Y. Liang and B. Y. Chen | PQ330596 | PQ338463 | N/A | N/A |  |  |
| *C. pseudoreteaudii* | CSF24811 | AA-- | Soil (*Eucalyptus* plantation) | E | 21º8'34.99"N, 110º5'32.03"E | S. F. Chen, W. X. Wu, X. Y. Liang and B. Y. Chen | PQ330597 | PQ338464 | N/A | N/A |  |  |
| *C. pseudoreteaudii* | CSF24812 | AAAA | Soil (*Eucalyptus* plantation) | E | 21º8'34.99"N, 110º5'32.03"E | S. F. Chen, W. X. Wu, X. Y. Liang and B. Y. Chen | PQ330598 | PQ338465 | PQ330796 | PQ338663 |  |  |
| *C. pseudoreteaudii* | CSF24813 | AA-- | Soil (*Eucalyptus* plantation) | E | 21º8'34.99"N, 110º5'32.03"E | S. F. Chen, W. X. Wu, X. Y. Liang and B. Y. Chen | PQ330599 | PQ338466 | N/A | N/A |  |  |
| *C. pseudoreteaudii* | CSF24814 | AA-- | Soil (*Eucalyptus* plantation) | E | 21º8'34.99"N, 110º5'32.03"E | S. F. Chen, W. X. Wu, X. Y. Liang and B. Y. Chen | PQ330600 | PQ338467 | N/A | N/A |  |  |
| *C. pseudoreteaudii* | CSF24817 | AA-- | Soil (*Eucalyptus* plantation) | E | 21º8'34.99"N, 110º5'32.03"E | S. F. Chen, W. X. Wu, X. Y. Liang and B. Y. Chen | PQ330601 | PQ338468 | N/A | N/A |  |  |
| *C. pseudoreteaudii* | CSF24818 | AAAA | Soil (*Eucalyptus* plantation) | E | 21º8'34.99"N, 110º5'32.03"E | S. F. Chen, W. X. Wu, X. Y. Liang and B. Y. Chen | PQ330602 | PQ338469 | PQ330797 | PQ338664 |  |  |
| *C. pseudoreteaudii* | CSF24819 | AA-- | Soil (*Eucalyptus* plantation) | E | 21º8'34.99"N, 110º5'32.03"E | S. F. Chen, W. X. Wu, X. Y. Liang and B. Y. Chen | PQ330603 | PQ338470 | N/A | N/A |  |  |
| *C. pseudoreteaudii* | CSF24821 | AA-- | Soil (*Eucalyptus* plantation) | E | 21º8'34.99"N, 110º5'32.03"E | S. F. Chen, W. X. Wu, X. Y. Liang and B. Y. Chen | PQ330604 | PQ338471 | N/A | N/A |  |  |
| *C. pseudoreteaudii* | CSF24822 | AA-- | Soil (*Eucalyptus* plantation) | E | 21º8'34.99"N, 110º5'32.03"E | S. F. Chen, W. X. Wu, X. Y. Liang and B. Y. Chen | PQ330605 | PQ338472 | N/A | N/A |  |  |
| *C. pseudoreteaudii* | CSF24823 | AAAA | Soil (*Eucalyptus* plantation) | E | 21º8'34.99"N, 110º5'32.03"E | S. F. Chen, W. X. Wu, X. Y. Liang and B. Y. Chen | PQ330606 | PQ338473 | PQ330798 | PQ338665 |  |  |
| *C. pseudoreteaudii* | CSF24651 | AAAA | 1-year-old *Eucalyptus* leaf | F | 21º8'1.59"N, 110º4'37.57"E | S. F. Chen, W. X. Wu, X. Y. Liang and B. Y. Chen | PQ330607 | PQ338474 | PQ330799 | PQ338666 |  |  |
| *C. pseudoreteaudii* | CSF24653 | AA-- | 1-year-old *Eucalyptus* leaf | F | 21º8'1.59"N, 110º4'37.57"E | S. F. Chen, W. X. Wu, X. Y. Liang and B. Y. Chen | PQ330608 | PQ338475 | N/A | N/A |  |  |
| *C. pseudoreteaudii* | CSF24655 | AA-- | 1-year-old *Eucalyptus* leaf | F | 21º8'1.59"N, 110º4'37.57"E | S. F. Chen, W. X. Wu, X. Y. Liang and B. Y. Chen | PQ330609 | PQ338476 | N/A | N/A |  |  |
| *C. pseudoreteaudii* | CSF24657 | AA-- | 1-year-old *Eucalyptus* leaf | F | 21º8'1.59"N, 110º4'37.57"E | S. F. Chen, W. X. Wu, X. Y. Liang and B. Y. Chen | PQ330610 | PQ338477 | N/A | N/A |  |  |
| *C. pseudoreteaudii* | CSF24659 | AAAA | 1-year-old *Eucalyptus* leaf | F | 21º8'1.59"N, 110º4'37.57"E | S. F. Chen, W. X. Wu, X. Y. Liang and B. Y. Chen | PQ330611 | PQ338478 | PQ330800 | PQ338667 |  |  |
| *C. pseudoreteaudii* | CSF24661 | AA-- | 1-year-old *Eucalyptus* leaf | F | 21º8'1.59"N, 110º4'37.57"E | S. F. Chen, W. X. Wu, X. Y. Liang and B. Y. Chen | PQ330612 | PQ338479 | N/A | N/A |  |  |
| *C. pseudoreteaudii* | CSF24663 | AA-- | 1-year-old *Eucalyptus* leaf | F | 21º8'1.59"N, 110º4'37.57"E | S. F. Chen, W. X. Wu, X. Y. Liang and B. Y. Chen | PQ330613 | PQ338480 | N/A | N/A |  |  |
| *C. pseudoreteaudii* | CSF24665 | AA-- | 1-year-old *Eucalyptus* leaf | F | 21º8'1.59"N, 110º4'37.57"E | S. F. Chen, W. X. Wu, X. Y. Liang and B. Y. Chen | PQ330614 | PQ338481 | N/A | N/A |  |  |
| *C. pseudoreteaudii* | CSF24667 | AAAA | 1-year-old *Eucalyptus* leaf | F | 21º8'1.59"N, 110º4'37.57"E | S. F. Chen, W. X. Wu, X. Y. Liang and B. Y. Chen | PQ330615 | PQ338482 | PQ330801 | PQ338668 |  |  |
| *C. pseudoreteaudii* | CSF24669 | AA-- | 1-year-old *Eucalyptus* leaf | F | 21º8'1.59"N, 110º4'37.57"E | S. F. Chen, W. X. Wu, X. Y. Liang and B. Y. Chen | PQ330616 | PQ338483 | N/A | N/A |  |  |
| *C. pseudoreteaudii* | CSF24671 | AA-- | 1-year-old *Eucalyptus* leaf | F | 21º8'1.59"N, 110º4'37.57"E | S. F. Chen, W. X. Wu, X. Y. Liang and B. Y. Chen | PQ330617 | PQ338484 | N/A | N/A |  |  |
| *C. pseudoreteaudii* | CSF24673 | AA-- | 1-year-old *Eucalyptus* leaf | F | 21º8'1.59"N, 110º4'37.57"E | S. F. Chen, W. X. Wu, X. Y. Liang and B. Y. Chen | PQ330618 | PQ338485 | N/A | N/A |  |  |
| *C. pseudoreteaudii* | CSF24676 | AAAA | 1-year-old *Eucalyptus* leaf | F | 21º8'1.59"N, 110º4'37.57"E | S. F. Chen, W. X. Wu, X. Y. Liang and B. Y. Chen | PQ330619 | PQ338486 | PQ330802 | PQ338669 |  |  |
| *C. pseudoreteaudii* | CSF24679 | AA-- | 1-year-old *Eucalyptus* leaf | F | 21º8'1.59"N, 110º4'37.57"E | S. F. Chen, W. X. Wu, X. Y. Liang and B. Y. Chen | PQ330620 | PQ338487 | N/A | N/A |  |  |
| *C. pseudoreteaudii* | CSF24680 | AA-- | 1-year-old *Eucalyptus* leaf | F | 21º8'1.59"N, 110º4'37.57"E | S. F. Chen, W. X. Wu, X. Y. Liang and B. Y. Chen | PQ330621 | PQ338488 | N/A | N/A |  |  |
| *C. pseudoreteaudii* | CSF24681 | AA-- | 1-year-old *Eucalyptus* leaf | F | 21º8'1.59"N, 110º4'37.57"E | S. F. Chen, W. X. Wu, X. Y. Liang and B. Y. Chen | PQ330622 | PQ338489 | N/A | N/A |  |  |
| *C. pseudoreteaudii* | CSF24683 | AA-- | 1-year-old *Eucalyptus* leaf | F | 21º8'1.59"N, 110º4'37.57"E | S. F. Chen, W. X. Wu, X. Y. Liang and B. Y. Chen | PQ330623 | PQ338490 | N/A | N/A |  |  |
| *C. pseudoreteaudii* | CSF24685 | AA-- | 1-year-old *Eucalyptus* leaf | F | 21º8'1.59"N, 110º4'37.57"E | S. F. Chen, W. X. Wu, X. Y. Liang and B. Y. Chen | PQ330624 | PQ338491 | N/A | N/A |  |  |
| *C. pseudoreteaudii* | CSF24687 | AAAA | 1-year-old *Eucalyptus* leaf | F | 21º8'1.59"N, 110º4'37.57"E | S. F. Chen, W. X. Wu, X. Y. Liang and B. Y. Chen | PQ330625 | PQ338492 | PQ330803 | PQ338670 |  |  |
| *C. pseudoreteaudii* | CSF24689 | AA-- | 1-year-old *Eucalyptus* leaf | F | 21º8'1.59"N, 110º4'37.57"E | S. F. Chen, W. X. Wu, X. Y. Liang and B. Y. Chen | PQ330626 | PQ338493 | N/A | N/A |  |  |
| *C. pseudoreteaudii* | CSF24690 | AAAA | Soil (*Eucalyptus* plantation) | F | 21º8'1.59"N, 110º4'37.57"E | S. F. Chen, W. X. Wu, X. Y. Liang and B. Y. Chen | PQ330627 | PQ338494 | PQ330804 | PQ338671 |  |  |
| *C. pseudoreteaudii* | CSF24691 | AA-- | Soil (*Eucalyptus* plantation) | F | 21º8'1.59"N, 110º4'37.57"E | S. F. Chen, W. X. Wu, X. Y. Liang and B. Y. Chen | PQ330628 | PQ338495 | N/A | N/A |  |  |
| *C. pseudoreteaudii* | CSF24692 | AA-- | Soil (*Eucalyptus* plantation) | F | 21º8'1.59"N, 110º4'37.57"E | S. F. Chen, W. X. Wu, X. Y. Liang and B. Y. Chen | PQ330629 | PQ338496 | N/A | N/A |  |  |
| *C. pseudoreteaudii* | CSF24693 | AA-- | Soil (*Eucalyptus* plantation) | F | 21º8'1.59"N, 110º4'37.57"E | S. F. Chen, W. X. Wu, X. Y. Liang and B. Y. Chen | PQ330630 | PQ338497 | N/A | N/A |  |  |
| *C. pseudoreteaudii* | CSF24694 | AAAA | Soil (*Eucalyptus* plantation) | F | 21º8'1.59"N, 110º4'37.57"E | S. F. Chen, W. X. Wu, X. Y. Liang and B. Y. Chen | PQ330631 | PQ338498 | PQ330805 | PQ338672 |  |  |
| *C. pseudoreteaudii* | CSF24695 | AAAA | 1-year-old *Eucalyptus* leaf | G | 21º8'23.20"N, 110º5'45.41"E | S. F. Chen, W. X. Wu, X. Y. Liang and B. Y. Chen | PQ330632 | PQ338499 | PQ330806 | PQ338673 |  |  |
| *C. pseudoreteaudii* | CSF24697 | AA-- | 1-year-old *Eucalyptus* leaf | G | 21º8'23.20"N, 110º5'45.41"E | S. F. Chen, W. X. Wu, X. Y. Liang and B. Y. Chen | PQ330633 | PQ338500 | N/A | N/A |  |  |
| *C. pseudoreteaudii* | CSF24699 | AA-- | 1-year-old *Eucalyptus* leaf | G | 21º8'23.20"N, 110º5'45.41"E | S. F. Chen, W. X. Wu, X. Y. Liang and B. Y. Chen | PQ330634 | PQ338501 | N/A | N/A |  |  |
| *C. pseudoreteaudii* | CSF24701 | AA-- | 1-year-old *Eucalyptus* leaf | G | 21º8'23.20"N, 110º5'45.41"E | S. F. Chen, W. X. Wu, X. Y. Liang and B. Y. Chen | PQ330635 | PQ338502 | N/A | N/A |  |  |
| *C. pseudoreteaudii* | CSF24703 | AABA | 1-year-old *Eucalyptus* leaf | G | 21º8'23.20"N, 110º5'45.41"E | S. F. Chen, W. X. Wu, X. Y. Liang and B. Y. Chen | PQ330636 | PQ338503 | PQ330807 | PQ338674 |  |  |
| *C. pseudoreteaudii* | CSF24706 | AA-- | 1-year-old *Eucalyptus* leaf | G | 21º8'23.20"N, 110º5'45.41"E | S. F. Chen, W. X. Wu, X. Y. Liang and B. Y. Chen | PQ330637 | PQ338504 | N/A | N/A |  |  |
| *C. pseudoreteaudii* | CSF24710 | AA-- | 1-year-old *Eucalyptus* leaf | G | 21º8'23.20"N, 110º5'45.41"E | S. F. Chen, W. X. Wu, X. Y. Liang and B. Y. Chen | PQ330638 | PQ338505 | N/A | N/A |  |  |
| *C. pseudoreteaudii* | CSF24712 | AA-- | 1-year-old *Eucalyptus* leaf | G | 21º8'23.20"N, 110º5'45.41"E | S. F. Chen, W. X. Wu, X. Y. Liang and B. Y. Chen | PQ330639 | PQ338506 | N/A | N/A |  |  |
| *C. pseudoreteaudii* | CSF24714 | AAAA | 1-year-old *Eucalyptus* leaf | G | 21º8'23.20"N, 110º5'45.41"E | S. F. Chen, W. X. Wu, X. Y. Liang and B. Y. Chen | PQ330640 | PQ338507 | PQ330808 | PQ338675 |  |  |
| *C. pseudoreteaudii* | CSF24716 | AA-- | 1-year-old *Eucalyptus* leaf | G | 21º8'23.20"N, 110º5'45.41"E | S. F. Chen, W. X. Wu, X. Y. Liang and B. Y. Chen | PQ330641 | PQ338508 | N/A | N/A |  |  |
| *C. pseudoreteaudii* | CSF24718 | AA-- | 1-year-old *Eucalyptus* leaf | G | 21º8'23.20"N, 110º5'45.41"E | S. F. Chen, W. X. Wu, X. Y. Liang and B. Y. Chen | PQ330642 | PQ338509 | N/A | N/A |  |  |
| *C. pseudoreteaudii* | CSF24720 | AA-- | 1-year-old *Eucalyptus* leaf | G | 21º8'23.20"N, 110º5'45.41"E | S. F. Chen, W. X. Wu, X. Y. Liang and B. Y. Chen | PQ330643 | PQ338510 | N/A | N/A |  |  |
| *C. pseudoreteaudii* | CSF24723 | AAAA | 1-year-old *Eucalyptus* leaf | G | 21º8'23.20"N, 110º5'45.41"E | S. F. Chen, W. X. Wu, X. Y. Liang and B. Y. Chen | PQ330644 | PQ338511 | PQ330809 | PQ338676 |  |  |
| *C. pseudoreteaudii* | CSF24726 | AA-- | 1-year-old *Eucalyptus* leaf | G | 21º8'23.20"N, 110º5'45.41"E | S. F. Chen, W. X. Wu, X. Y. Liang and B. Y. Chen | PQ330645 | PQ338512 | N/A | N/A |  |  |
| *C. pseudoreteaudii* | CSF24729 | AA-- | 1-year-old *Eucalyptus* leaf | G | 21º8'23.20"N, 110º5'45.41"E | S. F. Chen, W. X. Wu, X. Y. Liang and B. Y. Chen | PQ330646 | PQ338513 | N/A | N/A |  |  |
| *C. pseudoreteaudii* | CSF24732 | AA-- | 1-year-old *Eucalyptus* leaf | G | 21º8'23.20"N, 110º5'45.41"E | S. F. Chen, W. X. Wu, X. Y. Liang and B. Y. Chen | PQ330647 | PQ338514 | N/A | N/A |  |  |
| *C. pseudoreteaudii* | CSF24734 | AA-- | 1-year-old *Eucalyptus* leaf | G | 21º8'23.20"N, 110º5'45.41"E | S. F. Chen, W. X. Wu, X. Y. Liang and B. Y. Chen | PQ330648 | PQ338515 | N/A | N/A |  |  |
| *C. pseudoreteaudii* | CSF24737 | AA-- | 1-year-old *Eucalyptus* leaf | G | 21º8'23.20"N, 110º5'45.41"E | S. F. Chen, W. X. Wu, X. Y. Liang and B. Y. Chen | PQ330649 | PQ338516 | N/A | N/A |  |  |
| *C. pseudoreteaudii* | CSF24739 | AAAA | 1-year-old *Eucalyptus* leaf | G | 21º8'23.20"N, 110º5'45.41"E | S. F. Chen, W. X. Wu, X. Y. Liang and B. Y. Chen | PQ330650 | PQ338517 | PQ330810 | PQ338677 |  |  |
| *C. pseudoreteaudii* | CSF24742 | AA-- | 1-year-old *Eucalyptus* leaf | G | 21º8'23.20"N, 110º5'45.41"E | S. F. Chen, W. X. Wu, X. Y. Liang and B. Y. Chen | PQ330651 | PQ338518 | N/A | N/A |  |  |
| *C. pseudoreteaudii* | CSF24744 | AAAA | Soil (*Eucalyptus* plantation) | G | 21º8'23.20"N, 110º5'45.41"E | S. F. Chen, W. X. Wu, X. Y. Liang and B. Y. Chen | PQ330652 | PQ338519 | PQ330811 | PQ338678 |  |  |
| *C. pseudoreteaudii* | **CSF24064 ^e^** | BAAA | 1-year-old *E. urophylla × E. grandis* leaf | H | 19º47'41.46"N, 109º49'52.71"E | S. F. Chen, Q. C. Wang, X. Y. Liang and L. F. Liu | *OQ188170* | *OQ210529* | *OQ210673* | *OQ230744* |  |  |
| *C. pseudoreteaudii* | **CSF24065** | BA-- | 1-year-old *E. urophylla × E. grandis* leaf | H | 19º47'41.46"N, 109º49'52.71"E | S. F. Chen, Q. C. Wang, X. Y. Liang and L. F. Liu | *OQ188172* | *OQ210531* | N/A | N/A |  |  |
| *C. pseudoreteaudii* | **CSF24066** | BA-- | 1-year-old *E. urophylla × E. grandis* leaf | H | 19º47'41.46"N, 109º49'52.71"E | S. F. Chen, Q. C. Wang, X. Y. Liang and L. F. Liu | *OQ188173* | *OQ210532* | N/A | N/A |  |  |
| *C. pseudoreteaudii* | **CSF24067** | BA-- | 1-year-old *E. urophylla × E. grandis* leaf | H | 19º47'41.46"N, 109º49'52.71"E | S. F. Chen, Q. C. Wang, X. Y. Liang and L. F. Liu | *OQ188174* | *OQ210533* | N/A | N/A |  |  |
| *C. pseudoreteaudii* | **CSF24068** | AA-- | 1-year-old *E. urophylla × E. grandis* leaf | H | 19º47'41.46"N, 109º49'52.71"E | S. F. Chen, Q. C. Wang, X. Y. Liang and L. F. Liu | *OQ188139* | *OQ210499* | N/A | N/A |  |  |
| *C. pseudoreteaudii* | **CSF24069** | AA-- | 1-year-old *E. urophylla × E. grandis* leaf | H | 19º47'41.46"N, 109º49'52.71"E | S. F. Chen, Q. C. Wang, X. Y. Liang and L. F. Liu | *OQ188140* | *OQ210500* | N/A | N/A |  |  |
| *C. pseudoreteaudii* | **CSF24070** | AA-- | 1-year-old *E. urophylla × E. grandis* leaf | H | 19º47'41.46"N, 109º49'52.71"E | S. F. Chen, Q. C. Wang, X. Y. Liang and L. F. Liu | *OQ188141* | *OQ210501* | N/A | N/A |  |  |
| *C. pseudoreteaudii* | **CSF24071** | AA-- | 1-year-old *E. urophylla × E. grandis* leaf | H | 19º47'41.46"N, 109º49'52.71"E | S. F. Chen, Q. C. Wang, X. Y. Liang and L. F. Liu | *OQ188142* | *OQ210502* | N/A | N/A |  |  |
| *C. pseudoreteaudii* | **CSF24073** | AAAA | 1-year-old *E. urophylla × E. grandis* leaf | H | 19º47'41.46"N, 109º49'52.71"E | S. F. Chen, Q. C. Wang, X. Y. Liang and L. F. Liu | *OQ188047* | *OQ210407* | *OQ210669* | *OQ230740* |  |  |
| *C. pseudoreteaudii* | **CSF24077** | AA-- | 1-year-old *E. urophylla × E. grandis* leaf | H | 19º47'41.46"N, 109º49'52.71"E | S. F. Chen, Q. C. Wang, X. Y. Liang and L. F. Liu | *OQ188143* | *OQ210503* | N/A | N/A |  |  |
| *C. pseudoreteaudii* | **CSF24079** | AA-- | 1-year-old *E. urophylla × E. grandis* leaf | H | 19º47'41.46"N, 109º49'52.71"E | S. F. Chen, Q. C. Wang, X. Y. Liang and L. F. Liu | *OQ188144* | *OQ210504* | N/A | N/A |  |  |
| *C. pseudoreteaudii* | **CSF24084** | AA-- | 1-year-old *E. urophylla × E. grandis* leaf | H | 19º47'41.46"N, 109º49'52.71"E | S. F. Chen, Q. C. Wang, X. Y. Liang and L. F. Liu | *OQ188145* | *OQ210505* | N/A | N/A |  |  |
| *C. pseudoreteaudii* | **CSF24085** | AA-- | 1-year-old *E. urophylla × E. grandis* leaf | H | 19º47'41.46"N, 109º49'52.71"E | S. F. Chen, Q. C. Wang, X. Y. Liang and L. F. Liu | *OQ188146* | *OQ210506* | N/A | N/A |  |  |
| *C. pseudoreteaudii* | **CSF24086** | AA-- | 1-year-old *E. urophylla × E. grandis* leaf | H | 19º47'41.46"N, 109º49'52.71"E | S. F. Chen, Q. C. Wang, X. Y. Liang and L. F. Liu | *OQ188147* | *OQ210507* | N/A | N/A |  |  |
| *C. pseudoreteaudii* | **CSF24087** | AA-- | 1-year-old *E. urophylla × E. grandis* leaf | H | 19º47'41.46"N, 109º49'52.71"E | S. F. Chen, Q. C. Wang, X. Y. Liang and L. F. Liu | *OQ188148* | *OQ210508* | N/A | N/A |  |  |
| *C. pseudoreteaudii* | **CSF24092** | AA-- | 1-year-old *E. urophylla × E. grandis* leaf | H | 19º47'41.46"N, 109º49'52.71"E | S. F. Chen, Q. C. Wang, X. Y. Liang and L. F. Liu | *OQ188149* | *OQ210509* | N/A | N/A |  |  |
| *C. pseudoreteaudii* | **CSF24093** | AA-- | 1-year-old *E. urophylla × E. grandis* leaf | H | 19º47'41.46"N, 109º49'52.71"E | S. F. Chen, Q. C. Wang, X. Y. Liang and L. F. Liu | *OQ188150* | *OQ210510* | N/A | N/A |  |  |
| *C. pseudoreteaudii* | **CSF24094** | AA-- | 1-year-old *E. urophylla × E. grandis* leaf | H | 19º47'41.46"N, 109º49'52.71"E | S. F. Chen, Q. C. Wang, X. Y. Liang and L. F. Liu | *OQ188151* | *OQ210511* | N/A | N/A |  |  |
| *C. pseudoreteaudii* | **CSF24095** | AA-- | 1-year-old *E. urophylla × E. grandis* leaf | H | 19º47'41.46"N, 109º49'52.71"E | S. F. Chen, Q. C. Wang, X. Y. Liang and L. F. Liu | *OQ188152* | *OQ210512* | N/A | N/A |  |  |
| *C. pseudoreteaudii* | **CSF24096** | AA-- | 1-year-old *E. urophylla × E. grandis* leaf | H | 19º47'41.46"N, 109º49'52.71"E | S. F. Chen, Q. C. Wang, X. Y. Liang and L. F. Liu | *OQ188153* | *OQ210513* | N/A | N/A |  |  |
| *C. pseudoreteaudii* | **CSF24101** | AA-- | 1-year-old *E. urophylla × E. grandis* leaf | H | 19º47'41.46"N, 109º49'52.71"E | S. F. Chen, Q. C. Wang, X. Y. Liang and L. F. Liu | *OQ188154* | *OQ210514* | N/A | N/A |  |  |
| *C. pseudoreteaudii* | **CSF24103** | AA-- | 1-year-old *E. urophylla × E. grandis* leaf | H | 19º47'41.46"N, 109º49'52.71"E | S. F. Chen, Q. C. Wang, X. Y. Liang and L. F. Liu | *OQ188155* | *OQ210515* | N/A | N/A |  |  |
| *C. pseudoreteaudii* | **CSF24104** | AA-- | 1-year-old *E. urophylla × E. grandis* leaf | H | 19º47'41.46"N, 109º49'52.71"E | S. F. Chen, Q. C. Wang, X. Y. Liang and L. F. Liu | *OQ188156* | *OQ210516* | N/A | N/A |  |  |
| *C. pseudoreteaudii* | **CSF24105** | AA-- | 1-year-old *E. urophylla × E. grandis* leaf | H | 19º47'41.46"N, 109º49'52.71"E | S. F. Chen, Q. C. Wang, X. Y. Liang and L. F. Liu | *OQ188157* | *OQ210517* | N/A | N/A |  |  |
| *C. pseudoreteaudii* | **CSF24106** | AA-- | 1-year-old *E. urophylla × E. grandis* leaf | H | 19º47'41.46"N, 109º49'52.71"E | S. F. Chen, Q. C. Wang, X. Y. Liang and L. F. Liu | *OQ188158* | *OQ210518* | N/A | N/A |  |  |
| *C. pseudoreteaudii* | **CSF24107** | AA-- | 1-year-old *E. urophylla × E. grandis* leaf | H | 19º47'41.46"N, 109º49'52.71"E | S. F. Chen, Q. C. Wang, X. Y. Liang and L. F. Liu | *OQ188159* | *OQ210519* | N/A | N/A |  |  |
| *C. pseudoreteaudii* | **CSF24108** | AA-- | 1-year-old *E. urophylla × E. grandis* leaf | H | 19º47'41.46"N, 109º49'52.71"E | S. F. Chen, Q. C. Wang, X. Y. Liang and L. F. Liu | *OQ188160* | *OQ210520* | N/A | N/A |  |  |
| *C. pseudoreteaudii* | **CSF24109** | AA-- | 1-year-old *E. urophylla × E. grandis* leaf | H | 19º47'41.46"N, 109º49'52.71"E | S. F. Chen, Q. C. Wang, X. Y. Liang and L. F. Liu | *OQ188161* | *OQ210521* | N/A | N/A |  |  |
| *C. pseudoreteaudii* | **CSF24110** | AA-- | 1-year-old *E. urophylla × E. grandis* leaf | H | 19º47'41.46"N, 109º49'52.71"E | S. F. Chen, Q. C. Wang, X. Y. Liang and L. F. Liu | *OQ188162* | *OQ210522* | N/A | N/A |  |  |
| *C. pseudoreteaudii* | **CSF24111** | BA-- | 1-year-old *E. urophylla × E. grandis* leaf | H | 19º47'41.46"N, 109º49'52.71"E | S. F. Chen, Q. C. Wang, X. Y. Liang and L. F. Liu | *OQ188175* | *OQ210534* | N/A | N/A |  |  |
| *C. pseudoreteaudii* | **CSF24114** | AA-- | 1-year-old *E. urophylla × E. grandis* leaf | H | 19º47'41.46"N, 109º49'52.71"E | S. F. Chen, Q. C. Wang, X. Y. Liang and L. F. Liu | *OQ188163* | *OQ210523* | N/A | N/A |  |  |
| *C. pseudoreteaudii* | **CSF24116 ^e^** | BAAA | 1-year-old *E. urophylla × E. grandis* leaf | H | 19º47'41.46"N, 109º49'52.71"E | S. F. Chen, Q. C. Wang, X. Y. Liang and L. F. Liu | *OQ188171* | *OQ210530* | *OQ210674* | *OQ230745* |  |  |
| *C. pseudoreteaudii* | **CSF24117** | BA-- | 1-year-old *E. urophylla × E. grandis* leaf | H | 19º47'41.46"N, 109º49'52.71"E | S. F. Chen, Q. C. Wang, X. Y. Liang and L. F. Liu | *OQ188176* | *OQ210535* | N/A | N/A |  |  |
| *C. pseudoreteaudii* | CSF24824 | BAAA | Soil (*Eucalyptus* plantation) | H | 19º47'41.46"N, 109º49'52.71"E | S. F. Chen, Q. C. Wang, X. Y. Liang and L. F. Liu | PQ330653 | PQ338520 | PQ330812 | PQ338679 |  |  |
| *C. pseudoreteaudii* | CSF24825 | BAAA | Soil (*Eucalyptus* plantation) | H | 19º47'41.46"N, 109º49'52.71"E | S. F. Chen, Q. C. Wang, X. Y. Liang and L. F. Liu | PQ330654 | PQ338521 | PQ330813 | PQ338680 |  |  |
| *C. pseudoreteaudii* | CSF24826 | BAAA | Soil (*Eucalyptus* plantation) | H | 19º47'41.46"N, 109º49'52.71"E | S. F. Chen, Q. C. Wang, X. Y. Liang and L. F. Liu | PQ330655 | PQ338522 | PQ330814 | PQ338681 |  |  |
| *C. pseudoreteaudii* | CSF24827 | AAAA | Soil (*Eucalyptus* plantation) | H | 19º47'41.46"N, 109º49'52.71"E | S. F. Chen, Q. C. Wang, X. Y. Liang and L. F. Liu | PQ330656 | PQ338523 | PQ330815 | PQ338682 |  |  |
| *C. pseudoreteaudii* | CSF24828 | AAAA | Soil (*Eucalyptus* plantation) | H | 19º47'41.46"N, 109º49'52.71"E | S. F. Chen, Q. C. Wang, X. Y. Liang and L. F. Liu | PQ330657 | PQ338524 | PQ330816 | PQ338683 |  |  |
| *C. pseudoreteaudii* | CSF24829 | AAAA | Soil (*Eucalyptus* plantation) | H | 19º47'41.46"N, 109º49'52.71"E | S. F. Chen, Q. C. Wang, X. Y. Liang and L. F. Liu | PQ330658 | PQ338525 | PQ330817 | PQ338684 |  |  |
| *C. pseudoreteaudii* | CSF24831 | AAAA | Soil (*Eucalyptus* plantation) | H | 19º47'41.46"N, 109º49'52.71"E | S. F. Chen, Q. C. Wang, X. Y. Liang and L. F. Liu | PQ330659 | PQ338526 | PQ330818 | PQ338685 |  |  |
| *C. pseudoreteaudii* | CSF24833 | AAAA | Soil (*Eucalyptus* plantation) | H | 19º47'41.46"N, 109º49'52.71"E | S. F. Chen, Q. C. Wang, X. Y. Liang and L. F. Liu | PQ330660 | PQ338527 | PQ330819 | PQ338686 |  |  |
| *C. pseudoreteaudii* | CSF24834 | AAAA | Soil (*Eucalyptus* plantation) | H | 19º47'41.46"N, 109º49'52.71"E | S. F. Chen, Q. C. Wang, X. Y. Liang and L. F. Liu | PQ330661 | PQ338528 | PQ330820 | PQ338687 |  |  |
| *C. pseudoreteaudii* | CSF24835 | AAAA | Soil (*Eucalyptus* plantation) | H | 19º47'41.46"N, 109º49'52.71"E | S. F. Chen, Q. C. Wang, X. Y. Liang and L. F. Liu | PQ330662 | PQ338529 | PQ330821 | PQ338688 |  |  |
| *C. pseudoreteaudii* | CSF24841 | AAAA | Soil (*Eucalyptus* plantation) | H | 19º47'41.46"N, 109º49'52.71"E | S. F. Chen, Q. C. Wang, X. Y. Liang and L. F. Liu | PQ330663 | PQ338530 | PQ330822 | PQ338689 |  |  |
| *C. pseudoreteaudii* | CSF24848 | AAAA | Soil (*Eucalyptus* plantation) | H | 19º47'41.46"N, 109º49'52.71"E | S. F. Chen, Q. C. Wang, X. Y. Liang and L. F. Liu | PQ330664 | PQ338531 | PQ330823 | PQ338690 |  |  |
| *C. pseudoreteaudii* | CSF24854 | AAAA | Soil (*Eucalyptus* plantation) | H | 19º47'41.46"N, 109º49'52.71"E | S. F. Chen, Q. C. Wang, X. Y. Liang and L. F. Liu | PQ330665 | PQ338532 | PQ330824 | PQ338691 |  |  |
| *C. pseudoreteaudii* | CSF24868 | AAAA | Soil (*Eucalyptus* plantation) | H | 19º47'41.46"N, 109º49'52.71"E | S. F. Chen, Q. C. Wang, X. Y. Liang and L. F. Liu | PQ330666 | PQ338533 | PQ330825 | PQ338692 |  |  |
| *C. pseudoreteaudii* | CSF24869 | AAAA | Soil (*Eucalyptus* plantation) | H | 19º47'41.46"N, 109º49'52.71"E | S. F. Chen, Q. C. Wang, X. Y. Liang and L. F. Liu | PQ330667 | PQ338534 | PQ330826 | PQ338693 |  |  |
| *C. pseudoreteaudii* | CSF24870 | AAAA | Soil (*Eucalyptus* plantation) | H | 19º47'41.46"N, 109º49'52.71"E | S. F. Chen, Q. C. Wang, X. Y. Liang and L. F. Liu | PQ330668 | PQ338535 | PQ330827 | PQ338694 |  |  |
| *C. pseudoreteaudii* | CSF24872 | AAAA | Soil (*Eucalyptus* plantation) | H | 19º47'41.46"N, 109º49'52.71"E | S. F. Chen, Q. C. Wang, X. Y. Liang and L. F. Liu | PQ330669 | PQ338536 | PQ330828 | PQ338695 |  |  |
| *C. pseudoreteaudii* | CSF24874 | AAAA | Soil (*Eucalyptus* plantation) | H | 19º47'41.46"N, 109º49'52.71"E | S. F. Chen, Q. C. Wang, X. Y. Liang and L. F. Liu | PQ330670 | PQ338537 | PQ330829 | PQ338696 |  |  |
| *C. pseudoreteaudii* | CSF24875 | AAAA | Soil (*Eucalyptus* plantation) | H | 19º47'41.46"N, 109º49'52.71"E | S. F. Chen, Q. C. Wang, X. Y. Liang and L. F. Liu | PQ330671 | PQ338538 | PQ330830 | PQ338697 |  |  |
| *C. pseudoreteaudii* | CSF24876 | AAAA | Soil (*Eucalyptus* plantation) | H | 19º47'41.46"N, 109º49'52.71"E | S. F. Chen, Q. C. Wang, X. Y. Liang and L. F. Liu | PQ330672 | PQ338539 | PQ330831 | PQ338698 |  |  |
| *C. pseudoreteaudii* | CSF24878 | AAAA | Soil (*Eucalyptus* plantation) | H | 19º47'41.46"N, 109º49'52.71"E | S. F. Chen, Q. C. Wang, X. Y. Liang and L. F. Liu | PQ330673 | PQ338540 | PQ330832 | PQ338699 |  |  |
| *C. pseudoreteaudii* | CSF24880 | AAAA | Soil (*Eucalyptus* plantation) | H | 19º47'41.46"N, 109º49'52.71"E | S. F. Chen, Q. C. Wang, X. Y. Liang and L. F. Liu | PQ330674 | PQ338541 | PQ330833 | PQ338700 |  |  |
| *C. pseudoreteaudii* | CSF24881 | AAAA | Soil (*Eucalyptus* plantation) | H | 19º47'41.46"N, 109º49'52.71"E | S. F. Chen, Q. C. Wang, X. Y. Liang and L. F. Liu | PQ330675 | PQ338542 | PQ330834 | PQ338701 |  |  |
| *C. pseudoreteaudii* | CSF24882 | AAAA | Soil (*Eucalyptus* plantation) | H | 19º47'41.46"N, 109º49'52.71"E | S. F. Chen, Q. C. Wang, X. Y. Liang and L. F. Liu | PQ330676 | PQ338543 | PQ330835 | PQ338702 |  |  |
| *C. pseudoreteaudii* | CSF24884 | AAAA | Soil (*Eucalyptus* plantation) | H | 19º47'41.46"N, 109º49'52.71"E | S. F. Chen, Q. C. Wang, X. Y. Liang and L. F. Liu | PQ330677 | PQ338544 | PQ330836 | PQ338703 |  |  |
| *C. pseudoreteaudii* | CSF24886 | BAAA | Soil (*Eucalyptus* plantation) | H | 19º47'41.46"N, 109º49'52.71"E | S. F. Chen, Q. C. Wang, X. Y. Liang and L. F. Liu | PQ330678 | PQ338545 | PQ330837 | PQ338704 |  |  |
| *C. pseudoreteaudii* | CSF24887 | AAAA | Soil (*Eucalyptus* plantation) | H | 19º47'41.46"N, 109º49'52.71"E | S. F. Chen, Q. C. Wang, X. Y. Liang and L. F. Liu | PQ330679 | PQ338546 | PQ330838 | PQ338705 |  |  |
| *C. pseudoreteaudii* | CSF24889 | BAAA | Soil (*Eucalyptus* plantation) | H | 19º47'41.46"N, 109º49'52.71"E | S. F. Chen, Q. C. Wang, X. Y. Liang and L. F. Liu | PQ330680 | PQ338547 | PQ330839 | PQ338706 |  |  |
| *C. pseudoreteaudii* | CSF24891 | BAAA | Soil (*Eucalyptus* plantation) | H | 19º47'41.46"N, 109º49'52.71"E | S. F. Chen, Q. C. Wang, X. Y. Liang and L. F. Liu | PQ330681 | PQ338548 | PQ330840 | PQ338707 |  |  |
| *Calonectria kyotensis* species complex | | | | | | | | | | | | |
| *C. aconidialis* | CSF24227 ^e^ | AAAA | Soil (*Eucalyptus* plantation) | A | 21º51'39.78"N, 108º49'52.83"E | S. F. Chen, W. X. Wu, X. Y. Liang and B. Y. Chen | PQ330719 | PQ338586 | PQ330878 | PQ338745 |  |  |
| *C. aconidialis* | CSF24231 ^e^ | BAAA | Soil (*Eucalyptus* plantation) | A | 21º51'39.78"N, 108º49'52.83"E | S. F. Chen, W. X. Wu, X. Y. Liang and B. Y. Chen | PQ330720 | PQ338587 | PQ330879 | PQ338746 |  |  |
| *C. aconidialis* | CSF24235 | BAAA | Soil (*Eucalyptus* plantation) | A | 21º51'39.78"N, 108º49'52.83"E | S. F. Chen, W. X. Wu, X. Y. Liang and B. Y. Chen | PQ330721 | PQ338588 | PQ330880 | PQ338747 |  |  |
| *C. aconidialis* | CSF24251 | AAAA | Soil (*Eucalyptus* plantation) | A | 21º51'39.78"N, 108º49'52.83"E | S. F. Chen, W. X. Wu, X. Y. Liang and B. Y. Chen | PQ330722 | PQ338589 | PQ330881 | PQ338748 |  |  |
| *C. aconidialis* | CSF24255 ^e^ | BAAA | Soil (*Eucalyptus* plantation) | A | 21º51'39.78"N, 108º49'52.83"E | S. F. Chen, W. X. Wu, X. Y. Liang and B. Y. Chen | PQ330723 | PQ338590 | PQ330882 | PQ338749 |  |  |
| *C. aconidialis* | CSF24274 | BAAA | Soil (*Eucalyptus* plantation) | A | 21º51'39.78"N, 108º49'52.83"E | S. F. Chen, W. X. Wu, X. Y. Liang and B. Y. Chen | PQ330724 | PQ338591 | PQ330883 | PQ338750 |  |  |
| *C. aconidialis* | CSF24550 ^e^ | AAAA | Soil (*Eucalyptus* plantation) | C | 21º50'31.94"N, 108º57'37.67"E | S. F. Chen, W. X. Wu, X. Y. Liang and B. Y. Chen | PQ330725 | PQ338592 | PQ330884 | PQ338751 |  |  |
| *C. chinensis* | CSF24858 ^e^ | ABAA | Soil (*Eucalyptus* plantation) | H | 19º47'41.46"N, 109º49'52.71"E | S. F. Chen, Q. C. Wang, X. Y. Liang and L. F. Liu | PQ330726 | PQ338593 | PQ330885 | PQ338752 |  |  |
| *C. chinensis* | CSF24865 ^e^ | ACAA | Soil (*Eucalyptus* plantation) | H | 19º47'41.46"N, 109º49'52.71"E | S. F. Chen, Q. C. Wang, X. Y. Liang and L. F. Liu | PQ330727 | PQ338594 | PQ330886 | PQ338753 |  |  |
| *C. chinensis* | CSF24873 ^e^ | ADAA | Soil (*Eucalyptus* plantation) | H | 19º47'41.46"N, 109º49'52.71"E | S. F. Chen, Q. C. Wang, X. Y. Liang and L. F. Liu | PQ330728 | PQ338595 | PQ330887 | PQ338754 |  |  |
| *C. chinensis* | CSF24877 ^e^ | BAAA | Soil (*Eucalyptus* plantation) | H | 19º47'41.46"N, 109º49'52.71"E | S. F. Chen, Q. C. Wang, X. Y. Liang and L. F. Liu | PQ330729 | PQ338596 | PQ330888 | PQ338755 |  |  |
| *C. chinensis* | CSF24879 ^e^ | BAAA | Soil (*Eucalyptus* plantation) | H | 19º47'41.46"N, 109º49'52.71"E | S. F. Chen, Q. C. Wang, X. Y. Liang and L. F. Liu | PQ330730 | PQ338597 | PQ330889 | PQ338756 |  |  |
| *C. hongkongensis* | CSF24618 ^e^ | CAAA | Soil (*Eucalyptus* plantation) | D | 21º50'39.81"N, 108º55'56.89"E | S. F. Chen, W. X. Wu, X. Y. Liang and B. Y. Chen | PQ330703 | PQ338570 | PQ330862 | PQ338729 |  |  |
| *C. hongkongensis* | CSF24630 ^e^ | AAAA | Soil (*Eucalyptus* plantation) | D | 21º50'39.81"N, 108º55'56.89"E | S. F. Chen, W. X. Wu, X. Y. Liang and B. Y. Chen | PQ330704 | PQ338571 | PQ330863 | PQ338730 |  |  |
| *C. hongkongensis* | CSF24830 ^e^ | AABA | Soil (*Eucalyptus* plantation) | H | 19º47'41.46"N, 109º49'52.71"E | S. F. Chen, Q. C. Wang, X. Y. Liang and L. F. Liu | PQ330705 | PQ338572 | PQ330864 | PQ338731 |  |  |
| *C. hongkongensis* | CSF24832 | AABA | Soil (*Eucalyptus* plantation) | H | 19º47'41.46"N, 109º49'52.71"E | S. F. Chen, Q. C. Wang, X. Y. Liang and L. F. Liu | PQ330706 | PQ338573 | PQ330865 | PQ338732 |  |  |
| *C. hongkongensis* | CSF24838 | AAAA | Soil (*Eucalyptus* plantation) | H | 19º47'41.46"N, 109º49'52.71"E | S. F. Chen, Q. C. Wang, X. Y. Liang and L. F. Liu | PQ330707 | PQ338574 | PQ330866 | PQ338733 |  |  |
| *C. hongkongensis* | CSF24840 | AAAA | Soil (*Eucalyptus* plantation) | H | 19º47'41.46"N, 109º49'52.71"E | S. F. Chen, Q. C. Wang, X. Y. Liang and L. F. Liu | PQ330708 | PQ338575 | PQ330867 | PQ338734 |  |  |
| *C. hongkongensis* | CSF24843 ^e^ | ABAA | Soil (*Eucalyptus* plantation) | H | 19º47'41.46"N, 109º49'52.71"E | S. F. Chen, Q. C. Wang, X. Y. Liang and L. F. Liu | PQ330709 | PQ338576 | PQ330868 | PQ338735 |  |  |
| *C. hongkongensis* | CSF24845 | AAAA | Soil (*Eucalyptus* plantation) | H | 19º47'41.46"N, 109º49'52.71"E | S. F. Chen, Q. C. Wang, X. Y. Liang and L. F. Liu | PQ330710 | PQ338577 | PQ330869 | PQ338736 |  |  |
| *C. hongkongensis* | CSF24847 ^e^ | ABAA | Soil (*Eucalyptus* plantation) | H | 19º47'41.46"N, 109º49'52.71"E | S. F. Chen, Q. C. Wang, X. Y. Liang and L. F. Liu | PQ330711 | PQ338578 | PQ330870 | PQ338737 |  |  |
| *C. hongkongensis* | CSF24850 | AAAA | Soil (*Eucalyptus* plantation) | H | 19º47'41.46"N, 109º49'52.71"E | S. F. Chen, Q. C. Wang, X. Y. Liang and L. F. Liu | PQ330712 | PQ338579 | PQ330871 | PQ338738 |  |  |
| *C. hongkongensis* | CSF24852 ^e^ | AABA | Soil (*Eucalyptus* plantation) | H | 19º47'41.46"N, 109º49'52.71"E | S. F. Chen, Q. C. Wang, X. Y. Liang and L. F. Liu | PQ330713 | PQ338580 | PQ330872 | PQ338739 |  |  |
| *C. hongkongensis* | CSF24860 | AAAA | Soil (*Eucalyptus* plantation) | H | 19º47'41.46"N, 109º49'52.71"E | S. F. Chen, Q. C. Wang, X. Y. Liang and L. F. Liu | PQ330714 | PQ338581 | PQ330873 | PQ338740 |  |  |
| *C. hongkongensis* | CSF24862 | AAAA | Soil (*Eucalyptus* plantation) | H | 19º47'41.46"N, 109º49'52.71"E | S. F. Chen, Q. C. Wang, X. Y. Liang and L. F. Liu | PQ330715 | PQ338582 | PQ330874 | PQ338741 |  |  |
| *C. hongkongensis* | CSF24871 ^e^ | AAAA | Soil (*Eucalyptus* plantation) | H | 19º47'41.46"N, 109º49'52.71"E | S. F. Chen, Q. C. Wang, X. Y. Liang and L. F. Liu | PQ330716 | PQ338583 | PQ330875 | PQ338742 |  |  |
| *C. hongkongensis* | CSF24883 ^e^ | BCAA | Soil (*Eucalyptus* plantation) | H | 19º47'41.46"N, 109º49'52.71"E | S. F. Chen, Q. C. Wang, X. Y. Liang and L. F. Liu | PQ330717 | PQ338584 | PQ330876 | PQ338743 |  |  |
| *C. hongkongensis* | CSF24892 | AABA | Soil (*Eucalyptus* plantation) | H | 19º47'41.46"N, 109º49'52.71"E | S. F. Chen, Q. C. Wang, X. Y. Liang and L. F. Liu | PQ330718 | PQ338585 | PQ330877 | PQ338744 |  |  |
| *Calonectria cylindrospora* species complex | | | | | | | | | | | |  |
| *C. auriculiformis* | CSF24816 ^e^ | AAAA | Soil (*Eucalyptus* plantation) | E | 21º8'34.99"N, 110º5'32.03"E | S. F. Chen, Q. C. Wang, X. Y. Liang and L. F. Liu | PQ330731 | PQ338598 | PQ330890 | PQ338757 |  |  |

^a^ CSF: Culture collection located at the Research Institute of Fast-growing Trees (RIFT), Chinese Academy of Forestry, Zhanjiang, Guangdong Province, China.

^b^ Isolates indicated in bold were used by Liang et al. (2023). The relative GenBank accession numbers were in italic.

^c^ Genotype within each *Calonectria* species, as confirmed by sequences of the *tef1*, *tub2*, *cmdA*, and *his3* gene regions.

^d^ *tef1* = translation elongation factor 1-alpha; *tub2* = β-tubulin; *cmdA* = calmodulin; *his3* = histone H3.

^e^ Isolates used for phylogenetic analyses.

f “-”: the relative locus was not sequenced.

^g^ “N/A”: information is not available.
