# Supplementary figures and images for: ﻿Distribution patterns of Calonectria (Ascomycota, Sordariomycetes, Hypocreales, Nectriaceae) species complexes related to diseased leaves and soil habitats during leaf blight outbreak season in Eucalyptus plantations in southern China
Source: MycoKeys. 2024 Nov 4;110:117–40. doi: 10.3897/mycokeys.110.130733 (PMC11555429; doi:10.3897/mycokeys.110.130733)

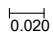

Supplement: Supplementary material 2 — Phylogenetic tree of Calonectria species based on maximum likelihood (ML) analyses of a combined DNA dataset of tef1 gene sequences [file mycokeys-110-117-s002.pdf]

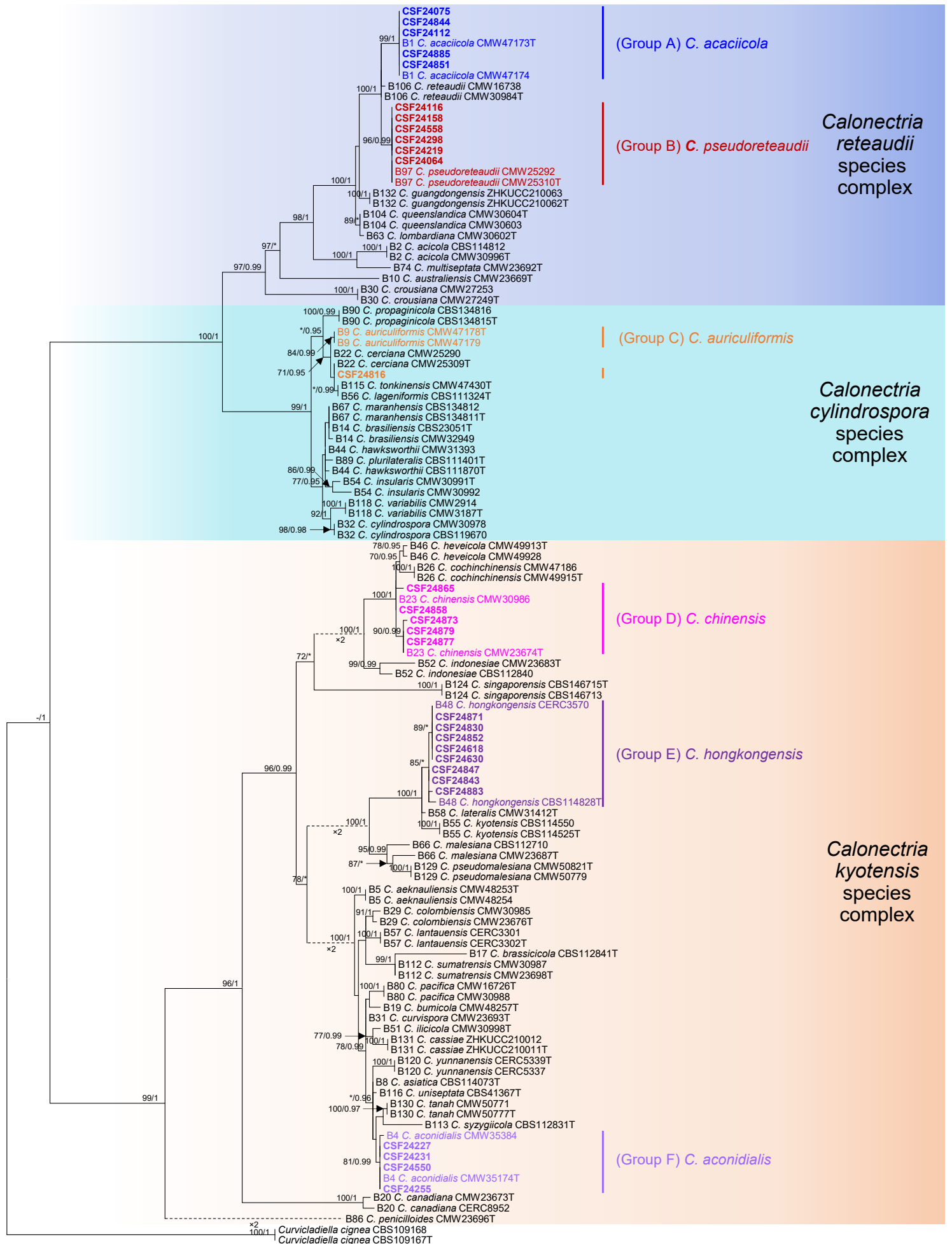

Supplement: Supplementary material 3 — Phylogenetic tree of Calonectria species based on maximum likelihood (ML) analyses of a combined DNA dataset of tub2 gene sequences [file mycokeys-110-117-s003.pdf]

cmdA

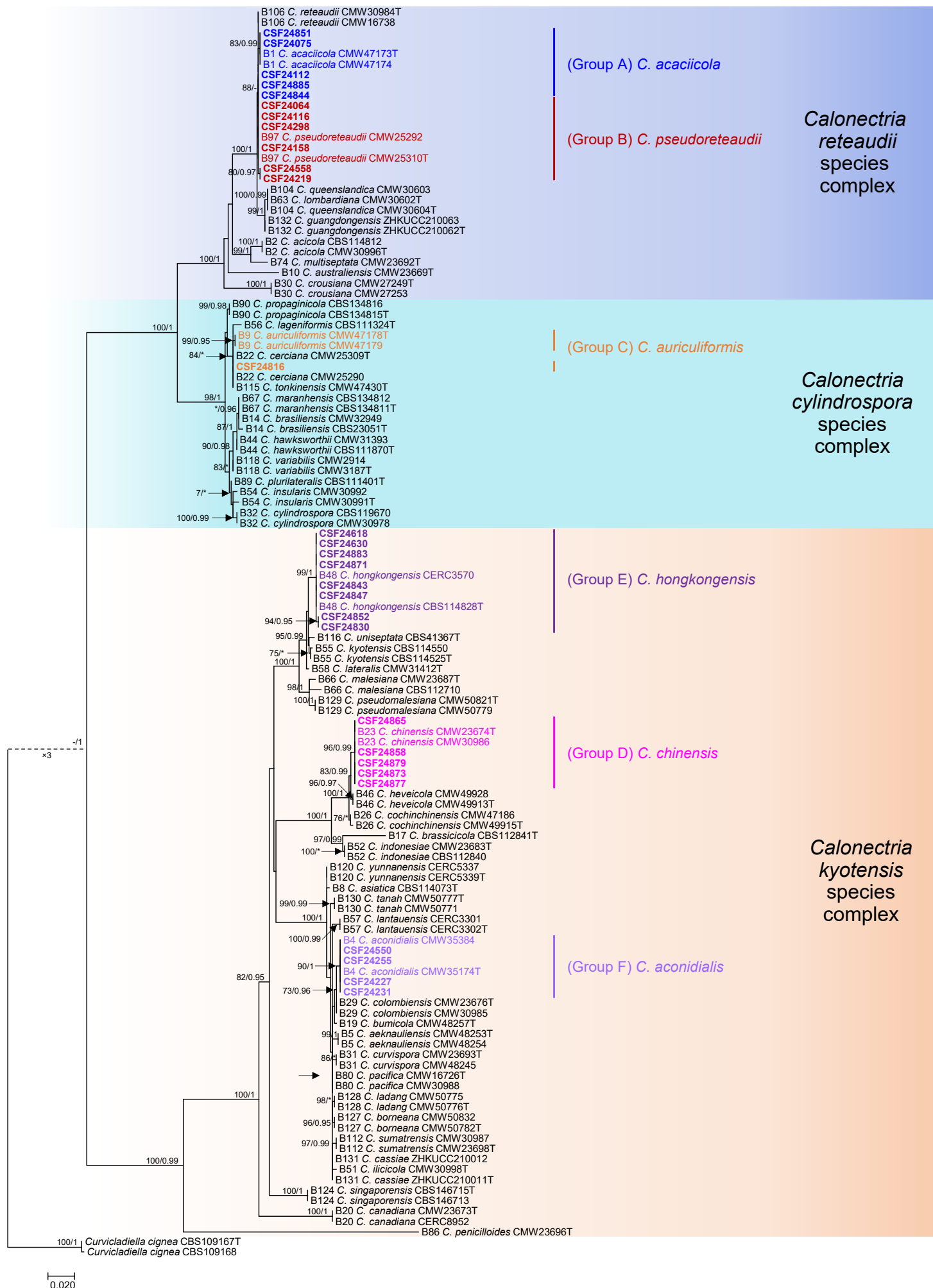

Supplement: Supplementary material 4 — Phylogenetic tree of Calonectria species based on maximum likelihood (ML) analyses of a combined DNA dataset of cmdA gene sequences [file mycokeys-110-117-s004.pdf]

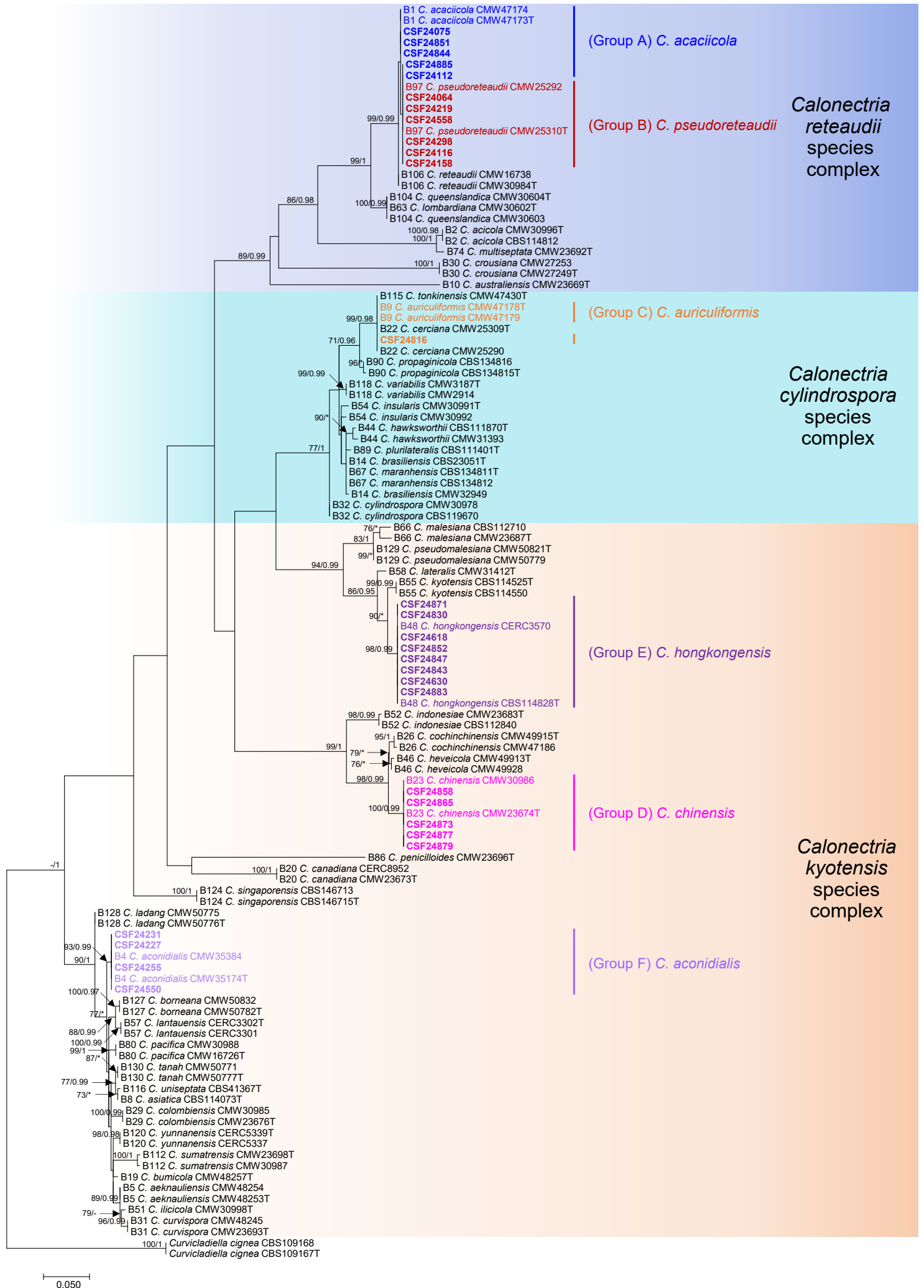

Supplement: Supplementary material 5 — Phylogenetic tree of Calonectria species based on maximum likelihood (ML) analyses of a combined DNA dataset of his3 gene sequences [file mycokeys-110-117-s005.pdf]
